# Evaluation of Novel Cloxyquin Analogs for K_2P_18.1 Channel Modulation

**DOI:** 10.1002/ardp.70310

**Published:** 2026-07-15

**Authors:** Jasmin Sörgel, Marcel Kloth, Henning Klaasen, Sven G. Meuth, Thomas Budde, Bart Jan Ravoo, Julian A. Schreiber

**Affiliations:** ^1^ Institute of Pharmaceutical and Medicinal Chemistry University of Münster Münster NRW Germany; ^2^ Organisch‐Chemisches Institut University of Münster Münster NRW Germany; ^3^ Department of Neurology University Hospital Münster Münster NRW Germany; ^4^ Institute of Physiology University of Münster Münster NRW Germany; ^5^ Center for Soft Nanoscience University of Münster Münster NRW Germany

**Keywords:** 8‐hydroxyquinolines, cloxyquin, K_2P_ channels, K_2P_18.1, TRESK

## Abstract

K_2P_18.1 (TRESK) displays one of the most unusual two pore domain potassium channels, that is associated with different neurological and immune‐related diseases emphasizing the therapeutic potential of K_2P_18.1 activators. Previous studies identified cloxyquin and nitroxoline as lead compounds that do not alter the function of other K_2P_ channels. Although their 8‐OH group is crucial for channel activation, 8‐hydroxyquinolines are known for various effects including metal chelation and antibacterial properties increasing the risk of side effects. Therefore, we synthesized 14 quinoline derivatives to search for other tolerated substitutions in 8‐position. Activity determination using two‐electrode voltage clamp (TEVC) revealed that four derivatives achieved moderate inhibitory or stimulatory activity. Among these, benzaldimine **3d** represents a new extended scaffold that unexpectedly increases K_2P_18.1 channel activity by about 60% at 100 µM. Docking studies suggest that despite the enlarged structure, **3d** is still able to achieve in silico interactions with important amino acids from the cloxyquin binding site. However, its usability in vitro is limited by hydrolysis leading to a compound half‐life of 39 min in aqueous solutions. Nevertheless, the identification of a possible scaffold extension for K_2P_18.1 channel activators opens a new opportunity for future compound diversification.

## Graphical abstract

5‐Chloro‐/5‐bromoquinolines reveal how subtle changes at the 8‐position of the quinoline scaffold tune activity at hK_2P_18.1 channels. Among 14 synthesized derivatives, benzaldimine **3d** emerged as a new lead for activator optimization potentiating hK_2P_18.1 activity by around 60% at 100 µM. Docking studies revealed a potential binding mode comparable to cloxyquin binding. However, rapid aqueous hydrolysis requires further structure optimization.

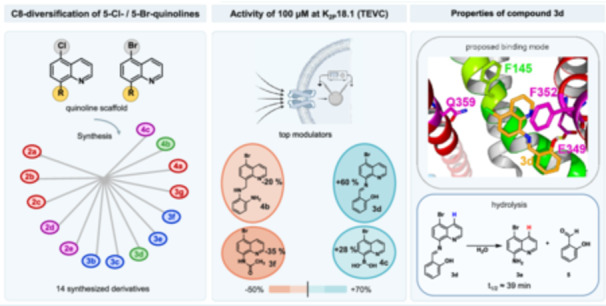

## 1 Introduction

Two‐pore domain potassium (K_2P_) channels constitute a subclass of selective ion channels, that differ in terms of structure and function from voltage‐gated (K_v_), Ca^2+^‐regulated (K_Ca_) or inward rectifying (K_ir_) potassium channels [1]. Unlike the others, K_2P_ channels are formed by dimerization of subunits generating homo‐ but also heterodimeric ion channels [2, 3, 4]. Each monomer possesses four transmembrane helices (M1–M4) and two extracellular helices (E1, E2). Between E2 and M2 as well as M3 and M4, two pore helices (P1, P2) and two pore‐forming loops (SF1, SF2) form a tetramer‐like selectivity filter similar to those of tetrameric potassium channels [5]. In humans, 15 different subunits encoded by *KCNK1*‐*KCNK18* are known, that can be subdivided into six different classes based on their homodimeric ion channel properties [6]. Compared to the other five subclasses consisting of multiple members, the TWIK‐related spinal cord K^+^ (TRESK) channel K_2P_18.1 is the sole member and characterized by several unique features [7]. K_2P_18.1 is the only K_2P_ channel with an additional intracellular domain between M2 and M3, that encompasses approximately 120 amino acids and plays a crucial role in channel activity regulation [8]. This includes the unique mechanism of channel activation by increased intracellular Ca^2+^ levels, that is controlled by direct binding of the Ca^2+^‐dependent phosphatase calcineurin and subsequent ion channel dephosphorylation [9]. Very recently, a similar but not identical mechanism was discovered for the TREK channels K_2P_2.1 and K_2P_10.1 [10]. In comparison, the modulation of the TREK ion channels by Ca^2+^ is related to interactions with their long C‐terminal domain, that is much shorter in K_2P_18.1 [10].

Besides their modulation by Ca^2+^, TREK and TRESK channels also share overlapping expression patterns in neuronal tissues [1, 3, 11]. Especially in neurons from the dorsal root ganglion, K_2P_18.1 and K_2P_10.1 are responsible for the major background K^+^ current subsequently maintaining the resting membrane potential and shaping neuronal excitability [12]. The physiological significance of K_2P_18.1 for neuronal excitability is also underlined by data from *KCNK18* knockout mice as well as stem cell derived neuronal cells [11, 13, 14, 15]. The combination of neuronal expression in certain cell types together with the physiological role to act as a brake for neuronal excitability links dysfunction of K_2P_18.1 with diseases like migraine, neuropathic pain and epilepsy [13, 16, 17, 18, 19]. Moreover, recent studies identified K_2P_18.1 as a crucial switch for regulatory T cell (T_reg_) proliferation and differentiation within the thymus [20]. It was shown pharmacologically that elevated K_2P_18.1 ion channel function can increase the number of immunosuppressive T_reg_ cells in humans, while inhibition resulted in T_reg_ cell number reduction in mice [20]. These results render K_2P_18.1 channel modulation as a future target for drug therapy in autoimmune diseases like multiple sclerosis but also as an add‐on therapy for certain cancer therapies [20, 21].

Since most of the mentioned pathophysiological conditions are associated with reduced channel function, the development of K_2P_18.1 channel activators is of major interest. To date, several lead structures exist that are able to potentiate K_2P_18.1 channel function in an indirect or direct fashion [7, 22, 23]. However, most of them are not very potent or produce major off‐target effects hampering their direct use for efficient drug therapy [7]. Especially the selectivity for K_2P_18.1 over other K_2P_ ion channels display a known issue [23, 24]. Therefore, structural optimization of lead compounds is needed. The structure of cloxyquin (5‐chloro‐8‐hydroxyquinoline) is a starting point for optimization, since it shows moderate potency at K_2P_18.1 but does not alter the function of other K_2P_ channels [22, 25]. Therefore, cloxyquin and its derivative nitroxoline are already used in different studies including previously mentioned T_reg_ study to characterize the physiological relevance of K_2P_18.1 in more detail [21, 26, 27]. The usability of cloxyquin and nitroxoline in patients is limited due to several factors, that are linked to the 8‐OH substitution of the quinoline scaffold. This includes antimicrobial, cytotoxic and metal chelating effects, that are commonly observed for 8‐hydroxyquinolines [28, 29]. On the other hand, the quinoline substructures can be found in many different drug molecules, that are not linked to these specific side effects [29].

In our previous study we characterized the structure‐activity relationships of cloxyquin and identified the mechanism of K_2P_18.1 channel activation [30]. The results showed that a halogen atom in 5‐position (5‐Cl/5‐Br) together with an 8‐OH group synergistically increases the compound efficacy [30]. By interactions with human K_2P_18.1 (hK_2P_18.1) E349 and Q359 through H bond as well as halogen‐π‐interactions, cloxyquin stabilizes the selectivity filter leading to more efficient conduction and therefore channel activation [30]. In this study we tried to find tolerated replacements for the 8‐OH group, that open new options for further K_2P_18.1 channel activator design. We synthesized 14 different cloxyquin analogs, that were tested in two‐electrode voltage clamp (TEVC) recordings for their potential K_2P_18.1 channel modulation. The results support the idea, that a H bond‐donating function in spatial proximity to the 8‐position of the quinoline scaffold is needed for stimulatory activity. Furthermore, the data indicate that rotational freedom and spatial orientation of the tested H bond‐donating substituents might be crucial for activity but also for the mode of action leading to channel inhibition or activation. In total, four out of 15 tested compounds significantly influenced the hK_2P_18.1 channel activity leading to ion channel inhibition (**3f**, **4b**) or activation (**3d**, **4c**).

## 2 Results and Discussion

### 2.1 Chemistry

**Scheme 1:**
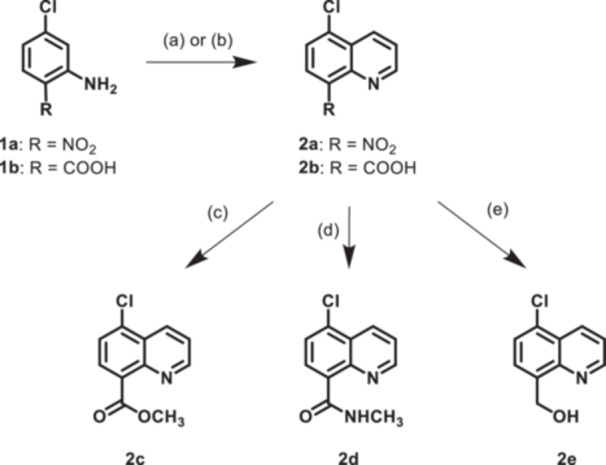
Synthesis of 5‐chloroquinoline derivatives **2b**–**2e**. Reagents and reaction conditions: (a) **1a**, glycerol, H_2_SO_4_ conc., KI, 140°C, 2 h, 16%. (b) **1b**, glycerol, H_2_SO_4_ 80%, KI, 140°C, 6.5 h, 45%. (c) **2b**, H_2_SO_4_ conc., methanol, 80°C, overnight, 33%. (d) **2b**, CH_3_NH_2_, EDC─HCl, NEt_3_, CH_2_Cl_2_, rt, 2 h, 15%. (e) **2b**, 1. oxalyl chloride, DMF, CH_2_Cl_2_, 1 h 0°C, 2 h rt. 2. LiAlH_4_, diethyl ether, −60°C, 1.5 h, 15%.

To enable comparison of suitable modifications at the 8‐position, all synthesized compounds share a quinoline scaffold with a 5‐chloro‐ or 5‐bromo‐substituent, which both were previously identified as crucial for efficient hK_2P_18.1 channel activation [30]. The first test compounds 5‐chloro‐8‐nitroquinoline (**2a**) and 5‐chloroquinoline‐8‐carboxylic acid (**2b**) were synthesized by a Skraup quinoline synthesis heating 5‐chloro‐2‐nitroaniline (**1a**) or 2‐amino‐4‐chlorobenzoic acid (**1b**) in presence of glycerol, KI and conc. H_2_SO_4_ [31, 32]. In comparison to the cited literature, the yields for **2a** (16%) and **2b** (45%) were reduced, which might be related to the electron‐withdrawing effect of the chloro‐substituent reducing the nucleophilic properties of the amines **1a** and **1b** (Scheme 1).

Carboxylic acid **2b** was further used as a starting point for diversification leading to different 5‐chloro‐substituted quinolines **2c**–**2e**. The ester **2c** was obtained in 33% yield by dissolving **2b** in methanol/H_2_SO_4_ conc. and heating the solution under reflux overnight [33, 34]. Amidation of **2b** with methylamine was conducted in the presence of coupling reagent 1‐ethyl‐3‐(3‐dimethylaminopropyl)carbodiimide hydrochloride (EDC‐HCl) to obtain carboxamide **2 d** in 15% yield [35]. For the preparation of primary alcohol **2e**, a two‐step reduction was conducted. Acid **2b** was first reacted with oxalyl chloride to form the corresponding acid chloride and subsequently reduced with LiAlH_4_ to provide **2e** in 15% yield [36, 37].

**Scheme 2:**
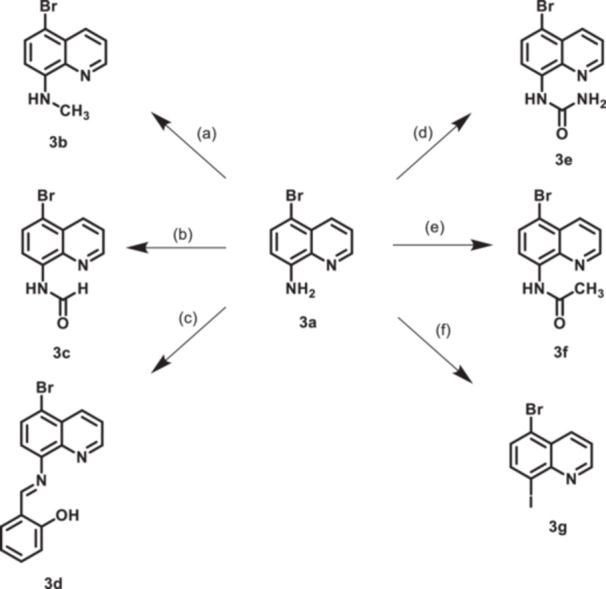
Synthesis of 5‐bromoquinolines **3b**–**3g**. Reagents and reaction conditions: (a) CH_3_I (1 equivalent), K_2_CO_3_, DMF, rt, 2 d, 35%. (b) DMF, KO*t*Bu, rt, 1 d, 6%. (c) salicylaldehyde, EtOH, rt, 10 min, 64%. (d) KOCN, CH_3_COOH/H_2_O (70:30 V/V), rt, 1.5 h, 54%. (e) CH_3_COCl, NEt_3_, CH_2_Cl_2_, rt, 2.5 h, 65%. (f) NaNO_2_, HCl, H_2_O, 0°C, 30 min, then KI, 0°C, 2.5 h, 53%.

As mentioned before, previous structure‐activity relationships indicated comparable hK_2P_18.1 channel activation for 5‐chloro‐ and 5‐bromo‐substitution [30]. Therefore, commercially available 5‐bromoquinolin‐8‐amine (**3a**) served as a building block for the synthesis of 5‐bromo‐substituted quinolines **3b**–**3g** (Scheme 2).

Methylation of the primary amine **3a** with one equivalent of CH_3_I and K_2_CO_3_ in DMF led to the secondary amine **3b** in 35% yield [38]. The formamide **3c** was synthesized via transamidation of *N*,*N*‐dimethylformamide with primary amine **3a** using KO*t*Bu [39, 40]. Unexpectedly, **3c** was obtained in only 6% yield, which is considerably less than previously described [39, 40]. Imine **3d** was obtained in 64% yield by condensation of primary amine **3a** with salicylaldehyde [41]. For synthesis of the urea **3e**, primary amine **3a** was reacted with KOCN and AcOH [42]. After 1.5 h, urea **3e** was isolated in 54% yield. Acetylation of **3a** with acetyl chloride led to the acetamide **3f** in 65% yield. Lastly, a Sandmeyer‐type reaction was conducted to obtain the 8‐iodoquinoline **3g**. For this purpose, **3a** was converted into the corresponding diazonium salt using NaNO_2_/HCl [43]. In a second step, the resulting diazonium salt reacted with KI under temporarily formation of iodine radicals and N_2_ to give 8‐iodoquinoline **3g** in 53% yield [43, 44].

**Scheme 3:**
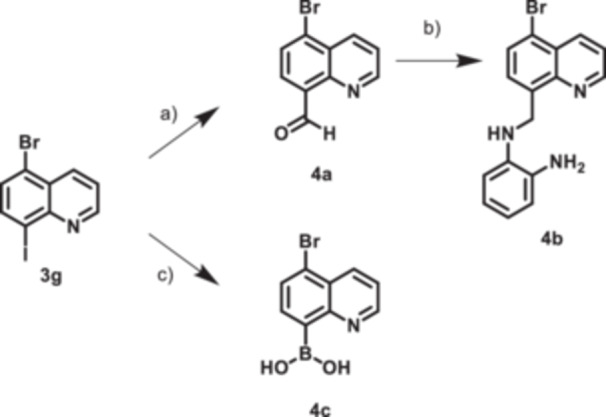
Synthesis of 5‐bromoquinolines **4a**–**4c**. Reagents and reaction conditions: (a) *n*‐BuLi, THF, −78°C, 10 min, then DMF, −78°C, 10 min, 55%. (b) *o*‐phenylenediamine, THF/H_2_O (1:1), reflux, 24 h, 30%. (c) *n*‐BuLi, −78°C, THF, 1 h, then B(OCH_3_)_3_, rt, 1.5 h, 30%.

High yields of **3g** were required to ensure further derivatizations to compounds **4a**–**4c** (Scheme 3).

Aldehyde **4a** was synthesized by a halogen‐metal exchange of **3g** with *n*‐BuLi followed by reaction with DMF in 55% yield [45]. Unexpectedly, conversion of aldehyde **4a** with *o*‐phenylenediamine did not lead to the corresponding benzimidazole as previously described [46]. Instead, analytical data indicates that the reaction resulted in the formation of the diamine **4b**, which was isolated in 30% yield. Finally, another iodine‐lithium exchange at iodoquinoline **3g** with *n*‐BuLi resulted in the aryl‐lithium intermediate, that was further converted with trimethyl borate into the corresponding boronic ester [47]. After hydrolysis with HCl, boronic acid derivative **4c** was isolated in 30% yield.

### 2.2 Pharmacology/Biology

**Figure 1:**
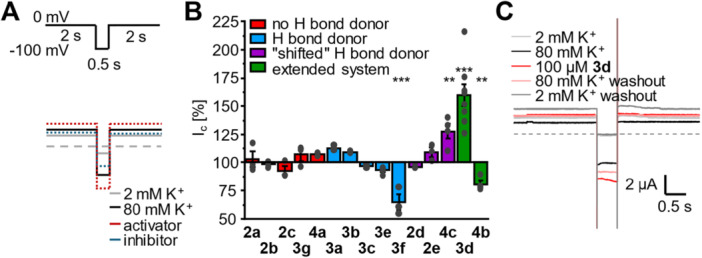
Pharmacological evaluation of 5‐chloro‐/5‐bromo‐quinolines **2**–**4**. (A) Schematic representation of utilized pulse protocol (top) and resulting current traces recorded via TEVC in hK_2P_18.1 channel expressing *Xenopus laevis* oocytes. (B) I_c_ values determined for 100 µM **2**–**4**. Values are given as mean ± SEM. Columns for different compound classes are colored in red (Class 1), blue (Class 2), purple (Class 3), and green (Class 4). Significance of log_2_ (I_c_) ≠ 0 was determined by a general linear regression model (see 4.2–1) and is indicated by **p < 0.01 and ***p < 0.001. (C) Sample trace showing the effect of 100 µM **3d**. SEM, standard error of mean; TEVC, two‐electrode voltage clamp.

In total, 14 synthesized 5‐chloro‐/5‐bromoquinoline derivatives and commercially available primary amine **3a** were evaluated for their modulatory impact at hK_2P_18.1 channel expressed in *Xenopus laevis* oocytes (*n* = 3–8; exact number for each compound is given in Table 1) via TEVC recordings. To ensure comparability with previous data, each compound was tested in the same screening setup as previously described using a single concentration of 100 µM in presence of 80 mM K^+^ buffer and a repetitive pulse protocol (Figure 1A) [20, 25, 30, 48]. While these assay conditions do not fully reflect physiological conditions, they are widely accepted and known for robust K_2P_ channel recordings with low signal‐to‐noise ratios and high reproducibility [20, 25, 30, 48]. Unfortunately, compound concentrations required to elicit effects were so high that further increase would have exceeded the solubility limits, making it impossible to generate proper dose‐response curves.

**Table 1:** Pharmacological data for compounds (cmp.) **2**–**4** evaluated via TEVC recordings.

Cmp.	Mean I_c_ ± SEM [%]	Mean I_w_ ± SEM [%]	*n*	log_2_ (I_c_)					Sig.
				Mean	95% LCI	95% UCI	*t*‐value	*p*‐value	
**2a**	103.2 ± 7.1		3	0.038	−0.193	0.269	0.335	0.739	ns
**2b**	98.3 ± 1.6		3	−0.025	−0.256	0.206	−0.221	0.826	ns
**2c**	93.0 ± 4.2		3	−0.107	−0.338	0.123	−0.940	0.353	ns
**2d**	96.0 ± 0.6		3	−0.059	−0.290	0.172	−0.518	0.607	ns
**2e**	109.2 ± 4.1		3	0.124	−0.107	0.355	1.088	0.283	ns
**3a**	113.2 ± 1.4		3	0.178	−0.053	0.409	1.558	0.127	ns
**3b**	109.5 ± 0.7		3	0.131	−0.100	0.362	1.149	0.258	ns
**3c**	96.6 ± 0.8		3	−0.050	−0.281	0.181	−0.437	0.665	ns
**3d**	159.8 ± 9.8	65.2 ± 6.7	8	0.658	0.466	0.849	6.944	< 0.001	***
**3e**	93.3 ± 1.6		4	−0.101	−0.317	0.115	−0.949	0.348	ns
**3f**	64.6 ± 7.0	68.2 ± 2.1	3	−0.647	−0.878	−0.416	−5.661	< 0.001	***
**3g**	107.0 ± 5.3		3	0.094	−0.137	0.325	0.827	0.413	ns
**4a**	107.0 ± 1.0		3	0.098	−0.133	0.329	0.856	0.397	ns
**4b**	80.7 ± 3.1	65.8 ± 18.8	4	−0.313	−0.529	−0.097	−2.930	0.006	**
**4c**	127.9 ± 6.3	93.2 ± 30.7	4	0.350	0.134	0.566	3.271	0.002	**

*Note:* Change of ion channel current in presence of 100 µM compound (I_c_) as well as effect washout (I_w_) were determined as mean percentage ± SEM from n independent K_2P_18.1 expressing oocytes. For statistical analysis, mean of log_2_‐transformed I_c_ is given together with 95% confidence interval limits (LCI, UCI) as well as determined *t* and *p*‐values for each compound (see materials and methods). Significance (Sig.) of ion channel current modulation (log_2_ (I_c_) ≠ 0) was determined by linear regression model and is indicated by ns for *p* > 0.05.

Abbreviations: SEM, standard error of mean; TEVC, two‐electrode voltage clamp.

** *p* < 0.01;

*** *p* < 0.001.

As demonstrated earlier, quinolines can act as K_2P_18.1 channel inhibitors or activators depending on their substitution pattern, which can complicate the interpretation and comparability of the different effects [48]. Due to biological test variations, it can be difficult to statistically identify moderate levels of activity with certainty even with our optimized protocol. These aspects were considered for activity as well as for statistical analyses. The modulatory effect termed I_c_ in the following (see methods) was quantified by the ratio of currents in presence and absence of test compounds. For statistics, we focused on clear identification of a significant ion channel modulation and not on a comparison between the different compounds. To account for the multiplicative nature of current ratios and to obtain a symmetric scale for better comparability of activation and inhibition, the I_c_ values were log_2_‐transformed. With this transformation, log_2_ (I_c_) = 0 displays no modulatory effect, whereas positive and negative values indicate activation and inhibition, respectively. To determine whether a compound significantly alters activity, estimated means and 95% confidence intervals were obtained from a general linear model and tested for the hypothesis log_2_ (I_c_) ≠ 0 (Table 1). This approach is analogous to established linear‐model analyses of log‐fold changes used in differential omics and chemoproteomic compound profilings [49, 50].

For a more precise data interpretation which kind of functional groups are tolerated as a replacement for the 8‐OH group, compounds were divided into four classes. The first class encompasses compounds with a H bond acceptor functional group in 8‐position, which cannot act as a H bond donor (**2a**, **2b**, **2c**, **3g**, **4a**; Figure 1B compounds with red columns). This also includes carboxylic acid **2b** (p*K*_a_ < 4), that is deprotonated under physiological as well as assay conditions (pH 7.4). The second class displays bioisosteres of phenols and other groups in 8‐position of the quinoline ring, that can donate H bonding (**3a**, **3b**, **3c**, **3e**, **3f**; Figure 1B compounds with blue columns). Within the third class of compounds, the H bond‐donating group is shifted by a carbonyl‐, methylene‐ or boronyl‐spacer (**2e**, **2d, 4c**; Figure 1B compounds with purple columns) to increase the distance to the aromatic core region. The fourth class is characterized by an enlarged ring system in 8‐position with H bond donating properties (**3d**, **4b**; Figure 1B compounds with green columns).

As previously described, the effect of cloxyquin on K_2P_18.1 evaluated by TEVC in *X. laevis* oocytes is slowly reversible showing only 57% of return from stimulated to basal K_2P_18.1 channel current within 15 min [25]. Therefore, this washout effect was quantified for significantly activating or inhibiting compounds.

The screening results showed that tested compounds without H bond donating group (Class 1) were not able to either activate or inhibit the hK_2P_18.1 channel significantly. The resulting I_c_ values for these compounds range from 93% to 107%. These data support the idea of a crucial H bond donation to E349 for hK_2P_18.1 channel activators binding to the cloxyquin binding site [30].

Only one compound (**3f**) of class 2 ligands, which can donate a H bond in 8‐position, was able to alter the hK_2P_18.1 channel activity. The primary and secondary aromatic amines **3a** and **3b**, the formamide **3c** as well as the urea derivative **3e** did not show significant alteration of ion channel activity. The mean I_c_ values for these inactive compounds are between 93% and 113%. Compared to O─H donors, N─H donors tend to form weaker H bonds due to the less electronegative N atom. Especially anilines and amines were described to form only weak H bond interactions [51]. On the other hand, amides and urea derivatives like **3c** and **3e** are in general able to form stronger H bonding indicating that polarization of the N─H bond might be not the only property to influence the strength of H bond interactions [51]. Interestingly, while **3c** and **3e** are inactive, the slightly larger and slightly more hydrophobic acetamide derivative **3f** induces a reversible ion channel inhibition of around 35% (I_c_ 65%) with a washout fraction I_w_ of 65%, that is comparable to the previously published value for cloxyquin [25].

Similar to Class 2, only one compound of Class 3 with a shifted H bond‐donating system was able to alter hK_2P_18.1 channel function. Application of 100 µM carboxamide **2d** (I_c_ 96%) or methanol derivative **2e** (I_c_ 109%) did not significantly alter the hK_2P_18.1 ion channel current, which might be explained by the rotational freedom of the H bond donating substructure resulting in insufficient orientation and/or geometry for strong H bonding with E349. Interestingly, boronic acid derivative **4c** led to a significant increase in ion channel activity with an I_c_ value of 128%. Although the I_w_ of 93% indicates an almost fully reversible effect induced by **4c**, it must be mentioned that this mean value might be distorted by the high variance. The interpretation of the activity of **4c** is complicated by the unique properties of the unusual aryl boronic acid group, which is generally able to form H bond interactions [52]. However, in aqueous solutions aryl boronic acids can react as Lewis acids leading to the sp^3^‐hybridized boronate altering orientation of the H bond donating system [52, 53]. The p*K*_a_ values for aryl boronic acids strongly depend on the ring system as well as on the substitution pattern [52, 53]. While phenylboronic acid has an experimentally determined p*K*_a_ value of 8.88 (8.91 predicted), electron withdrawing groups like halogen atoms in *p*‐position can lower this value tenfold [52, 53]. Additionally, sp^3^‐hybridization of the B atom can also be induced by intramolecular B‐N‐interactions, that have been reported for quinoline‐8‐ylboronic acid [54, 55]. Based on these findings, it is assumed that the major fraction of **4c** is sp^3^‐hybridized under assay conditions.

Most surprisingly, compounds from Class 4 with an extended ring system showed significant inhibitory or stimulatory properties. While the diamine **4b** significantly inhibited the hK_2P_18.1 channel current by around 20% (I_c_ 81%), the benzaldimine **3d** displayed the most active of all tested compounds resulting in an I_c_ value of 160% (Figure 1B,C). For both compounds, washouts of around 65% were observed (I_w_ 65% for benzaldimine **3d**; 66% for 4─*o*‐phenylenediamine **4b**). Although the inhibitory effect of **4b** is weak compared to other channel blockers, the result is in accordance with previous data for a different set of cloxyquin‐derived compounds, where more bulky substitutions at the 8‐position also led to inhibitory effects [48]. On the other hand, these previous findings render the hK_2P_18.1 stimulatory effects of **3d** as even more surprising. Compared to the other classes, **4b** possesses an H bond‐donating group that is in shifted position similar to compounds from class 3, while **3d** does not contain a H bond donating system directly linked to the 8‐position. However, **3d** possesses an OH group in *o*‐position of the additional ring system, that might be able to induce similar interactions like cloxyquin at the hK_2P_18.1 channel binding site. The direct comparison between **3d** and **4b** reveals further structural alterations in terms of geometry and flexibility that might be responsible for the observed activity differences. Therefore, we later performed docking experiments to visualize the potential impact of these differences for **3d** and **4b** at the cloxyquin binding site.

**Figure 2:**
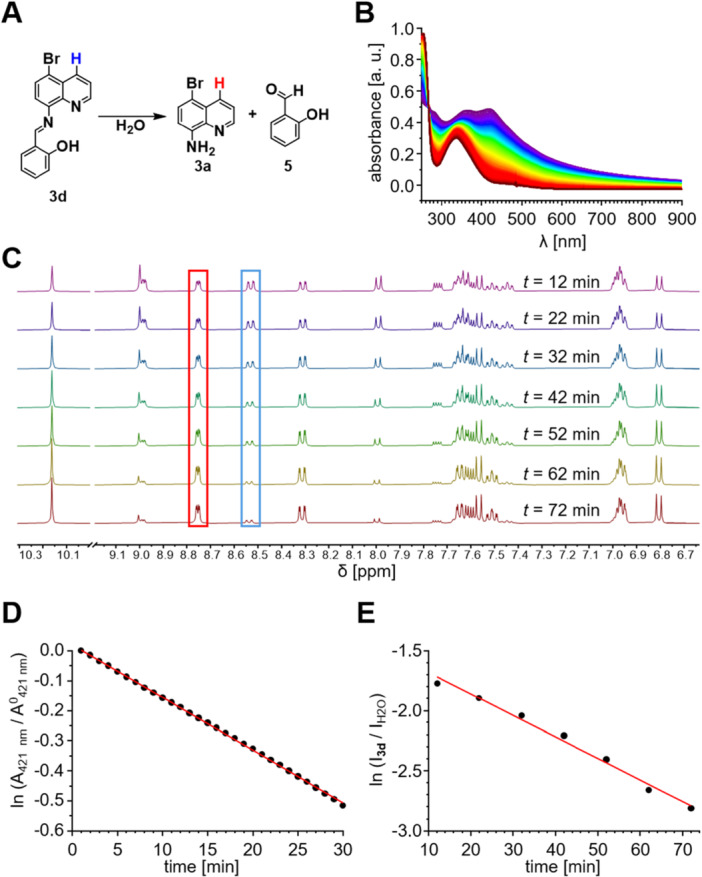
Time‐dependent hydrolysis of **3d**. (A) Reaction scheme of **3d** hydrolysis in aqueous solutions forming **3a** and salicylaldehyde **5**. (B) Overlay of 88 UV‐Vis spectra from 60 s (violet) to 89 min (red) after solution preparation. (C) Overlay of ^1^H‐NMR recorded after 12 min of solution preparation every 10 min. (D, E) Linear regression of UV‐Vis absorbance (ln (A_421 nm_(*t*)/A^0^_421 nm_), 2D) and ^1^H‐NMR data (ln (I_**3d**_/I_H2O_). (E) Fitting procedures and parameters are given in Supporting Information: Table 1 and Section 4.

During the initial TEVC recordings for activity determination, a continuous reduction of activity could be observed for compound **3d**, which is caused by compound decomposition. The imine substructure connecting the two aromatic systems is prone to hydrolyze in aqueous solutions depending on the pH resulting in the formation of **3a** and salicylaldehyde **5** (Figure 2A) [56]. To ensure comparability with the results for the other test compounds, the test solutions for **3d** were freshly prepared for each single oocyte recording and not reused for a second one.

Time‐dependent decomposition of **3d** in aqueous solutions can limit its usability in additional in vitro and in vivo evaluations. Therefore, we quantified the stability and the decay of **3d** over time by UV‐Vis and NMR spectroscopy. For UV‐Vis spectroscopy, **3d** was diluted from a 10 mM stock solution in dry DMSO with DPBS buffer, that is commonly used in cell culture experiments, to a final concentration of 50 µM immediately before the measurement. The resulting aqueous solution was analyzed by spectra recorded every 60 s (rainbow color gradient) for 89 min (Figure 2B). The initial spectrum (violet), recorded approximately 60 s after sample preparation, shows characteristic peaks at approximately 366 nm and approximately 421 nm. Both can be associated with *π* → π* and *n* → π* transitions, that are generally observed for imines [57]. As hydrolysis takes place, the maximum at 366 nm is shifted to 338 nm, while the maximum at 421 nm almost disappears (red spectrum). These changes in the UV spectra correlate with the decrease in size of the conjugated system and thus formation of hydrolysis products **3a** and **5** as well as with the decay of **3d** [57].

As a second method, ^1^H‐NMR spectroscopy was used to observe the decomposition of **3d** in a mixture of DMSO‐d_6_ and D_2_O (82.5%/17.5%) over 72 min (12 min sample preparation, 60 min observation) acquiring spectra every 10 min (Figure 2C). The decay of **3d** as well as the formation of **3a** can be quantified by the comparison of the integrals for the proton in 4‐position of the quinoline scaffold. In case of amine **3a**, the doublet of doublets signal for this proton is downfield shifted from 8.55 ppm (**3d**; Figure 2C, blue box) to 8.75 ppm (Figure 2C, red box) allowing for clear separation and integration of the signals. Due to the large molar excess of water, the concentration of D_2_O/H_2_O remained effectively constant throughout the reaction and was therefore utilized as an internal standard for normalization. By comparing the seven NMR spectra, these signals indicate a time‐dependent decay of **3d** and a simultaneous formation of **3a**.

Since the concentration of H_2_O/D_2_O can be considered constant for both methods due to the large molar excess of water, the half‐life of **3d** in aqueous solutions can be described via pseudo‐first‐order kinetics using the formula ln(c) = ln(c_0_) – k*t (see Section 4). Therefore, to determine the decay time constant k of **3d** via linear regression, the ratio of absorbance at 421 nm (A_421 nm_(*t*)/A^0^_421 nm_) as well as the ratio of NMR signal integral for the previously discussed proton of **3d** compared to water (I_**3d**_/I_H2O_) was transformed using the natural logarithm. In case of UV‐Vis data, a linear decay of ln (A_421 nm_(*t*)/A^0^_421 nm_) can be observed only within the first 30 min (Figure 2D), while for the NMR integral data all seven data points were used to determine k via linear regression (Figure 2E). The determined time constants for both methods are highly similar resulting in k_UV‐Vis_ = 0.01756 min^−1^ and k_NMR_ = 0.01792 min^−1^ (Figure 2D,E and Supporting Information: Table 1). Consequently, the half‐life (t_1/2_) of **3d** calculated by t_1/2_ = ln(2)/k results in approximately 39 min for both methods.

### 2.3 Molecular Modelling

**Figure 3:**
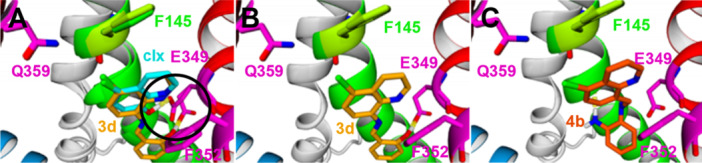
Docking poses for **3d** and **4b** in comparison to cloxyquin (clx). (A) Overlay of newly generated docking pose for **3d** compared to previously generated docking pose of cloxyquin. Most important interacting amino acids (F145, E349, F352, and Q359) are shown from both complexes (hK_2P_18.1/clx, hK_2P_18.1/**3 d**). Major conformational changes of amino acid side chains can only be observed for E349 (black circle). (B, C) Close up depiction of **3d** (B) and **4b** (C) docking poses at the cloxyquin binding site. H bond interactions are indicated by yellow dotted lines.

As previously mentioned, structural differences of **3d** and **4b** result in different geometrical and rotational compound properties. To get an idea of how these differences could alter the binding to the cloxyquin binding site, we performed docking studies using our previously described hK_2P_18.1 model [30]. Based on binding energies, the most stable conformations of **3d** and **4b** were selected for comparison. As indicated by the overlay, **3d** can adopt a similar pose within the binding site like the previously determined pose for cloxyquin (Figure 3A). Interestingly, while most interactions are achievable by **3d** without major conformational changes within the protein, the side chain of E349 needs to rotate by around 65° (C_β_–C_γ_ axis) compared to the cloxyquin pose (Figure 3A, black ring). The rotation can enable the formation of a H bond between the terminal carboxy group of E349 and the compound (Figure 3B). In agreement with the previous cloxyquin pose, aromatic interactions with F145 and halogen–π–interactions with Q359 can be observed for **3d** in silico. In contrast, the postulated pose for **4b** is slightly changed leading to no H bonding with E349 (Figure 3C). While the benzaldimine **3d** forces the orientation of the terminal phenol group into a planar conformation relative to the quinoline scaffold, the terminal ring system of **4b** can be rotated almost independently from the quinoline scaffold. Therefore, the primary amino group of **4b** might be able to interact with other residues near the binding site indicated by the formation of a different H bond with the L148 backbone of the M2 helix. Consequently, **4b** would not have simultaneous interactions with both M4 helices like **3d** or cloxyquin. The additional interaction of **4b** with L148 in combination with F145 interactions might influence the dynamic mobility of the M2 helix in a crucial segment, that was previously described for TREK channels as important for activity adaptation by straightening and buckling of the helix [58, 59]. In summary, differences between **3d** and **4b** can be observed in terms of their possible interactions within the cloxyquin binding pocket in silico. This hypothesis is in accordance with the observed activity data for previously synthesized compounds with bulkier substituents as well as with the previously described activity‐determining interactions with hK_2P_18.1 [30, 48]. However, it must be mentioned that docking of these only moderate active compounds using an in silico model of hK_2P_18.1 bears the risk of misinterpretation. Therefore, our in silico generated hypothesis should be treated as one potential explanation that needs further experimental data including a cryo‐EM or X‐ray structure of hK_2P_18.1 together with one of these compounds.

## 3 Conclusion

Within this study, we synthesized 14 different cloxyquin analogs searching for potential replacements of the 8‐OH group. Out of 15 tested, four compounds showed moderate, but significant inhibitory (**3f**, **4b**) or stimulatory (**3d**, **4c**) effects at hK_2P_18.1 expressing *X. laevis* oocytes at a test concentration of 100 µM. While the number of synthesized derivatives is too small to generate detailed structure–activity relationships, our data is in line with the literature supporting the relevance of a H bond‐donating system near the 8‐position [30]. Compound **3d** was identified as the most active of these newly synthesized compounds with an I_c_ value of 160%. In comparison to previous data, that were all generated under comparable assay conditions, the activity of **3d** ranges between the activity of 100 µM cloxyquin (I_c_ 375%) and 100 µM nitroxoline (I_c_ 115%) [30]. Although the activities of **3d** and nitroxoline are comparably low, it must be mentioned that nitroxoline was able to induce hK_2P_18.1‐dependent effects in human [20]. However, decomposition of **3d** in aqueous solutions resulting in a half‐life of around 39 min emphasizes the need for further structural optimization to generate a compound, that is more useful for future in vitro and potential in vivo evaluation. Still, our findings show that the 8‐OH group is in principle replaceable without abolishing hK_2P_18.1 activity completely. Consequently, the enlarged scaffold of **3d** displays a new template for further development of hK_2P_18.1 activators. Future optimizations should focus on stability improvements. For example, the imine substructure could be substituted with an alkenyl‐group, which would conserve conformational restrictions. To improve **3d** activity in general, systematic variations of the substitution pattern are needed.

## 4 Experimental

### 4.1 Chemistry

#### 4.1.1 General

Chemicals including compounds **1a**, **1b**, and **3a** were purchased from Sigma‐Aldrich or Alfa Aesar. Oxygen and moisture‐sensitive reactions were conducted under dry N_2_ in dried glassware. For thin layer chromatography (TLC), silica gel 60 F254 on aluminum sheets (Merck) was used. Flash chromatography was conducted using silica gel 60 (40–63 µm, Macherey‐Nagel) as stationary phase and compressed air. If applicable, synthetic procedures include column diameter (⊘), length of the stationary phase (*l*), fraction size (*V*) and eluent. Automatic flash chromatography was performed with Isolera™ Spektra One (Biotage®) and SNAP cartridges containing Silica gel 60 (40–63 µm, Macherey‐Nagel) with a fixed fraction size of 20 mL. Wherever applicable, cartridge sizes, flow rate and eluent are given in the synthetic procedures. In the following section, scientific instruments for analytical characterization are given. Melting points: melting point system MP50 (Mettler Toledo), uncorrected. High resolution mass spectrometry (HRMS): MicroTOFQII mass spectrometer (Bruker Daltonics): APCI. ^1^H nuclear magnetic resonance (NMR) (400 MHz/600 MHz), ^13^C NMR (101 MHz/151 MHz): Agilent DD2 400 MHz and 600 MHz spectrometers. Chemical shifts (δ) are reported in parts per million (ppm) against the reference substance tetramethylsilane (TMS) and calculated using the solvent residual peak of the undeuterated solvent. Software for NMR spectra analysis: MestReNova software (version 10.0.0–14381,® 2014 by Mestrelab Research S.L.). Infrared (IR) spectroscopy: FT/IR IRAffinity‐1 IR spectrometer (Shimadzu).

#### 4.1.2 Purity Determination via HPLC

Purity of all synthesized compounds except compound **3d** was determined using high pressure liquid chromatography (HPLC; Merck Hitachi) with the following specifications: pump: L‐7100, degasser: L‐7614, autosampler: L‐7200, UV detector: L‐7400, interface: D‐7000, data transfer: D‐line; column: LiChropher®60 RP‐select B (5 µm), LiChroCART®250‐4 mm cartridge; flow rate 1.0 mL/min; injection volume 5.0 µL; detection wavelength: 210 nm; solvents: demineralized water + 0.05% (V/V) trifluoroacetic acid (A), acetonitrile + 0,05% (V/V) trifluoroacetic acid (B); gradient elution (% A): 0–4 min: 90%; 4–29 min: gradient from 90% to 0%; 29–31 min: 0%; 31–31.5 min: gradient from 0% to 90%; 31.5–40 min: 90%. Data acquisition: HSM‐Software (Merck Hitachi), manual integration. Unless otherwise noted, the determined purity of compounds for pharmacological evaluation is > 95%.

#### 4.1.3 Purity Determination for **3d** via q^1^H‐NMR

Due to strong hydrolytic activity in aqueous solutions, purity of compound **3d** was determined using quantitative ^1^H‐NMR (Agilent DD2 600 MHz spectrometer) recorded with equimolar amounts of the internal calibrant 1,3,5‐trimethoxybenzene. The final purity (*P*) was determined utilizing the mass of internal calibrant (*m*_*ic*_) and sample (*m*_*s*_), the integral of internal calibrant (*Int*_*ic*_) and target analyte (*Int*_*t*_) resonance signal, the number of protons responsible for the corresponding integrals (*n*_*ic*_, *n*_*t*_), the purity of the internal calibrant (*P*_*ic*_) as well as the following equation:

$$P\left[\%\right]=\frac{n_{\mathrm{ic}}\;*\;{\mathrm{Int}}_{t}\;*\;M_{t}\;*\;m_{\mathrm{ic}}}{n_{t}\;*\;{\mathrm{Int}}_{\mathrm{ic}}\;*\;M_{\mathrm{ic}}\;*\;m_{s}}\;*\;P_{\mathrm{IC}}$$

The determined purity for **3d** is given in Section 4.1.11.

#### 4.1.4 Synthesis of 5‐Chloro‐8‐nitroquinoline (**2a**)

5‐Chloro‐2‐nitroaniline (**1a**) (1.01 g, 5.86 mmol, 1.0 eq) and KI (42 mg, 0.25 mmol, 0.04 eq) were mixed with glycerol (1.15 mL, 15.8 mmol, 2.7 eq) and cooled by an ice/water bath. At 0°C, H_2_SO_4_ conc. (0.71 mL, 13.3 mmol, 2.3 mmol) was added which led to a highly viscous red suspension. The mixture was stirred at 140°C for 2 h. After cooling to room temperature, the mixture was diluted with water (30 mL) and extracted three times with CH_2_Cl_2_ (30 mL). The combined organic phases were dried (NaSO_4_) and the solvent was removed in vacuo. The residue was purified by flash chromatography (⊘ = 1 cm, l = 17.5 cm, cyclohexane/ethyl acetate 86:14 140 mL, V = 10 mL), TLC: R_f_ 0.23 (cyclohexane/ethyl acetate 86:14). Yellow solid, mp 139°C, yield 192 mg (16%). Purity (HPLC): 99.8%, t_R_ = 17.71 min. C_9_H_5_ClN_2_O_2_ (Mr = 208.6). HRMS (APCI): *m/z* = 209.0126 (calcd. 209.0112 for C_9_H_6_^35^ClN_2_O_2_^+^ [M + H]^+^). ^1^H NMR (CDCl_3_): δ [ppm] = 7.68 (dd, *J* = 8.7/4.2 Hz, 1H, 3‐H), 7.72 (d, *J* = 8.1 Hz, 1H, 6‐H), 8.00 (d, *J* = 8.1 Hz, 1H, 7‐H), 8.68 (dd, *J* = 8.6/1.6 Hz, 1H, 4‐H), 9.13 (dd, *J* = 4.1/1.7 Hz, 1H, 2‐H). ^13^C NMR (CDCl_3_): δ [ppm] = 123.7 (1 C, C‐3), 123.8 (1 C, C‐6), 125.5 (1 C, C‐7), 127.2 (1 C, C‐4a), 133.4 (1 C, C‐4), 135.8 (1 C, C‐5), 140.3 (1 C, C‐8a), 147.4 (1 C, C‐8), 153.3 (1 C, C‐2). FT‐IR: ṽ [cm^−1^] = 3098 (C‐H_arom_), 1613 (C═C_arom_), 1524 (NO_2_), 1489 (C═C_arom_), 1350 (NO_2_), 783 (C‐Cl).

#### 4.1.5 Synthesis of 5‐Chloroquinoline‐8‐carboxylic Acid (**2b**)

A mixture of 2‐amino‐4‐chlorobenzoic acid (**1b**) (1.31 g, 7.61 mmol, 1.0 eq) and 80% H_2_SO_4_ (2.6 mL) was heated to 140°C. At 140°C KI (10 mg) and glycerol (0.55 mL, 7.49 mmol, 1.0 eq) were added. The violet mixture was stirred at 140°C for 6.5 h turning into a clear brown solution. After cooling to room temperature, the reaction mixture was brought to pH 7 with 1 M NaOH and the pale brown precipitate was filtered. The crude product was recrystallized from ethanol. TLC: R_f_ = 0:18 (cyclohexane/ethyl acetate 67:33). Pale brown solid, mp 209°C, yield 712 mg (45%). C_10_H_6_ClNO_2_ (207.6 g/mol). Purity (HPLC): 97.0%, t_R_ = 14.51 min. HRMS (APCI): *m/z* = 208.0152 (calcd. 208.0160 for C_10_H_7_^35^ClNO_2_^+^ [M + H]^+^). ^1^H NMR (600 MHz, CDCl_3_): δ [ppm] = 7.74 (dd, *J* = 8.6/4.4 Hz, 1H, 3‐H), 7.85 (d, *J* = 8.0 Hz, 1H, 6‐H), 8.74 (d, *J* = 8.0 Hz, 1H, 7‐H), 8.84 (dd, *J* = 8.6/1.7 Hz, 1H, 4‐H), 9.00 (dd, *J* = 4.4/1.7 Hz, 1H, 2‐H). A signal for the CO_2_H proton is not seen in the ^1^H NMR spectrum. ^13^C NMR (151 MHz, CDCl_3_): δ [ppm] = 122.6 (1 C, C‐3), 123.9 (1 C, C‐5), 126.6 (1 C, C‐4a), 127.8 (1 C, C‐6), 135.4 (1 C, C‐7), 136.0 (1 C, C‐4), 137.1 (1 C, C‐8), 145.9 (1 C, C‐8a), 149.3 (1 C, C‐2), 166.5 (1 C, COOH). FT‐IR: ṽ [cm^−1^] = 3098 (═C‐H_arom_), 1713 (C═O), 1574, 1493, 1458, (C═C_arom_), 799 (C‐Cl).

#### 4.1.6 Synthesis of Methyl 5‐chloroquinoline‐8‐carboxylate (**2c**)

Carboxylic acid **2b** (33.9 mg, 0,16 mmol) was suspended in methanol (2 mL). H_2_SO_4_ conc. (0.1 mL) was added and the brown solution was stirred overnight at room temperature. CH_2_Cl_2_ (2 mL) was added and the mixture was neutralized with saturated aqueous NaHCO_3_ solution. The mixture was extracted three times with CH_2_Cl_2_ (5 mL), the combined organic layers were washed with water (5 mL) and brine (5 mL), dried (Na_2_SO_4_) and the solvent was removed *in vacuo*. The crude product was purified by flash chromatography (⊘ = 1 cm, l = 12:5 cm, cyclohexane/ethyl acetate 67:33 120 mL, V = 5 mL). TLC: R_f_ = 0.26 (cyclohexane/ethyl acetate 67:33). Colorless solid, mp 52°C, yield 12 mg (33%). C_11_H_8_ClNO_2_ (221.6 g/mol). Purity (HPLC): 99.2%, t_R_ = 12.15 min. HRMS (APCI): *m/z* = 222.0297 (calcd. 222.0316 for C_11_H_9_^35^ClNO_2_^+^ [M + H]^+^). ^1^H NMR (600 MHz, CDCl_3_): δ [ppm] = 4.05 (s, 3H, CH_3_), 7.57 (dd, *J* = 8.6/4.2 Hz, 1H,3‐H), 7.66 (d, *J* = 7.8 Hz, 1H, 6‐H), 7.96 (d, *J* = 7.8 Hz, 1H, 7‐H), 8.63 (dd, *J* = 8.6/1.7 Hz,1H, 4‐H), 9.10 (dd, *J* = 4.2/1.7 Hz, 1H, 2‐H). ^13^C NMR (151 MHz, CDCl_3_): δ [ppm] = 52.9 (1 C, CH_3_), 122.5 (1 C, C‐3), 126.0 (1 C, C‐6), 126.7 (1 C, C‐4a), 130.3 (1 C, C‐7), 131.0 (1 C, C‐5), 133.2 (1 C, C‐4), 135.0 (1 C, C‐8), 146.3 (1 C, C‐8a), 152.1 (1 C, C‐2), 167.7 (1 C, C═O). FT‐IR: ṽ [cm^−1^] = 3013 (C‐H_arom_), 2959 (C‐H_aliph_), 1744 (C═O), 1562, 1493, 1443 (C═C_arom_), 787 (C‐Cl).

#### 4.1.7 Synthesis of Methyl 5‐Chloro‐*N*‐methylquinoline‐8‐carboxamide (**2d**)

Under dried N_2_, methylamine (2 M in THF, 0.48 mL, 1.0 eq), EDC‐HCl (290 mg, 1.51 mmol, 1,6 eq) and triethylamine (0.18 mL, 1.30 mmol, 1.4 eq) were dissolved in dry CH_2_Cl_2_ (20 mL). The mixture was cooled to 0°C and carboxylic acid **2b** (195 mg, 0.94 mmol, 1.0 eq) was added. The mixture was stirred for 2 h at room temperature. Saturated aqueous NH_4_Cl solution was added and the aqueous phase was extracted three times with ethyl acetate (20 mL). The combined organic layers were washed with brine (20 mL), dried (Na_2_SO_4_) and the solvent was removed *in vacuo*. The crude product was purified by flash chromatography (⊘ = 2 cm, l = 13 cm, cyclohexane/ethyl acetate 67:33 120 mL, V = 5 mL). The product was recrystallized from ethanol. TLC: R_f_ = 0:34 (cyclohexane/ethyl acetate 67:33). Purity (HPLC): 97.8%, t_R_ = 14.43 min. HRMS (APCI): *m/z* = 221.0476 (calcd. 221.0476 for C_11_H_10_^35^ClN_2_O^+^ [M + H]^+^). ^1^H NMR (600 MHz, CDCl_3_): δ [ppm] = 3.14 (d, *J* = 4.8 Hz, 3H, CH_3_), 7.74 (dd, *J* = 8.6/4.4 Hz, 1H, 3‐H), 7.85 (d, *J* = 8.0 Hz, 1H, 6‐H), 8.75 (d, *J* = 7.9 Hz, 1H, 7‐H), 8.84 (dd, *J* = 8.7/1.7 Hz, 1H, 4‐H), 9.00 (dd, *J* = 4.4/1.7 Hz, 1H, 2‐H). A signal for the NH proton is not seen in the 1H NMR spectrum. ^13^C NMR (151 MHz, CDCl_3_): δ [ppm] = 21.2 (1 C, CH_3_), 122.1 (1 C, C‐3), 123.9 (1 C, C‐5), 126.6 (1 C, C‐4a), 127.8 (1 C, C‐6), 135.4 (1 C, C‐7), 136.0 (1 C, C‐4), 137.1 (1 C, C‐8) 146.0 (1 C, C‐8a), 149.3 (1 C, C‐2), 166.4 (1 C, C═O). FT‐IR: ṽ [cm^−1^] = 1717 (C═O), 1663 (N‐H_bend_), 1574, 1493 (C═C_arom_), 799 (C─Cl).

#### 4.1.8 Synthesis of (5‐Chloroquinolin‐8‐yl)methanol (**2e**)

Under dried N_2_, carboxylic acid **2b** (200 mg, 0.96 mmol, 1.0 eq) was suspended in dry CH_2_Cl_2_ (5.2 mL). Oxalyl chloride (0.14 mL, 1.63 mmol, 1.7 eq) was added dropwise. After addition of DMF (3 drops) leading to a bubbling brown solution, the reaction mixture was stirred for 1 h at 0°C and 2 h at room temperature leading to a light brown suspension. The solvent was removed *in vacuo* and the orange residue was added to a suspension of LiAlH_4_ at −60°C, MgSO_4_ heptahydrate and H_2_O (2 mL) were added leading to a brown suspension A (52.6 mg, 1.39 mmol, 3.0 eq) in dry diethyl ether (1.3 mL) at −60°C. After stirring for 1.5 h a mixture of diethyl ether and aqueous NaHCO_3_ (10%, 1:1, 20 mL) was added. After filtration, the aqueous phase was extracted two times with diethyl ether (10 mL), the combined organic layers were dried (NaSO_4_) and the solvent was removed in vacuo. The crude product was purified by flash chromatography (⊘ = 1 cm, l = 13 cm, cyclohexane/ethyl acetate 67:33 90 mL, V = 5 mL). TLC: R_f_ = 0:21 (cyclohexane/ethyl acetate 67:33). Pale yellow solid, mp 130°C, yield 28 mg (15%). C_10_H_8_ClNO (193.6 g/mol). Purity (HPLC): 96.3%, t_R_ = 12.57 min. HRMS (APCI): *m/z* = 194.0322 (calcd. 194.0367 for C_10_H_9_^35^ClNO^+^ [M + H]^+^). 1H NMR (600 MHz, CDCl_3_): δ [ppm] = 4.60–4.81 (broad, 1H, OH), 5.17 (s, 2H, CH_2_), 7.53 (d, *J* = 7.6 Hz, 1H, 7‐H), 7.56 (dd, *J* = 8.5/4.2 Hz, 1H, 3‐H), 7.59 (d, *J* = 7.6 Hz, 1H, 6‐H) 8.63 (dd, *J* = 8.5/1.7 Hz, 1H, 4‐H), 8.93 (dd, *J* = 4.2/1.7 Hz, 1H, 2‐H). ^13^C NMR (151 MHz, CDCl_3_): δ [ppm] = 64.4 (1 C, CH_2_), 122.1 (1 C, C‐3), 126.5 (1 C, C‐6), 126.6 (1 C, C‐4a), 127.8 (1 C, C‐7), 131.0 (1 C, C‐5), 133.9 (1 C, C‐4), 137.7 (1 C, C‐8), 147.8 (1 C, C‐8a), 149.8 (1 C, C‐2). FT‐IR: ṽ [cm^−1^] = 3275 (O‐H), 1578, 1501, 1454 (C═C_arom_), 1454, 1385 (C‐H_aliph bend_), 775 (C─Cl).

#### 4.1.9 Synthesis of 5‐Bromo‐*N*‐methylquinoline‐8‐amine (**3b**)

5‐Bromoquinoline‐8‐amine (**3a**) (509 mg, 2.28 mmol, 1.0 eq) and CH_3_I (0.18 mL, 2.9 mmol, 1.3 eq) were dissolved in DMF (10 mL). After addition of K_2_CO_3_ (323 mg, 2.34 mmol, 1.0 eq), the mixture was stirred for 2 d at room temperature. The yellow reaction mixture was then diluted with water (20 mL) and extracted three times with ethyl acetate (20 mL). The combined organic layers were washed three times with brine (10 mL), dried (Na_2_SO_4_) and the solvent was removed in vacuo. The crude product was purified by flash chromatography (⊘ = 3 cm, l = 13:5 cm, cyclohexane/ethyl acetate 86:14 420 mL, V = 10 mL). TLC: R_f_ = 0:46 (cyclohexane/ethyl acetate 86:14). Yellow solid, light unstable, mp 76°C, yield 190 mg (35%). C_10_H_9_BrN_2_ (237.1 g/mol). Purity (HPLC): 99.8%, t_R_ = 20.15 min. HRMS (APCI): *m/z* = 237.0003 (calcd. 237.0022 for C_10_H_10_^79^BrN^+^ [M + H]^+^). ^1^H NMR (600 MHz, CDCl_3_): δ [ppm] = 3.02 (d, *J* = 4.9 Hz, 3H, CH_3_), 6.21 (d, *J* = 4.9 Hz, 1H, NH), 6.50 (d, *J* = 8.3 Hz, 1H, 7‐H), 7.47 (dd, J = 8.5/4.2 Hz, 1H, 3‐H), 7.63 (d, *J* = 8.2 Hz, 1H, 6‐H), 8.42 (dd, *J* = 8.5/1.6 Hz, 1H, 4‐H), 8.71 (dd, *J* = 4.2/1.6 Hz, 1H, 2‐H). ^13^C NMR (151 MHz, CDCl_3_): δ [ppm] = 30.1 (1 C, CH_3_), 104.6 (1 C, C‐7), 105.4 (1 C, C‐5), 122.6 (1 C, C‐3), 127.6 (1 C, C‐8a), 131.4 (1 C, C‐6), 135.6 (1 C, C‐4), 139.0 (1 C, C‐4a), 145.9 (1 C, C‐8), 147.3 (1 C, C‐2). FT‐IR: ṽ [cm^−1^] = 3414 (N─H), 3075 (C–H_arom_), 2905 (C–H_aliph_), 1516, 1470, 1427 (C═C_arom_), 783 (C‐Br).

#### 4.1.10 Synthesis of *N*‐(5‐bromoquinolin‐8‐yl)formamide (**3c**)

Under dry N_2_, **3a** (226 mg, 1.01 mmol, 1.0 eq) was dissolved in DMF (0.5 mL, 6.0 mmol, 6.5 eq). KOtBu (303 mg, 2.70 mmol, 2.7 eq) was added and the mixture was stirred over night at room temperature before water (2 mL) was added forming a brown precipitate. Ethyl acetate (10 mL) was added and the two phases were separated. The organic layer was washed two times with brine (10 mL), dried (Na_2_SO_4_) and the solvent was removed *in vacuo*. The residue was purified by flash chromatography (⊘ = 2.5 cm, l = 14 cm, cyclohexane/ethyl acetate 80:20 200 mL, V = 10 mL). TLC: R_f_ = 0.19 (cyclohexane/ethyl acetate 80:20). Orange solid, light unstable, mp 216°C, yield 15 mg (6%). C_10_H_7_BrN_2_O (251.1 g/mol). Purity (HPLC): 97.6%, t_R_ = 28.63 min. HRMS (APCI): *m/z* = 250.9797 (calcd. 250.9815 for C_10_H_8_^79^BrN_2_O^+^ [M + H]^+^). 1H NMR (600 MHz, CDCl_3_): δ [ppm] = 7.59 (dd, *J* = 8.5/4.2 Hz, 1H, 3‐H), 7.81 (d, *J* = 8.4 Hz, 1H, 6‐H), 8.55 (dd, *J* = 8.5/1.6 Hz, 1H, 4‐H), 8.65 (d, *J* = 8.3 Hz, 1H, 7‐H), 8.70 (d, *J* = 1.7 Hz, 1H, O═CH), 8.84 (dd, *J* = 4.2/1.6 Hz, 1H, 2‐H), 9.78 (broad, *J* = 1.7 Hz, 1H, NH). ^13^C NMR (151 MHz, CDCl_3_): δ [ppm] = 115.2 (1 C, C‐5), 118.2 (1 C, C‐7), 123.0 (1 C, C‐3), 127.4 (1 C, C‐4a), 131.0 (1 C, C‐6), 133.6 (1 C, C‐8), 136.2 (1 C, C‐4), 139.2 (1 C, C‐8a), 149.1 (1 C, C‐2), 159.3 (1 C, C═O). FT‐IR: ṽ [cm^−1^] = 3252 (N‐H), 2963 (C‐H_arom_), 2851 (H‐C═O), 1859 (C═O), 1528, 1470, 1450 (C═C_arom_), 783 (C‐Br).

#### 4.1.11 Synthesis of *N*‐(5‐bromoquinolin‐8‐yl)2─hydroxybenzaldimine (**3d**)

**3a** (234 mg, 1.05 mmol, 1.0 eq) was suspended in ethanol (5 mL). Salicylaldehyde (0.11 mL, 1.1 mmol, 1.0 eq) was added turning the mixture into an orange solution. The mixture was stirred at room temperature for 10 min forming an orange precipitate. The solvent was removed in vacuo and the residue was recrystallized from ethanol. TLC: R_f_ = 0.1 (cyclohexane/ethyl acetate 80:20). Orange solid, light instable, mp 119°C, yield 220 mg (64%). Purity (q1H‐NMR (600 MHz, CDCl_3_)): 99.8%. C_16_H_11_BrN_2_O (327.2 g/mol). HRMS (APCI): *m/z* = 327.0098 (calcd. 327.0128 for C_16_H_12_^79^BrN_2_O^+^ [M + H]^+^). ^1^H NMR (600 MHz, CDCl_3_): δ [ppm] = 6.93–6.97 (m, 1H, 4‐H_phenol_), 7.08 (ddd, *J* = 8.3/1.2/0.6 Hz, 1H, 3‐H_phenol_), 7.38 (d, *J* = 8.0 Hz, 1H, 7‐H), 7.39–7.43 (m, 1H, 5‐H_phenol_), 7.45 (dd, *J* = 7.7/1.7 Hz, 1H, 6‐H_phenol_), 7.55–7.59 (m, 1H, 3‐H), 7.86 (d, *J* = 7.9 Hz, 1H, 6‐H), 8.57 (dd, *J* = 8.5/1.7 Hz, 1H, 4‐H), 8.90 (s, 1H, H‐C═N), 9.00 (dd, *J* = 4.1/1.7 Hz, 1H, 2‐H), 13.71 (s, 1H, OH). ^13^C NMR (151 MHz, CDCl_3_): δ [ppm] = 117.8 (1 C, C‐3_phenol_), 119.1 (1 C, C‐4_phenol_), 119.2 (1 C, C‐7), 119.4 (1 C, C‐5), 119.6 (1 C, C‐2_phenol_), 123.0 (1 C, C‐3), 128.5 (1 C, C‐4a), 130.4 (1 C, C‐6), 132.6 (1 C, C‐6_phenol_), 133.7 (1 C, C‐5_phenol_), 135.7 (1 C, C‐4), 143.1 (1 C, C‐8a), 145.7 (1 C, C‐8), 151.1 (1 C, C‐2), 162.1 (1 C, C‐1_phenol_), 165.1 (1 C, C═N). FT‐IR: ‌ṽ [cm^−1^] = 1613 (C═N), 1574, 1485, 1458 (C═C_arom_), 748 (C‐Br). A signal for the O−H bond stretching is not seen in the IR spectrum.

#### 4.1.12 Synthesis of 1─(5‐Bromoquinolin‐8‐yl)urea (**3e**)

**3a** (224 mg, 1.00 mmol, 1.0 eq) was dissolved in acetic acid (7 mL). The orange solution was diluted with water (3 mL) and a 50°C warm solution of KOCN (106 mg, 1.31 mmol, 1.3 eq) in water (5 mL) was added under vigorous stirring. The mixture was kept for 2.5 h at room temperature forming a yellow precipitate. The precipitate was filtered, washed with water and recrystallized from ethanol. TLC: R_f_ = 0.11 (cyclohexane/ethyl acetate 67:33). Beige solid, mp 240°C (decomposition), yield 145 mg (54%). C_10_H_8_BrN_3_O (266.1 g/mol). Purity (HPLC): 98.9%, t_R_ = 16.74 min. HRMS (APCI): *m/z* = 265.9896 (calcd. 265.9924 for C_10_H_9_^79^BrN_3_O^+^ [M + H]^+^). 1H NMR (400 MHz, DMSO‐d_6_): δ [ppm] = 6.65 (s, 2H, NH_2_), 7.76 (dd, *J* = 8.5/4.2 Hz, 1H, 3‐H), 7.84 (d, *J* = 8.5 Hz, 1H, 6‐H), 8.45–8.51 (m, 2H, 4‐H, 7‐H), 8.94 (dd, *J* = 4.2/1.6 Hz, 1H, 2‐H), 9.44 (s, 1H, NH). ^13^C NMR (101 MHz, DMSO‐d_6_): δ [ppm] = 110.2 (1 C, C‐5), 114.7 (1 C, C‐7), 123.3 (1 C, C‐3), 126.5 (1 C, C‐4a), 130.9 (1 C, C‐6), 135.2 (1 C, C‐4), 137.0 (1 C, C‐8a), 138.4 (1 C, C‐8), 148.8 (1 C, C‐2), 155.7 (1 C, C═O). FT‐IR: ṽ [cm^−1^] = 3375 (N‐H), 3179 (C–H_arom_), 1713 (C═O), 1674, 1639 (C═C_arom_), 1480 (C═C_arom_), 783 (C‐Br).

#### 4.1.13 Synthesis of *N*‐(5‐bromoquinolin‐8‐yl)acetamide (**3f**)

**3a** (239 mg, 1.07 mmol, 1.0 eq) and NEt_3_ (0.14 mL, 1.0 mmol, 0.9 eq) were dissolved in CH_2_Cl_2_ (4 mL). Acetyl chloride (0.08 mL, 1.1 mmol, 1.1 eq) in CH_2_Cl_2_ (1 mL) was added dropwise and the mixture stirred for 2.5 h at room temperature. The organic layer was washed with saturated NaHCO_3_ solution (7.5 mL) and the aqueous layer was extracted with CH_2_Cl_2_ (7.5 mL). The residue was purified by flash chromatography (⊘ = 2 cm, l = 15 cm, cyclohexane/ethyl acetate 67:33 210 mL, V = 10 mL). TLC: R_f_ = 0.18 (cyclohexane/ethyl acetate 67:33). Colorless solid, mp 145°C, yield 186 mg (65%). C_11_H_9_BrN_2_O (265.1 g/mol). Purity (HPLC): 99.8%, t_R_ = 18.97 min. HRMS (APCI): *m/z* = 264.9968 (calcd. 264.9971 for C_11_H_10_^79^BrN_2_O^+^ [M + H]^+^). ^1^H NMR (600 MHz, CDCl_3_): δ [ppm] = 2.35 (s, 3H, CH_3_), 7.57 (dd, *J* = 8.5/4.2 Hz, 1H, 3‐H), 7.79 (d, *J* = 8.4 Hz, 1H, 6‐H), 8.53 (dd, *J* = 8.5/1.6 Hz, 1H, 4‐H), 8.66 (d, *J* = 8.4 Hz, 1H, 7‐H), 8.82 (dd, *J* = 4.2/1.7 Hz, 1H, 2‐H), 9.76 (s, 1H, NH). ^13^C NMR (151 MHz, CDCl_3_): δ [ppm] = 25.3 (1 C, CH_3_), 114.3 (1 C, C‐5), 117.1 (1 C, C‐7), 122.8 (1 C, C‐3), 127.3 (1 C, C‐4a), 131.1 (1 C, C‐6), 134.6 (1 C, C‐8), 136.2 (1 C, C‐4), 139.1 (1 C, C‐8a), 148.8 (1 C, C‐2), 168.9 (1 C, C═O). FT‐IR: ṽ [cm^−1^] = 3348 (N─H), 1678 (C═O), 1589, 1470, 1451 (C═C_arom_), 783 (C‐Br).

#### 4.1.14 Synthesis of 5‐Bromo‐8‐iodoquinoline (**3g**)

**3a** (1.78 g, 8 mmol, 1.0 eq) was suspended in water (15 mL) and HCl conc. (3 mL). After addition of NaNO_2_ (833 mg, 12 mmol, 1.5 eq) in water (3 mL), the mixture was stirred for 30 min at 0°C turning into a green suspension. A cooled solution of KI (3.34 g, 20 mmol, 2.5 eq) was added and the resulting brown mixture was stirred for 2.5 h at room temperature. The mixture was extracted three times with ethyl acetate (30 mL). The combined organic phases were washed with aqueous Na_2_S_2_O_3_ solution, dried (NaSO_4_) and filtered over celite. The filtrate was concentrated *in vacuo* and the residue was purified by flash chromatography (⊘ = 6 cm, l = 13 cm, cyclohexane/ethyl acetate 86:14 1050 mL, cyclohexane/ethyl acetate 75:25 400 mL, V = 20 mL). TLC: R_f_ = 0.58 (cyclohexane/ethyl acetate 86:14). Pale yellow solid, mp 129°C, yield 1.4 g (53%). C_9_H_5_BrIN (334.0 g/mol). Purity (HPLC): 98.2% (t_R_ = 21.08 min). HRMS (APCI): *m/z* = 333.8692 (calcd. 333.8723 for C_9_H_6_^79^BrIN^+^ [M + H]^+^). ^1^H NMR (600 MHz, CDCl_3_): δ [ppm] = 7.55 (dd, *J* = 8.5/4.2 Hz, 1H, 3‐H), 7.58 (d, *J* = 7.9 Hz, 1H, 6‐H), 8.21 (d, *J* = 7.9 Hz, 1H, 7‐H), 8.52 (dd, *J* = 8.5/1.6 Hz, 1H, 4‐H), 9.02 (dd, *J* = 4.2/1.6 Hz, 1H, 2‐H). ^13^C NMR (151 MHz, CDCl_3_): δ [ppm] = 103.3 (1 C, C‐5), 123.2 (1 C, C‐4a), 123.3 (1 C, C‐3), 128.3 (1 C, C‐8), 131.5 (1 C, C‐6), 136.5 (1 C, C‐4), 140.0 (1 C, C‐7), 147.8 (1 C, C‐8a), 152.2 (1 C, C‐2). FT‐IR: ṽ [cm^−1^] = 3067 (C‐H_aliph_), 1539, 1474, 1446 (C═C_arom_), 818 (C‐I), 771 (C‐Br).

#### 4.1.15 Synthesis of 5‐bromoquinoline‐8‐carbaldehyde (**4a**)

Under dry N_2_, **3 g** (401 mg, 1.20 mmol, 1.0 eq) was dissolved in dry THF (4 mL). *n*‐BuLi (1.6 M in n‐hexane, 1.2 mL, 1.9 mmol, 1.6 eq) was added dropwise at −78°C leading to a brown solution. After dropwise addition of dry DMF (0.45 mL, 5.9 mmol, 4.9 eq), a precipitate formed and the mixture was stirred for 10 min at −78°C. Water (5 mL) was added and the mixture was poured into a saturated aqueous solution of NaHCO_3_ (25 mL). The aqueous phase was extracted three times with ethyl acetate (20 mL), the combined organic layers were dried (Na_2_SO_4_) and the solvent was removed in vacuo. The crude product was purified by flash chromatography (⊘ = 2 cm, l = 16 cm, cyclohexane/ethyl acetate 90:10 200 mL, V = 10 mL). TLC: R_f_ = 0.30 (cyclohexane/ethyl acetate 90:10). Pale yellow solid, mp 131°C, yield 155 mg (55%). C_10_H_6_BrNO (236.1 g/mol). Purity (HPLC): 97.7%, t_R_ = 16.01 min. HRMS (APCI): *m/z* = 235.9676 (calcd. 235.9706 for C_10_H_7_^79^BrNO^+^ [M + H]^+^). ^1^H NMR (600 MHz, CDCl_3_): δ [ppm] = 7.62 (dd, *J* = 8.6/4.2 Hz, 1H, 3‐H), 7.99 (dd, *J* = 7.7/0.8 Hz, 1H, 7‐H), 8.17 (d, *J* = 7.9 Hz, 1H, 6‐H), 8.64 (dd, *J* = 8.6/1.7 Hz, 1H, 4‐H), 9.07 (dd, *J* = 4.2/1.7 Hz, 1H, 2‐H), 11.42 (d, *J* = 0.8 Hz, 1H, O═C‐H). ^13^C NMR (151 MHz, CDCl_3_): δ [ppm] = 123.0 (1 C, C‐3), 127.9 (1 C, C‐4a), 129.3 (1 C, C‐5), 129.5 (1 C, C‐6), 130.5 (1 C, C‐7), 131.5 (1 C, C‐8), 136.0 (1 C, C‐4), 148.1 (1 C, C‐8a), 152.0 (1 C, C‐2), 192.3 (1 C, HC═O). FT‐IR: ṽ [cm^−1^] = 3075 (C–H_arom_), 2862 (C–H_aliph_), 1674 (C═O), 1559, 1593, 1493 (C═C_arom_), 768 (C‐Br).

#### 4.1.16 Synthesis of *N*‐[(5‐bromoquinolin‐8‐yl)methyl]benzene‐1,2─diamine (**4b**)

**4a** (93 mg, 0.40 mmol, 1.0 eq) and o‐phenylenediamine (126 mg, 1.16 mmol, 2.9 eq) were dissolved in THF/water (1:1, 2 mL) and the solution was heated to reflux for 24 h. After cooling to room temperature, the mixture was diluted with ethyl acetate (10 mL). The organic phase was separated, washed with brine (10 mL), dried (Na_2_SO_4_) and the solvent was removed *in vacuo*. The crude product was purified by flash chromatography (⊘ = 1 cm, l = 18 cm, cyclohexane/ethyl acetate 75:25 100 mL, V = 5 mL). TLC: R_f_ = 0.13 (cyclohexane/ethyl acetate 75:25). Pale yellow solid, mp 173°C, yield 35 mg (30%). C_16_H_14_BrN_3_ (328.2 g/mol). Purity (HPLC): 98.3%, t_R_ = 16.14 min. HRMS (APCI): *m/z* = 328.0471 (calcd. 328.0444 for C_16_H_15_^79^BrN^+^ [M + H]^+^). ^1^H NMR (600 MHz, CDCl_3_): δ [ppm] = 4.93 (s, 2H, CH_2_), 6.68 ‐ 6.71 (m, 2H, 3‐H_aniline_, 6‐H_aniline_), 6.72 ‐ 6.75 (m, 2H, 4‐H_aniline_, 5‐H_aniline_), 7.54 (dd, *J* = 8.5/4.1 Hz, 1H, 3‐H), 7.58 (d, *J* = 7.7 Hz, 1H, 7‐H), 7.76 (d, *J* = 7.6 Hz, 1H, 6‐H), 8.57 (dd, *J* = 8.5/1.7 Hz, 1H, 4‐H), 8.96 (dd, *J* = 4.2/1.7 Hz, 1H, 2‐H). Signals for the NH_2_ and NH protons are not seen in the ^1^H NMR spectrum. ^13^C NMR (151 MHz, CDCl_3_): δ [ppm] = 45.6 (1 C, CH_2_), 113.3 (1 C, C‐3_aniline_), 116.6 (1 C, C‐6_aniline_), 119.3 (1 C, C‐4_aniline_), 120.6 (1 C, C‐5_aniline_), 121.0 (1 C, C‐4a), 122.4 (1 C, C‐3), 127.8 (1 C, C‐5), 128.9 (1 C, C‐7), 130.3 (1 C, C‐6), 135.1 (1 C, C‐2_aniline_), 136.1 (1 C, C‐4), 137.5 (1 C, C‐1_aniline_), 137.7 (1 C, C‐8a), 147.5 (1 C, C‐8), 150.2 (1 C, C‐2). FT‐IR: ṽ [cm^−1^] = 3395, 3345, 3229 (N–H), 3040(C‐H_arom_), 2963, 2897 (C–H_aliph_), 1647, 1597, 1570 (C═C_arom_), 1261 (C–N), 733 (C–Br).

#### 4.1.17 Synthesis of 5‐bromoquinoline‐8‐boronic acid (**4c**)

Under dry N_2_, **3g** (1.00 g, 3.00 mmol, 1.0 eq) was dissolved in dry THF (10 mL) and the solution was cooled to −78°C. *n*‐BuLi (1.6 M in n‐hexane, 2.3 mL, 3.7 mmol, 1.2 eq) was added dropwise and the mixture was stirred for 1 h at −78°C turning into a red solution. After addition of B(OMe)_3_ (0.67 mL, 6.0 mmol, 2.0 eq), the mixture was stirred for 1.5 h at room temperature. HCl 3 M (8 ml) was added, the phases were separated and the aqueous layer was washed with diethyl ether (10 mL). The mixture was brought to pH 7 with NaHCO_3_ forming a yellow precipitate. The precipitate was filtered and the collected solid was recrystallized from acetone and n‐hexane. TLC: R_f_ = 0.32, strong tailing (CH_2_Cl_2_/methanol 95:5). Yellow solid, mp 220°C (decomposition), yield 223 mg (30%). C_9_H_7_BBrNO_2_ (251.9 g/mol). Purity (HPLC): 97.8%, t_R_ = 16.11 min. HRMS (APCI): *m/z* = 251.9800 (calcd. 251.9826 for C_9_H_8_^11^B^79^BrNO_2_^+^ [M + H]^+^). ^1^H NMR (600 MHz, CD_3_OD): δ [ppm] = 7.84 (dd, *J* = 8.6/5.2 Hz, 1H, 3‐H), 7.89 (d, *J* = 7.5 Hz, 1H, 7‐H), 7.99 (d, *J* = 7.6 Hz, 1H, 6‐H), 8.85 (dd, *J* = 8.5/1.6 Hz, 1H, 4‐H), 9.38 (dd, *J* = 5.3/1.6 Hz, 1H, 2‐H). A signal for the OH proton is not seen in the 1H NMR spectrum. ^13^C NMR (151 MHz, CD_3_OD): δ [ppm] = 121.5 (1 C, C‐5), 123.0 (1 C, C‐3), 129.4 (1 C, C‐4a), 133.4 (1 C, C‐7), 135.4 (1 C, C‐6), 142.2 (1 C, C‐4), 143.0 (1 C, C‐8), 145.9 (1 C, C‐2), 147.2 (1 C, C‐8a). FT‐IR: ṽ [cm^−1^] = 3356 (O–H), 1578, 1497 (C═C_arom_), 1350 (O–H_bend_), 779 (C–Br).

#### 4.1.18 Hydrolysis Quantification of **3d** via UV‐Vis

To quantify the hydrolysis in aqueous solution, a 50 µM sample of **3d** was prepared from a 10 mM stock solution in dry DMSO (Thermo Scientific, Dreieich, Germany). To get to 5% DMSO, 5 µL of the stock solution were diluted with 45 µL of dry DMSO and 950 µL of Dulbecco's Phosphate Buffered Saline (DPBS) buffer pH 7.45 (Sigma‐Aldrich, St. Louis, USA). The resulting solution was transferred to High Precision SUPRASIL quartz glass cuvettes (Hellma Analytics GmbH, Müllheim, Germany) and placed directly in the light path of the fiber‐optics spectrometer setup from Avantes (AVASPEC─HSC1024X58TEC─EVO spectrometer and AVALIGHT‐DH‐S‐BAL light source) at room temperature. Continuous UV‐Vis spectra (250–900 nm) were recorded every 60 s for 89 min in total. For analysis of the linear decay within the first 30 min, the absorbance values for the *n* → π* transition at 421 nm (A_421 nm_) divided by the initial value at the start of the measurement (A^0^_421 nm_) were transformed by the natural logarithm and subsequently plotted and analyzed using OriginPro (version 2026, OriginLab Corporation, Northampton, MA, USA). The absorbance is proportional to the concentration (c) of **3d**. To determine the half‐life (*t*_1/2_), data were fitted to first‐order kinetics using the following equations for linear regression:

$$\ln\left(c\right)=\ln\left(c_{0}\right)\;*-k\;*\;t$$

$$t_{1/2}=\frac{\ln(2)}{k}$$

Graphical results are summarized in Figure 2, while fitting parameters of linear regression are given in Supporting Information: Table 1.

#### 4.1.19 Hydrolysis Quantification of **3d** via ^1^H‐NMR

For hydrolysis quantification via ^1^H‐NMR, spectra were measured at room temperature on a Bruker Avance Neo 400 spectrometer (Billerica, USA) at 400 MHz. The chemical shift “δ” is given in ppm (parts per million) against tetramethylsilane (TMS). Spectra were analyzed utilizing the software MestReNova (v17.0.0‐41178,® 2014 by Mestrelab Research S.L.). DMSO‐d_6_ purchased from Sigma Aldrich (St. Louis, United States of America) and D_2_O purchased from Deutero (Kastellaun, Germany) were used as references. For sample preparation, 14.8 mg of **3d** were dissolved in a mixture of 0.66 mL DMSO‐d_6_ and 0.14 mL D_2_O (17.5% D_2_O, *c*_**3d**_ = 56.5 mM). Between sample preparation and first NMR measurement, 12 min passed. Measurements were taken every 10 min, resulting in a total of 7 data points. Due to the large molar excess of water in the reaction and the known behavior of the kinetics from the UV data, the H_2_O peak was utilized as an internal standard for integral normalization. Kinetic analysis based on proton integrals was performed using natural logarithm transformations of the integral for the proton in 4‐position of **3d** (I_**3d**_) relative to the integral of water (I_H2O_) and subsequent linear regression fitting as previously described for UV‐Vis data (Section 4.1.18). The results are summarized in Figure 2 and Supporting Information: Table 1.

### 4.2 Pharmacological/Biological Assays

#### 4.2.1 Molecular Biology, cRNA Injection and Two‐Electrode Voltage Clamp (TEVC) Recordings

Molecular biology, cRNA injection and TEVC recordings were performed as previously described [30]. Briefly, cRNA was generated from linearized wildtype hK_2P_18.1/pSGEM (restriction enzyme NheI) using the mMessage mMachine T7 kit (Invitrogen) according to the manufacturer's specifications. For hK_2P_18.1 channel expression, 1 ng of cRNA was injected into *X. laevis* oocytes (Ecocyte Bioscience, Dortmund, Germany) followed by 24–48 h of incubation at 18°C in Bath's solution containing 88 mM NaCl, 1 mM KCl, 0.4 mM CaCl_2_, 0.33 mM Ca(NO_3_)_2_, 0.6 mM MgSO_4_, 5 mM TRIS‐HCl, 2.4 mM NaHCO_3_, 80 mg/L theophylline, 63 mg/L benzylpenicillin, 40 mg/L streptomycin, and 100 mg/L gentamycin. After incubation and sufficient hK_2P_18.1 channel expression, TEVC recordings were conducted at room temperature using a Turbo Tec 10CX amplifier (NPI electronic, Tamm, Germany), a NI USB 6221 DA/AD Interface (National Instruments, Austin, USA), GePulse software for data acquisition (Dr. Michael Pusch, Genova, Italy) and glass capillaries (Harvard Apparatus) backfilled with 3 M KCl (0.5–1.5 MΩ). Oocytes expressing hK_2P_18.1 were placed in a perfusion chamber filled with wash solution containing 95.4 mM NaCl, 2 mM KCl, 1.8 mM CaCl_2_ and 5 mM HEPES (pH 7.4, adjusted by NaOH). For recordings, recording solution containing 17.4 mM NaCl, 80 mM KCl, 1.8 mM CaCl_2_ and 5 mM HEPES (pH 7.4, adjusted by NaOH) used. To ensure unbiased results, recording solution was supplemented with 0.1% DMSO. Compound solutions were prepared from 100 mM DMSO stocks diluted 1:1000 with recording solution (final compound concentration 100 µM, 0.1% DMSO). To evaluate activity, a repetitive pulse sequence (sweeps) was applied, where each sweep is composed of 2 s at 0 mV, 0.5 s at −100 mV and 2 s at 0 mV (see pulse protocol Figure 1). The previously mentioned solutions were applied sequentially according to the following scheme: sweep 1–15 wash solution (2 mM K^+^), sweep 16–35 recording solution (80 mM K^+^ + 0.1% DMSO), sweep 36–65 compound solution (100 µM compound in recording solution), sweep 66–85 recording solution (80 mM K^+^ + 0.1% DMSO), sweep 86–100 wash solution (2 mM K^+^). The resulting current at −100 mV under the influence of the compound (I_c_) was calculated by the following equation:

$$I_{c}=\frac{I_{\mathrm{sweep}\;65}}{I_{\mathrm{sweep}\;35}}$$

All pharmacological data are summarized in Table 1 as mean ± SEM calculated from n independent oocytes. For significant ion channel current modulation, the fraction of effect washout was determined by the following equation:

$$I_{w}\left[\%\right]=\left(1-\frac{I_{\mathrm{sweep}\;85}-I_{\mathrm{sweep}\;35}}{I_{\mathrm{sweep}\;65}-I_{\mathrm{sweep}\;35}}\right)*100\;$$

Activity data were statistically evaluated using OriginPro 2026. To ensure symmetry of activation/inhibition effects as well as the applicability of general linear regression model with categorical data (compounds), single I_c_ values were log_2_‐transformed and mean values were determined together with lower (LCI) and upper (UCI) limits of 95% confidence intervals for each compound. Transformed log_2_ values correspond to no modulation of ion channel activity for log_2_ (I_c_) = 0, to activation of ion channel for log_2_ (I_c_) > 0 and to ion channel inhibition for log_2_ (I_c_) < 0. Significance of log_2_ (I_c_) ≠ 0 was determined utilizing the 95% confidence interval and is indicated by ns *p* > 0.05, * for *p* < 0.05 and **p < 0.01.

### 4.3 Procedure for **3d** and **4b** Docking Poses

For docking poses, compounds **3d** and **4b** were imported via a SMILES file into the YASARA structure 25.12.1 software [60]. After import, H atoms were added to the structure and initial structure optimization via semi‐empirical quantum mechanics (MOPAC) was performed to generate lowest energy rotamer conformation in water (TIP3P model). The rotamer was saved and used as starting point for docking experiments. The docking procedure was conducted as previously described with the same hK_2P_18.1 channel model as used for cloxyquin docking [30]. Briefly, YASARA provided macro “dock_runscreening.mcr” was used for local docking with the following adaptations: 100 runs per ligand, AMBER15IPQ force field, docking box dimensions of 20 × 15 × 20 Å around the cloxyquin binding site and ranking of docking poses by binding energy. The best poses for **3d** and **4b** were used for comparison. For overlay of **3d** with cloxyquin, previously published clox‐2 binding pose and MUSTANG algorithm were used [30, 61].

### 4.4 Use of Artificial Intelligence Generated Content (AIGC)

The authors declare that artificial intelligence was only used to create initial draft of the graphical abstract, that was later specified and corrected by the corresponding author. No other parts of the manuscript were supported by any artificial intelligence.

## Funding

The authors have nothing to report.

## Conflicts of Interest

The authors declare no conflicts of interest.

## Data and Code Availability

All relevant data including chemical procedures, ^1^H‐ and ^13^C─NMR spectra, purity data (HPLC, quantitative ^1^H‐NMR) and sample traces for each compound are given in the main article or the Supplementary Information. All used software is either commercially (OriginPro 2026, YASARA structure 25, Microsoft Excel and Word) or freely (Gepulse, Ana; http://users.ge.ibf.cnr.it/pusch/programs-mik.htm) available. All codes were used as provided by the software, adaptions are given in the materials and methods sections. No individual code was used. Further data is available from the corresponding author on reasonable request.

## Supporting information


Supporting File 1

Supporting File 2

## Data Availability

The data that support the findings of this study are available from the corresponding author upon reasonable request.
