# Supplementary material for: Evaluation of Novel Cloxyquin Analogs for K2P18.1 Channel Modulation
Source: Arch Pharm (Weinheim). 2026 Jul 15;359(7):e70310. doi: 10.1002/ardp.70310 (PMC13373494; doi:10.1002/ardp.70310)
Supplement: Supplementary file 1 — Supporting File 1 [file ARDP-359-e70310-s002.docx]

**Supporting Information**

**Evaluation of novel cloxyquin analogs for K_2P_18.1 channel modulation**

Jasmin Sörgel^1)^, Marcel Kloth^2)^, Henning Klaasen^2)^, Sven G. Meuth^3)^, Thomas Budde^4)^, Bart Jan Ravoo^2),5)^, Julian A. Schreiber^1)*^

1. Institute of Pharmaceutical and Medicinal Chemistry, University of Münster, Corrensstraße 48, 48149 Münster, Germany.
2. Organisch-Chemisches Institut, University of Münster, Corrensstraße 36, 48149 Münster, Germany.
3. Department of Neurology, University Hospital Münster, 48149 Münster, Germany.
4. Institute of Physiology I, University of Münster, 48149, Münster, Germany.
5. Center for Soft Nanoscience, University of Münster, Busso-Peus-Straße 10, 48149 Münster, Germany.

ORCID

Marcel Kloth: 0009-0000-3737-2164

Henning Klaasen: 0000-0002-1930-5969

Sven G. Meuth: 0000-0003-2571-3501

Thomas Budde: 0000-0002-5263-8183

Bart Jan Ravoo: 0000-0003-2202-7485

Julian A. Schreiber: 0000-0002-3847-7539

Keywords: 8-Hydroxyquinolines, K_2P_ channels, cloxyquin, K_2P_18.1, TRESK.

*Corresponding author:

Dr. Julian Alexander Schreiber

Corrensstrasse 48

D-48149 Münster

Tel. +49-251-8333372

j.schreiber@uni-muenster.de

**CONTENT** **PAGE**

1H and 13C NMR spectra 2 - 15

Purity data (HPLC; quantitative ^1^H-NMR for **3d**) 16 – 29

SI Table 1: Fitting parameters for linear regression 30

TEVC sample traces 31 - 33


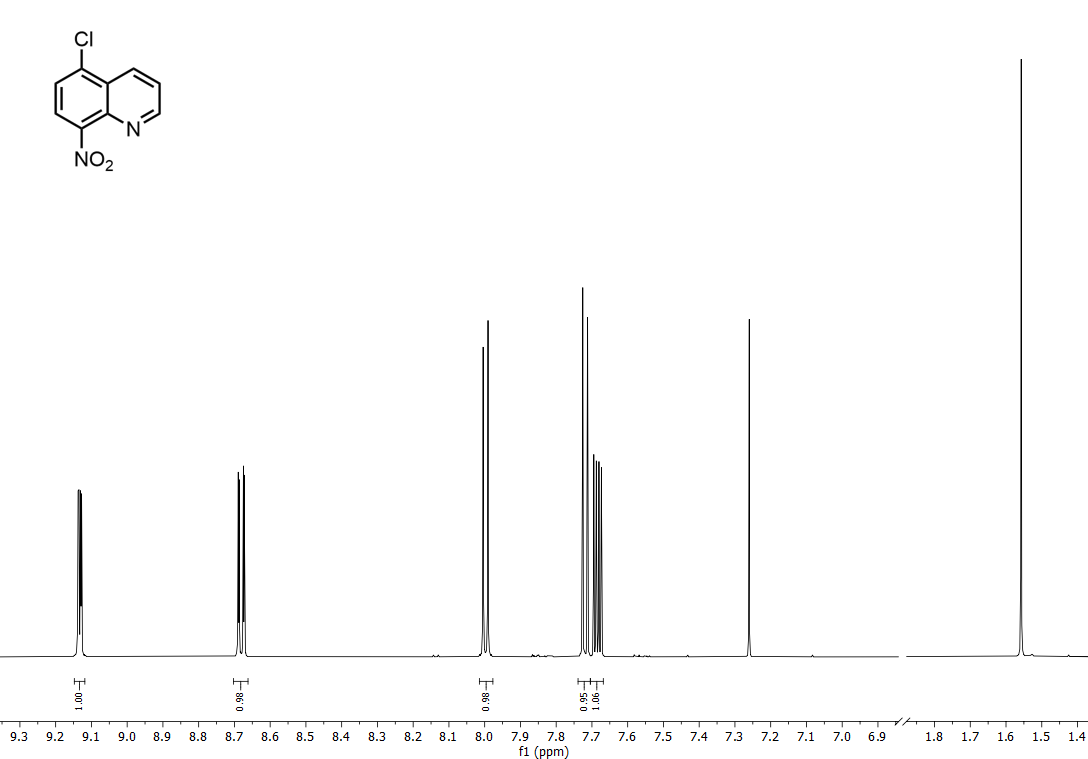


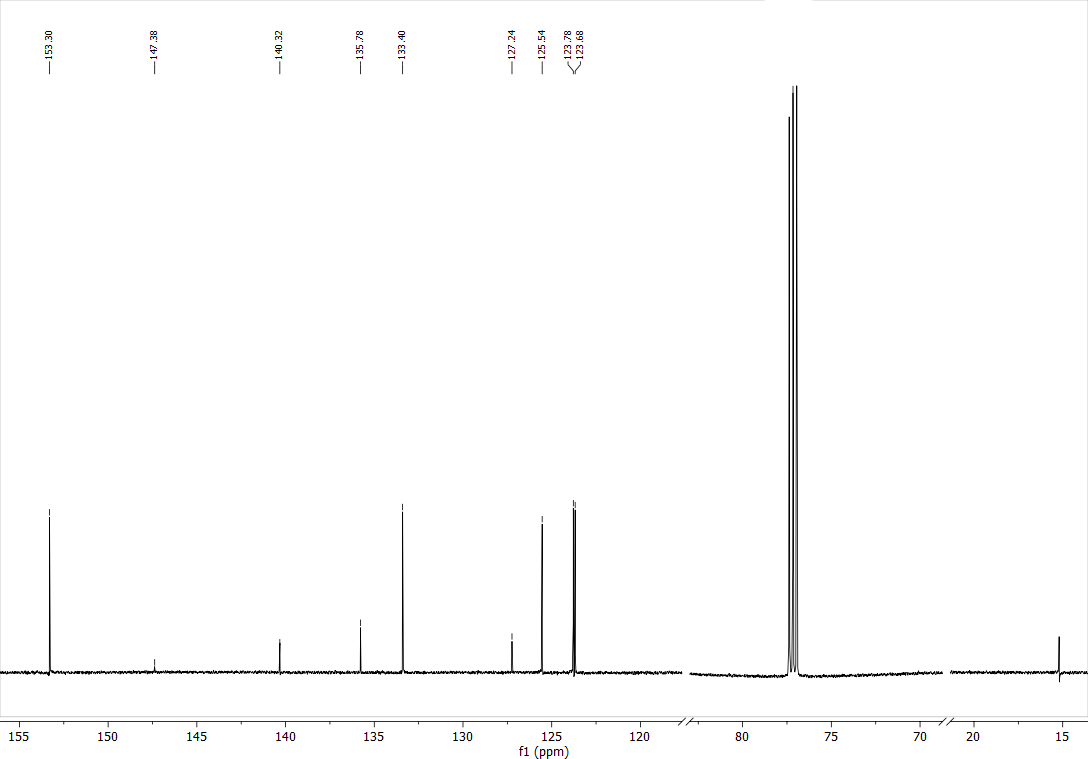


^1^H- (top) and ^13^C-NMR (bottom) spectra of 5-Chloro-8-nitroquinoline (**2a**) in CDCl_3_.


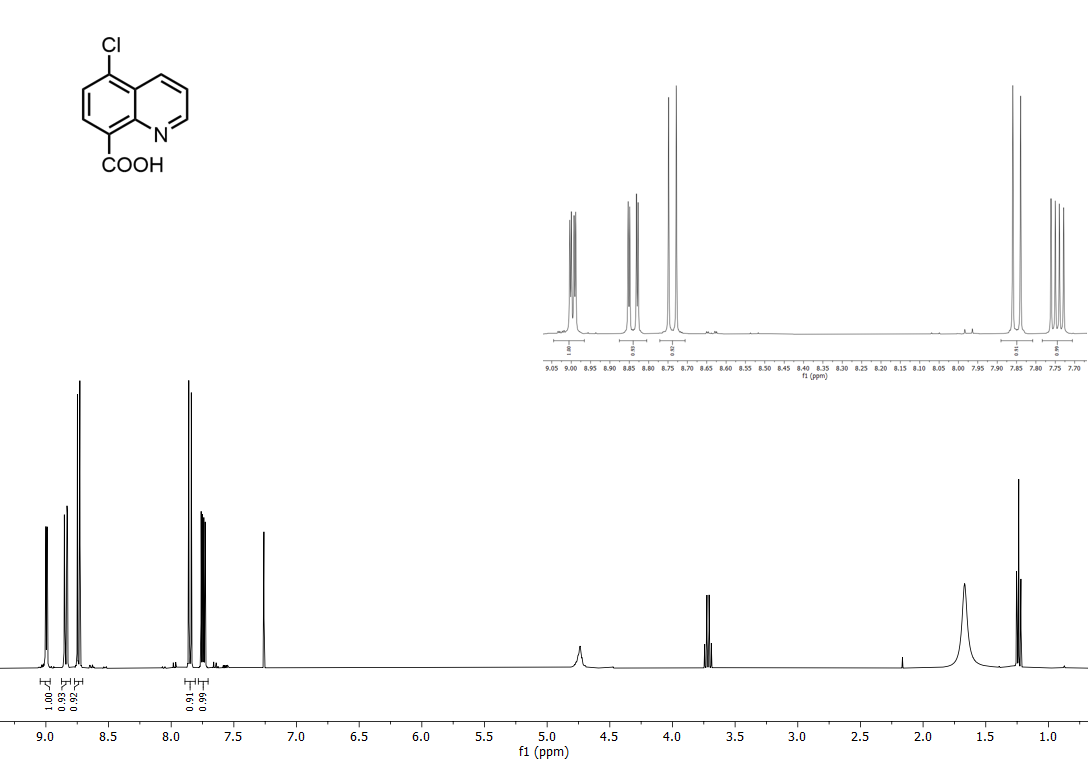


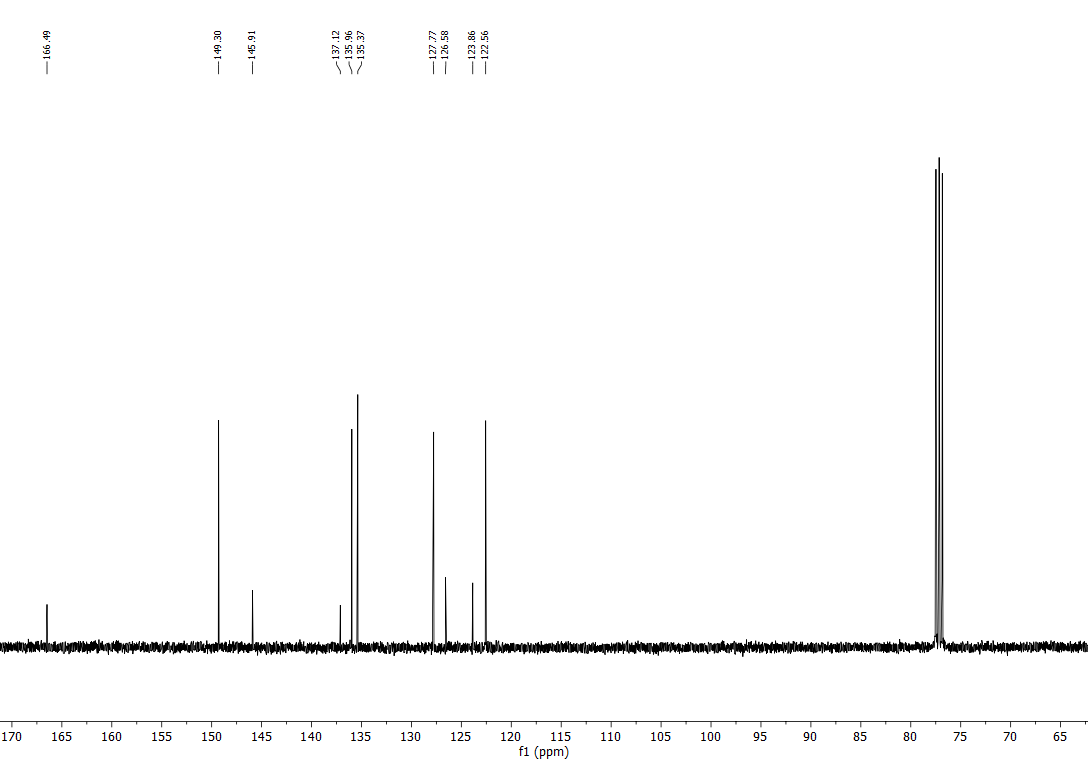


^1^H- (top) and ^13^C-NMR (bottom) spectra of 5-Chloroquinoline-8-carboxylic acid (**2b**) in CDCl_3_.

**
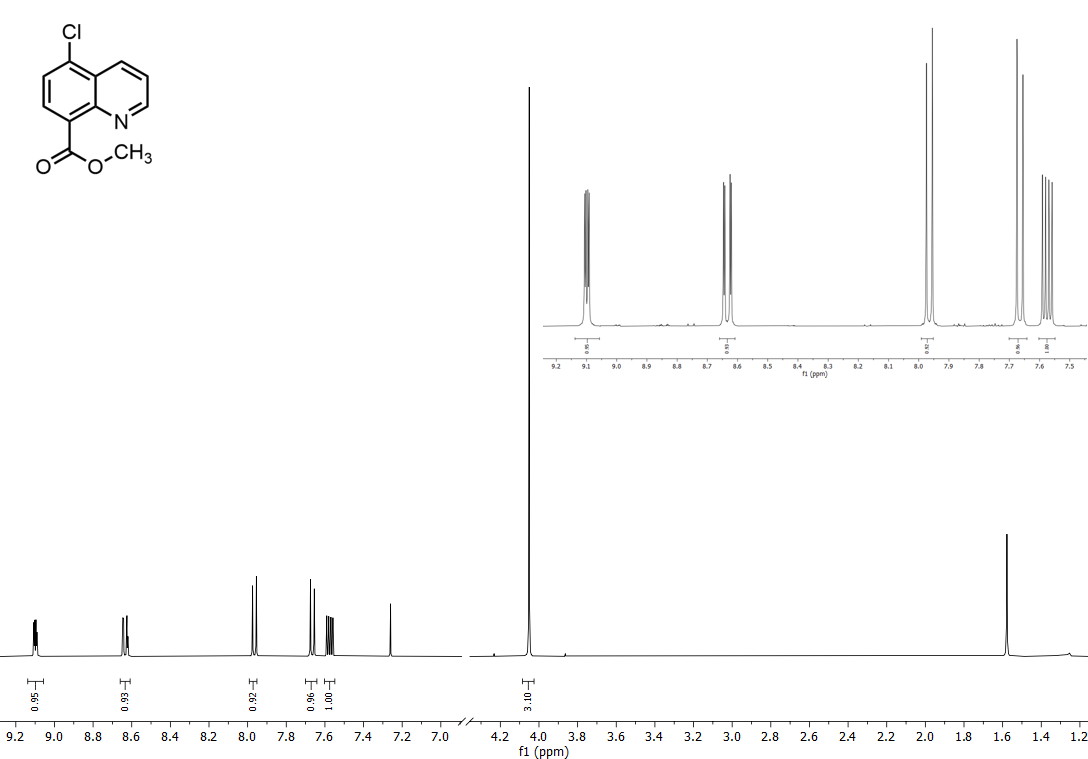
**

**
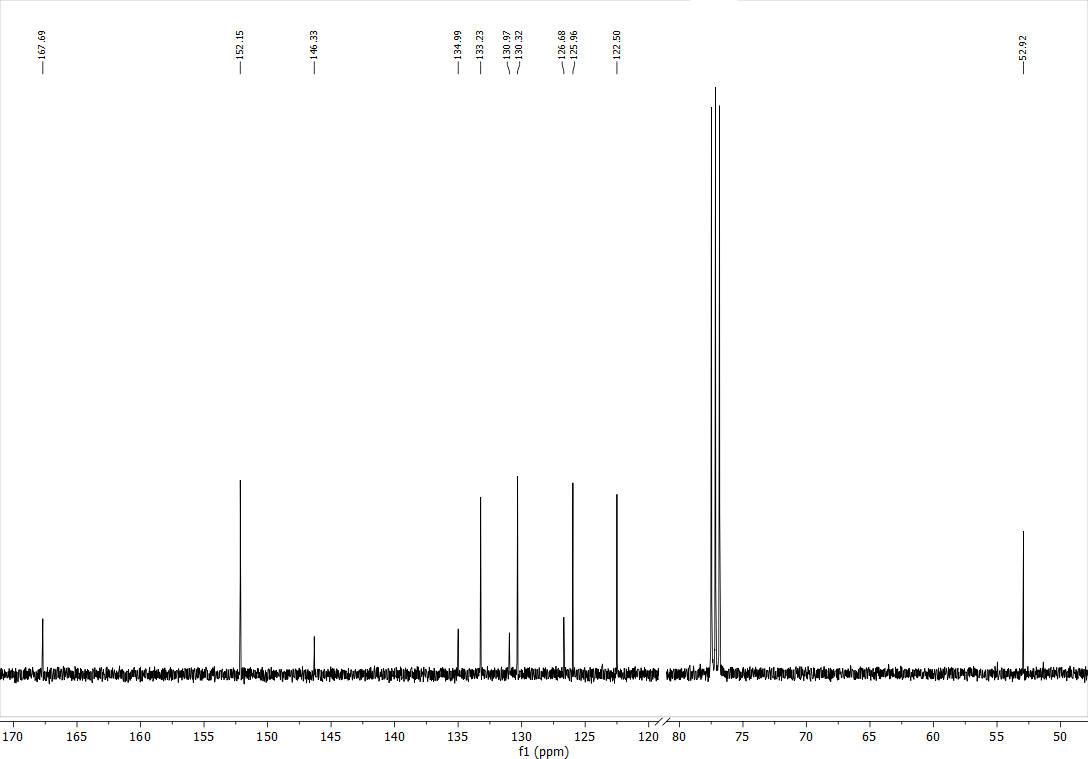
**

^1^H- (top) and ^13^C-NMR (bottom) spectra of Methyl 5-chloroquinoline-8-carboxylate (**2c**) in CDCl_3_.

**
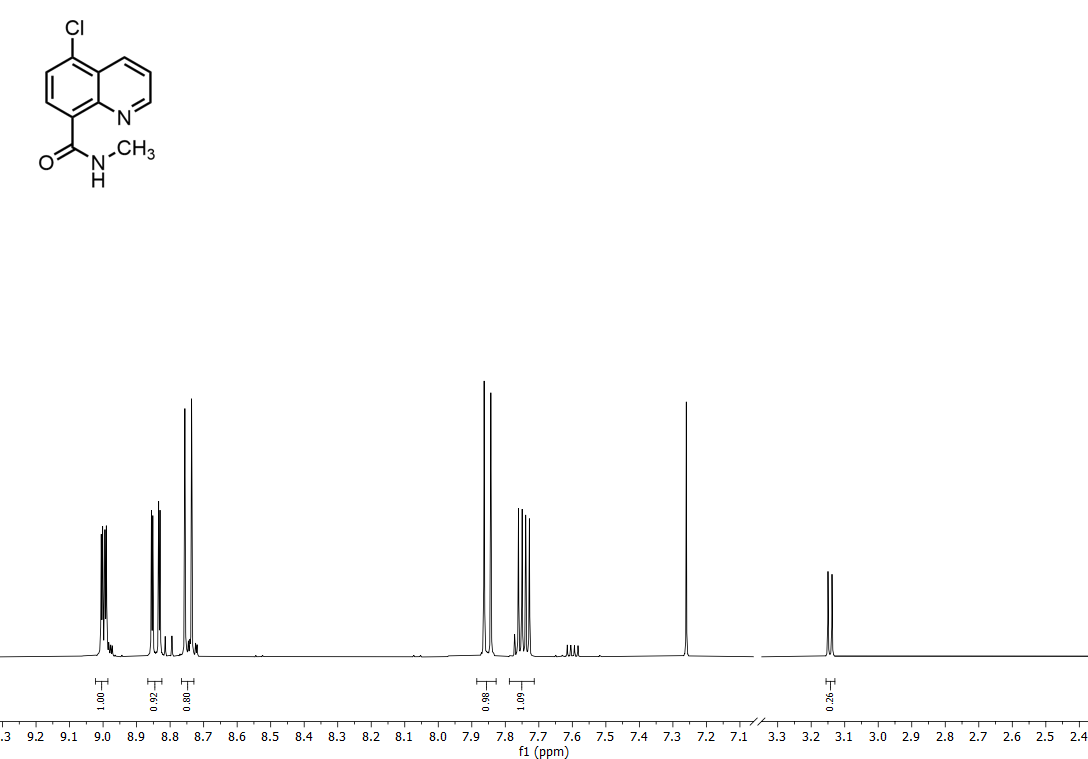
**

**
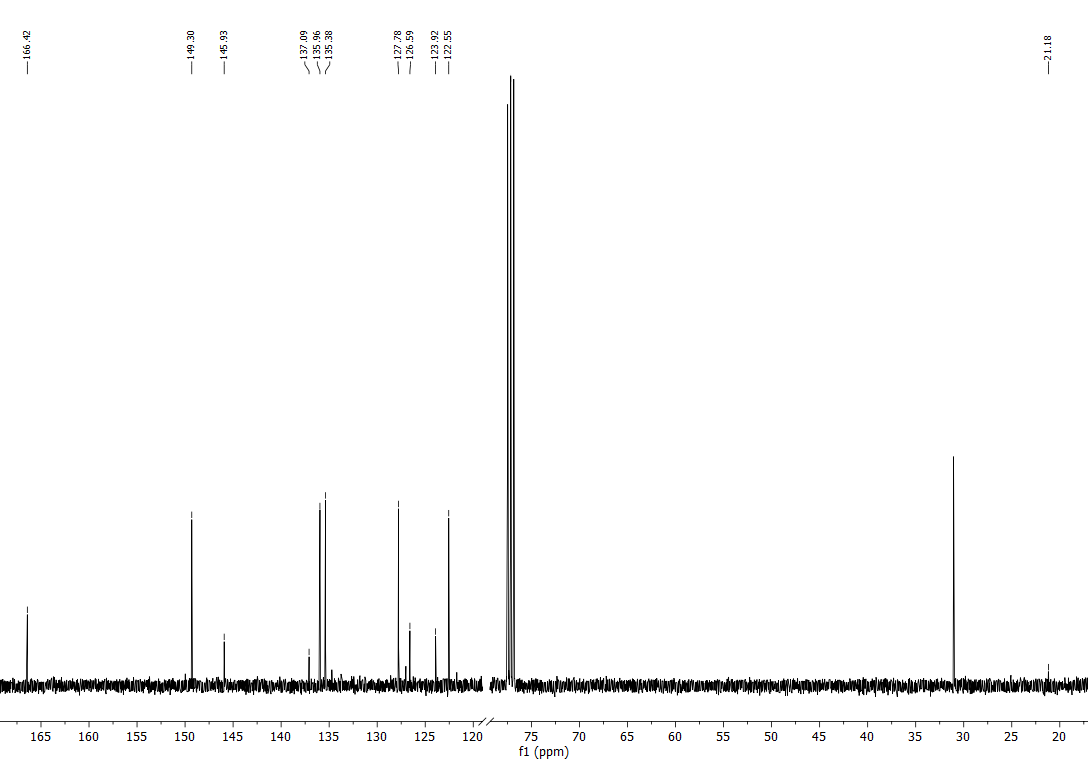
**

^1^H- (top) and ^13^C-NMR (bottom) spectra of 5-Chloro-*N*-methylquinoline-8-carboxamide (**2d**) in CDCl_3_.

**
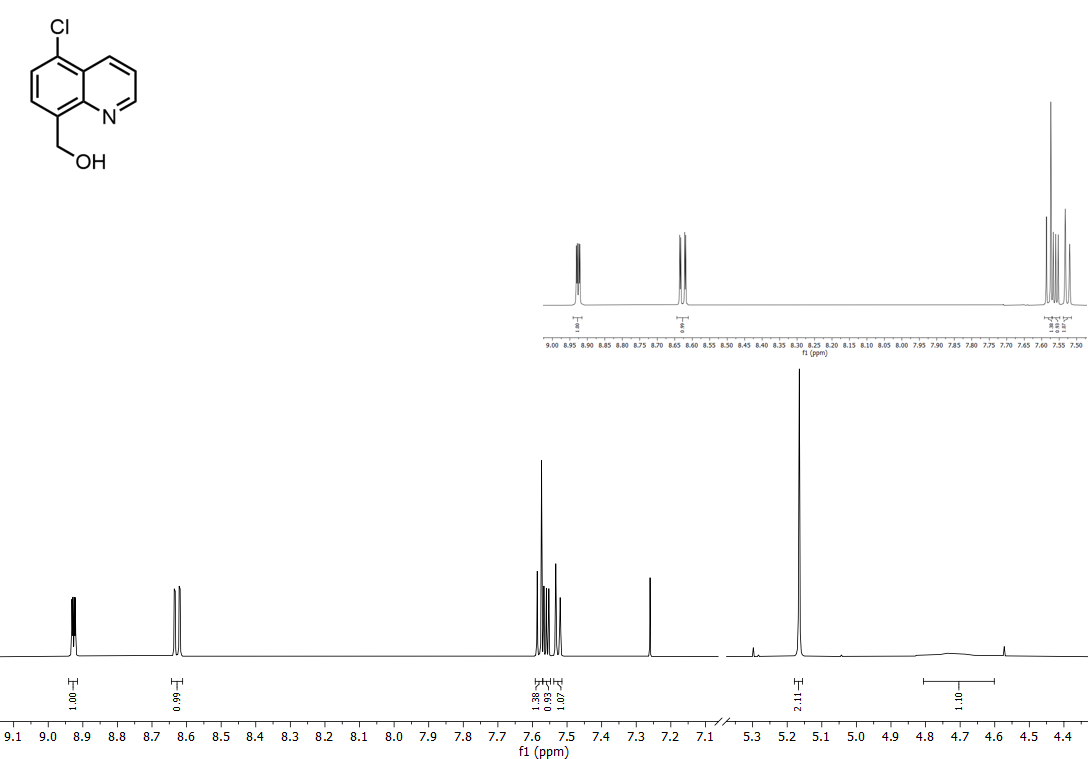
**

**
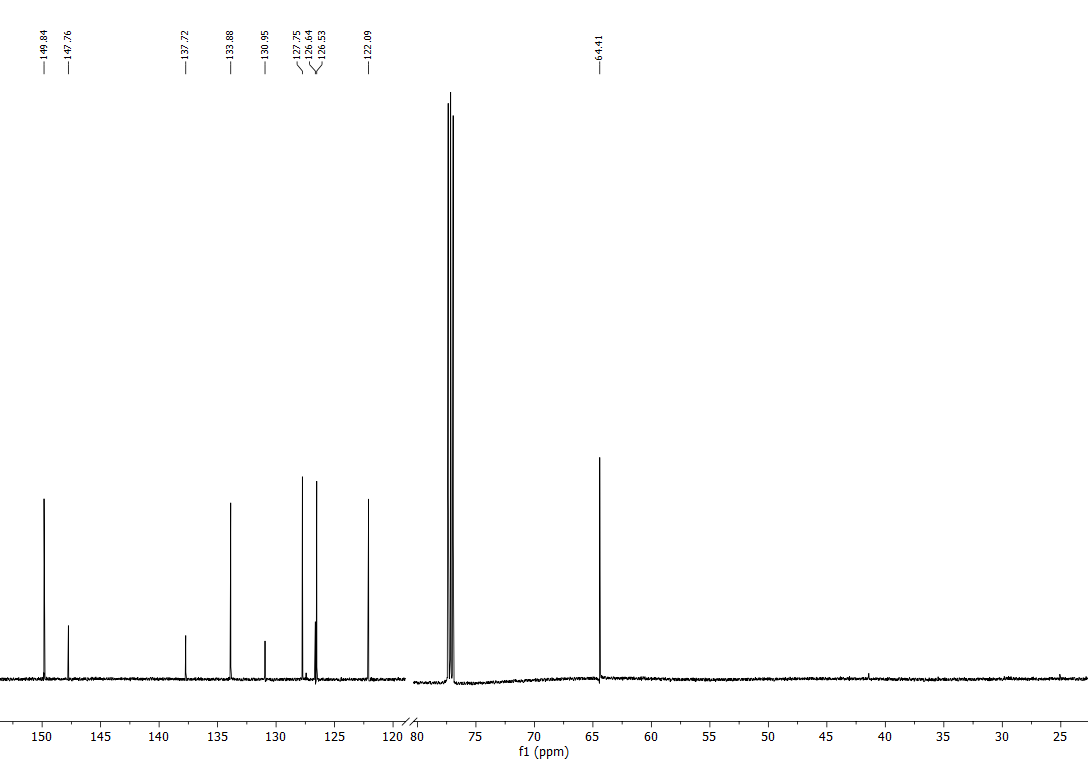
**

^1^H- (top) and ^13^C-NMR (bottom) spectra of (5-Chloroquinolin-8-yl)methanol (**2e**) in CDCl_3_.

**
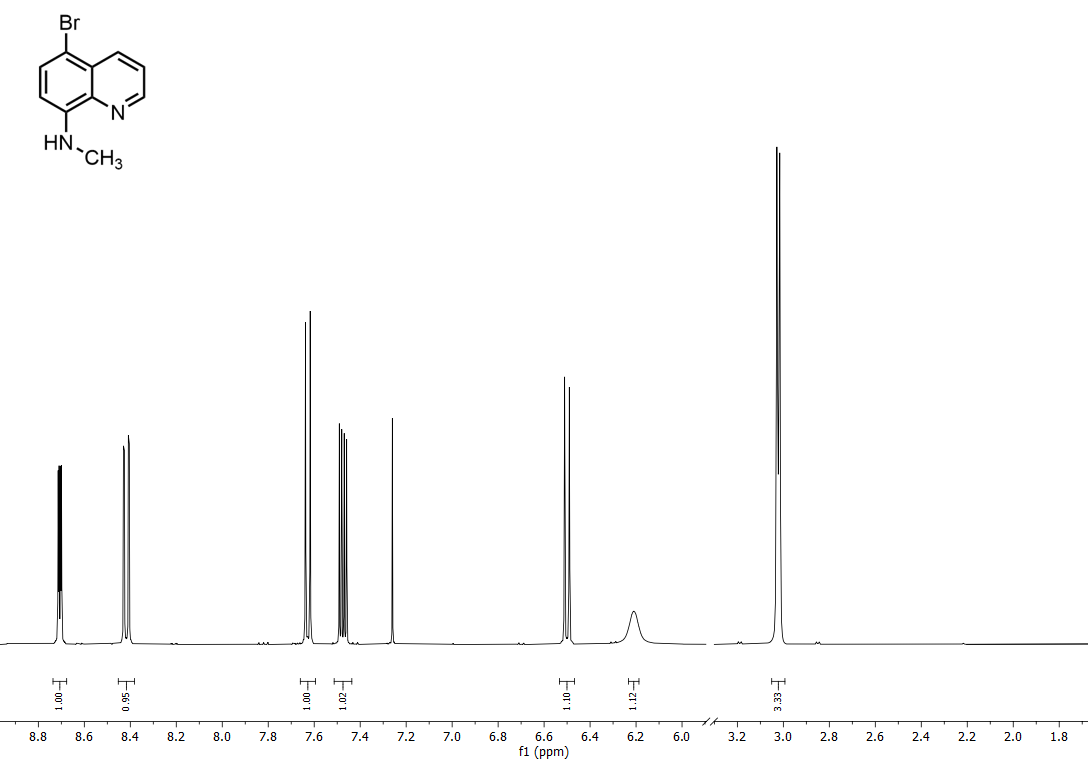
**

**
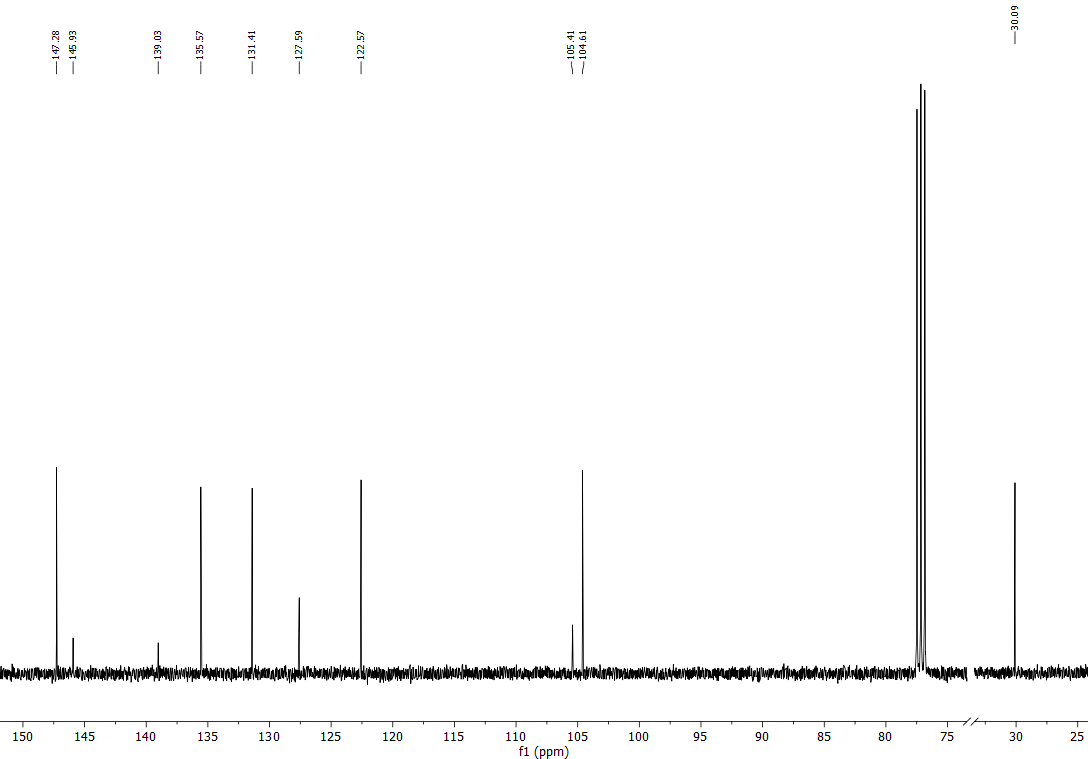
**

^1^H- (top) and ^13^C-NMR (bottom) spectra of 5-Bromo-*N*-methylquinoline-8-amine (**3b**) in CDCl_3_.

**
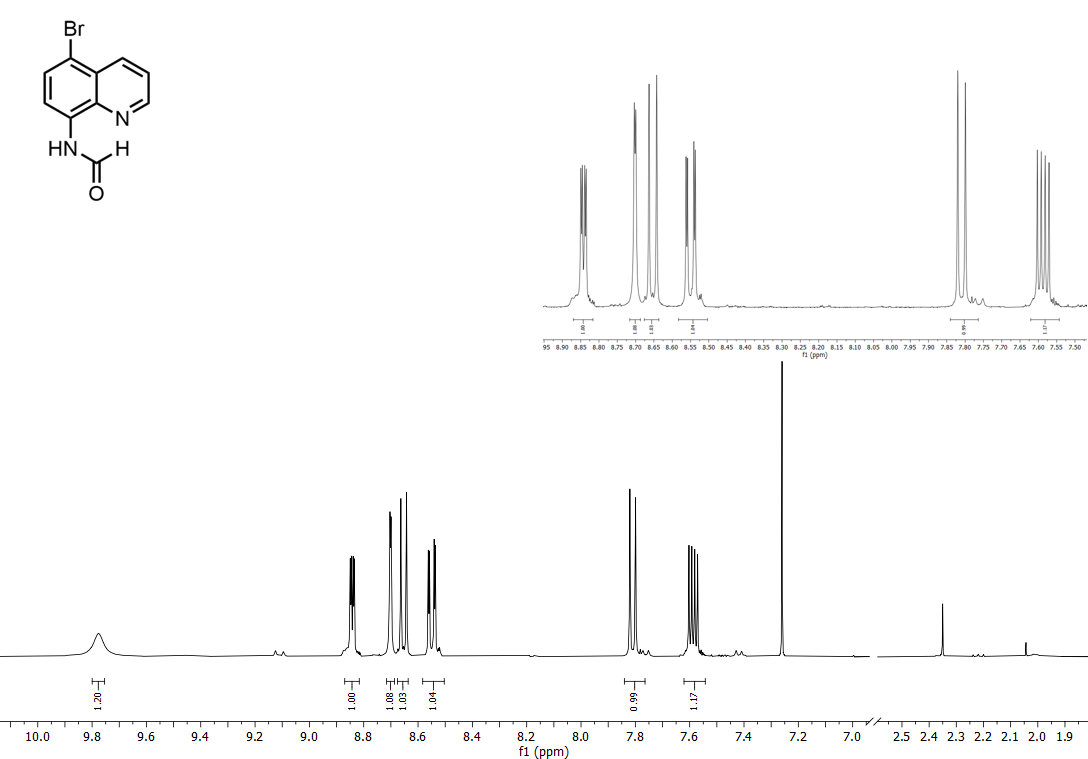
**

**
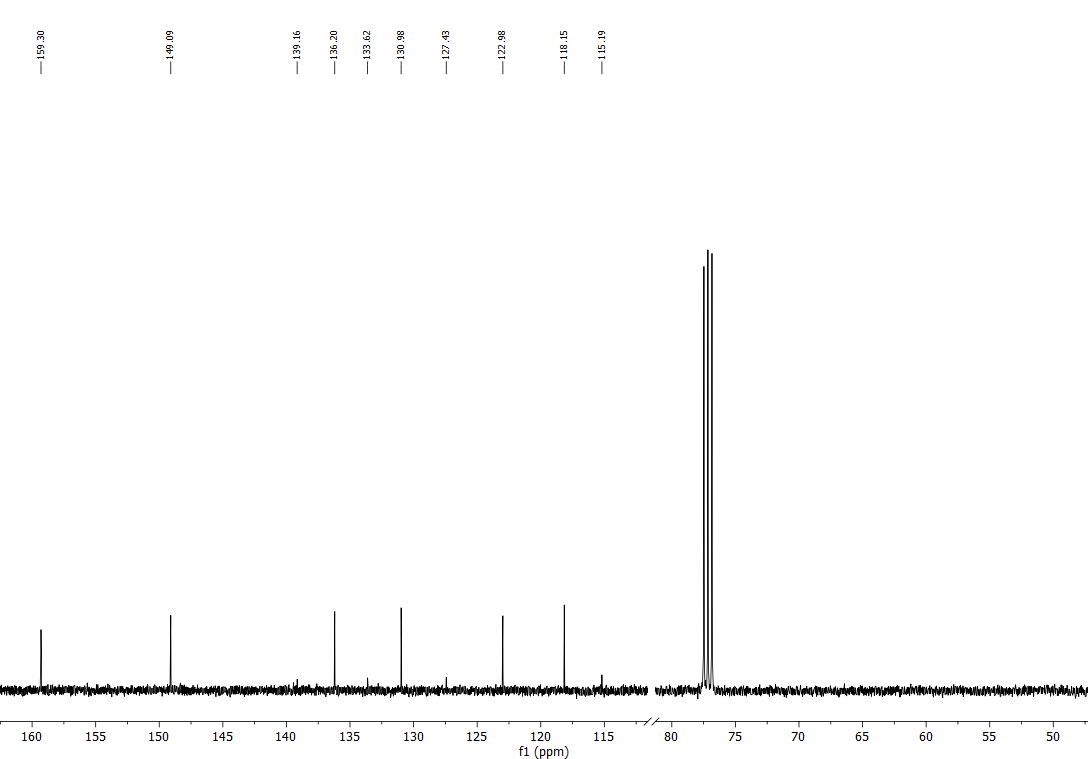
**

^1^H- (top) and ^13^C-NMR (bottom) spectra of *N*-(5-Bromoquinolin-8-yl)formamid (**3c**) in CDCl_3_.

**
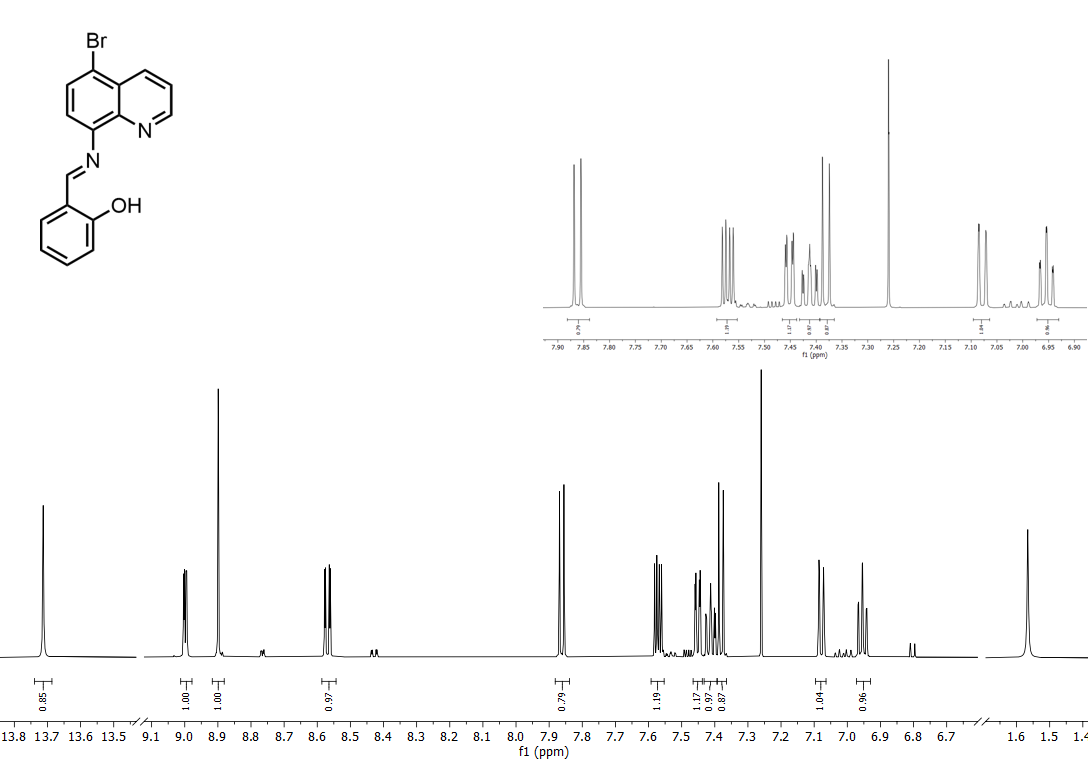
**

**
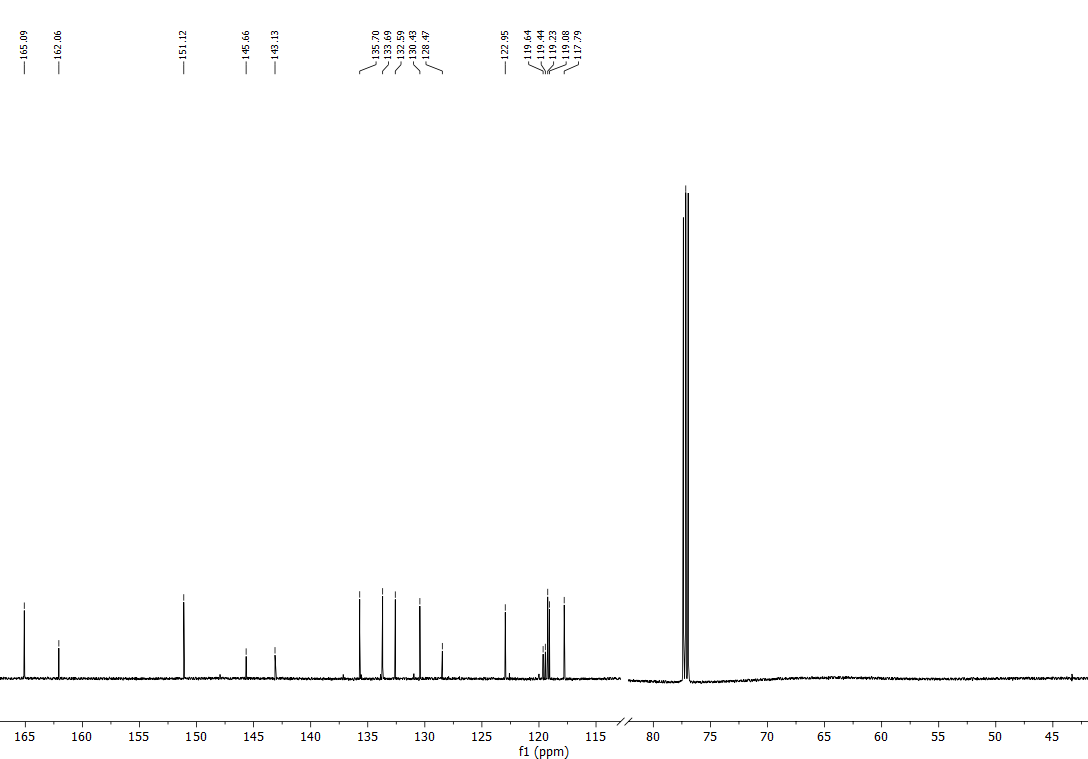
**

^1^H- (top) and ^13^C-NMR (bottom) spectra of *N*-(5-Bromoquinolin-8-yl)2-hydroxybenzaldimine (**3d**) in CDCl_3_.

**
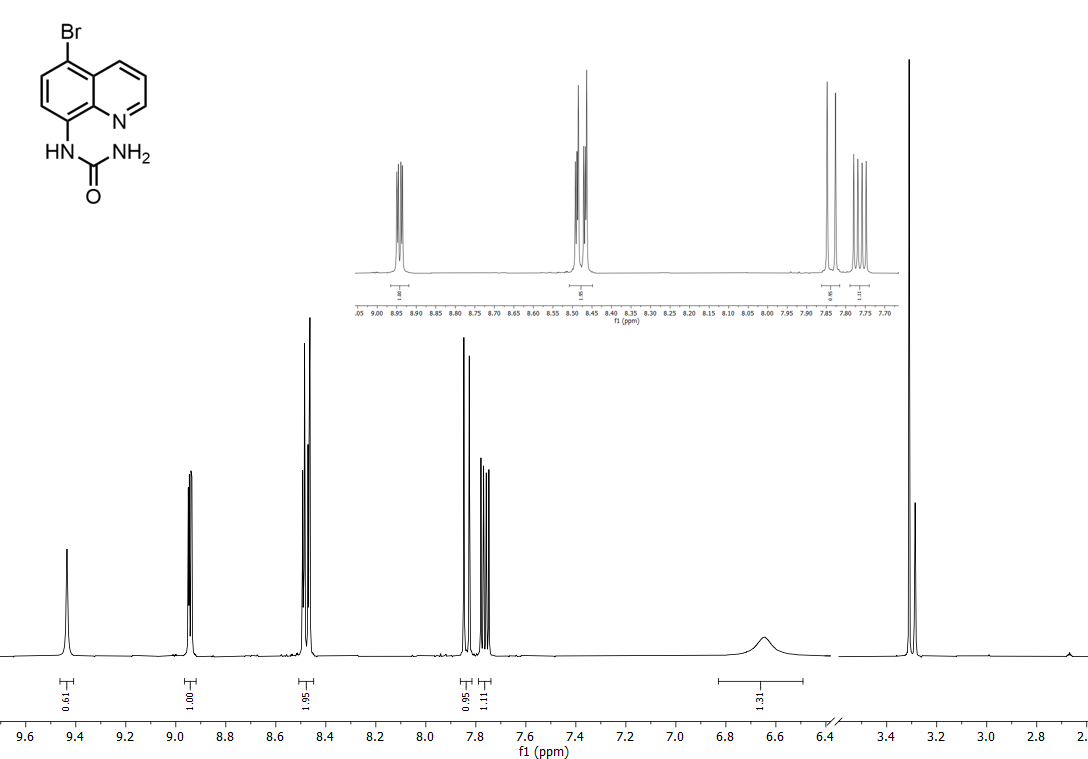
**

**
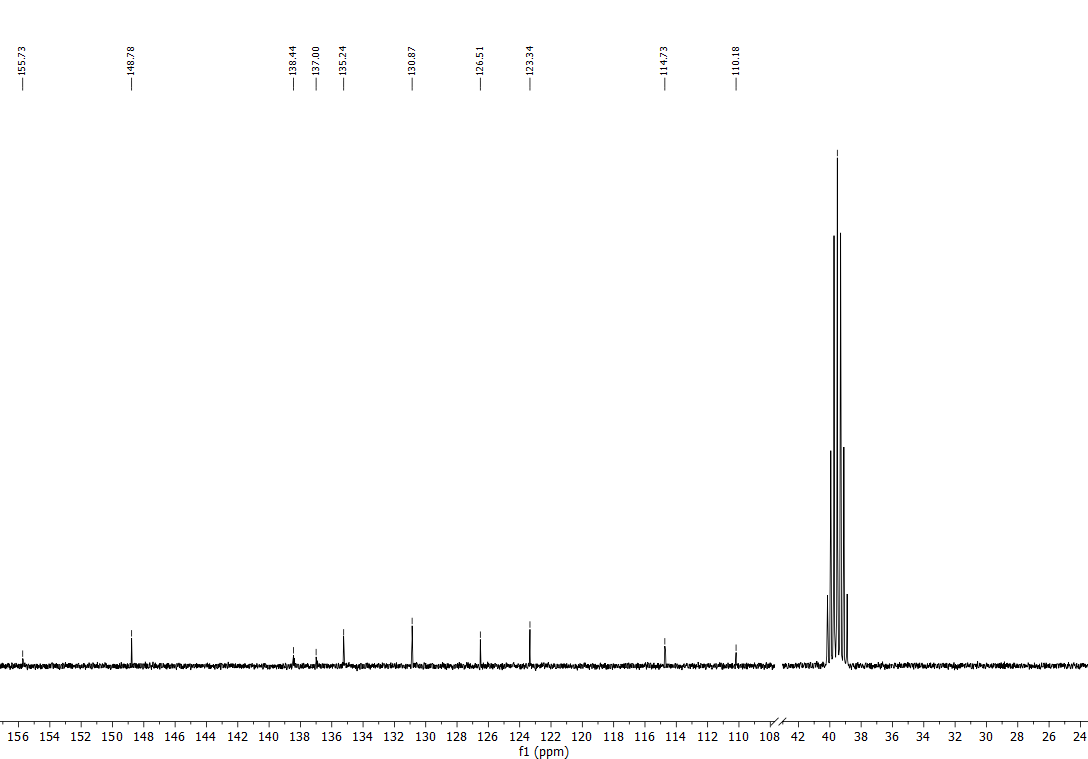
**

^1^H- (top) and ^13^C-NMR (bottom) spectra of 1-(5-Bromoquinolin-8-yl)urea (**3e**) in DMSO-D_6_.

**
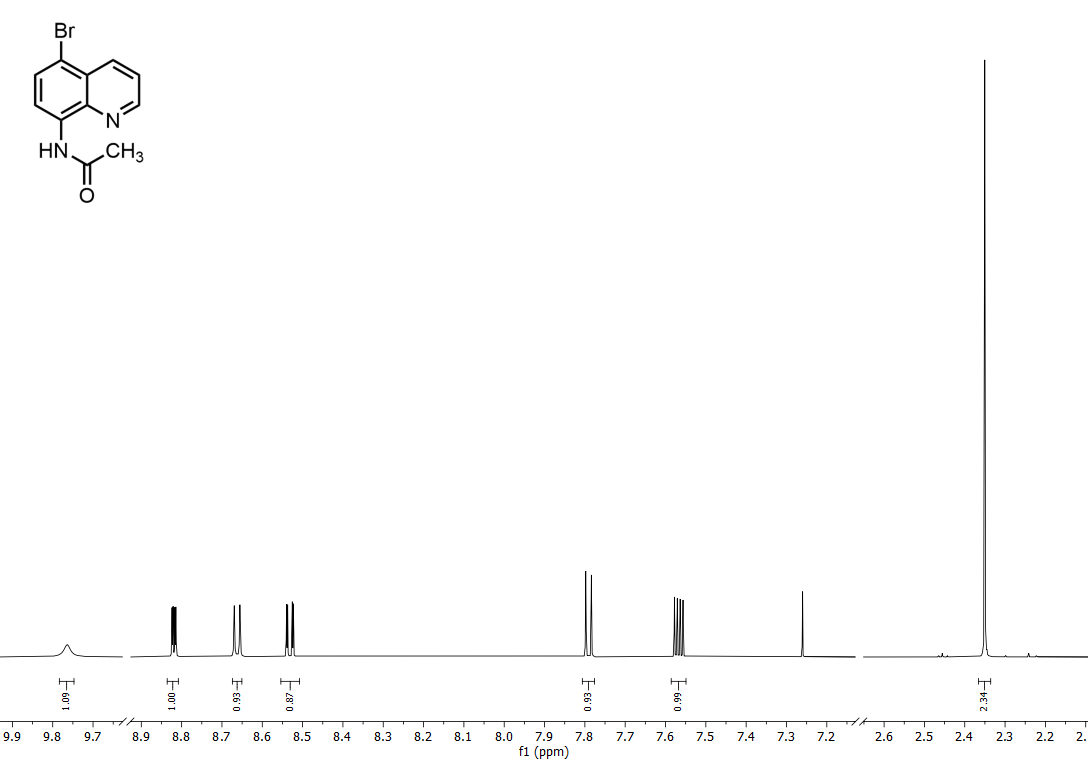
**

**
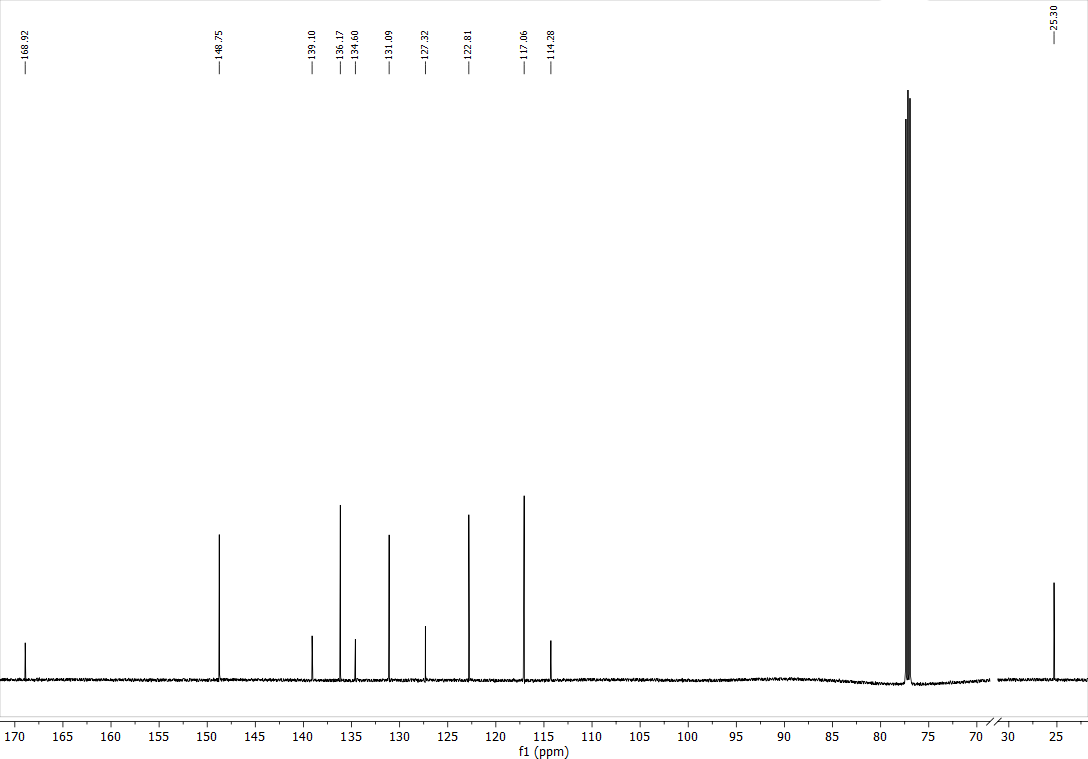
**

^1^H- (top) and ^13^C-NMR (bottom) spectra of *N*-(5-Bromoquinolin-8-yl)acetamid (**3f**) in CDCl_3_.

**
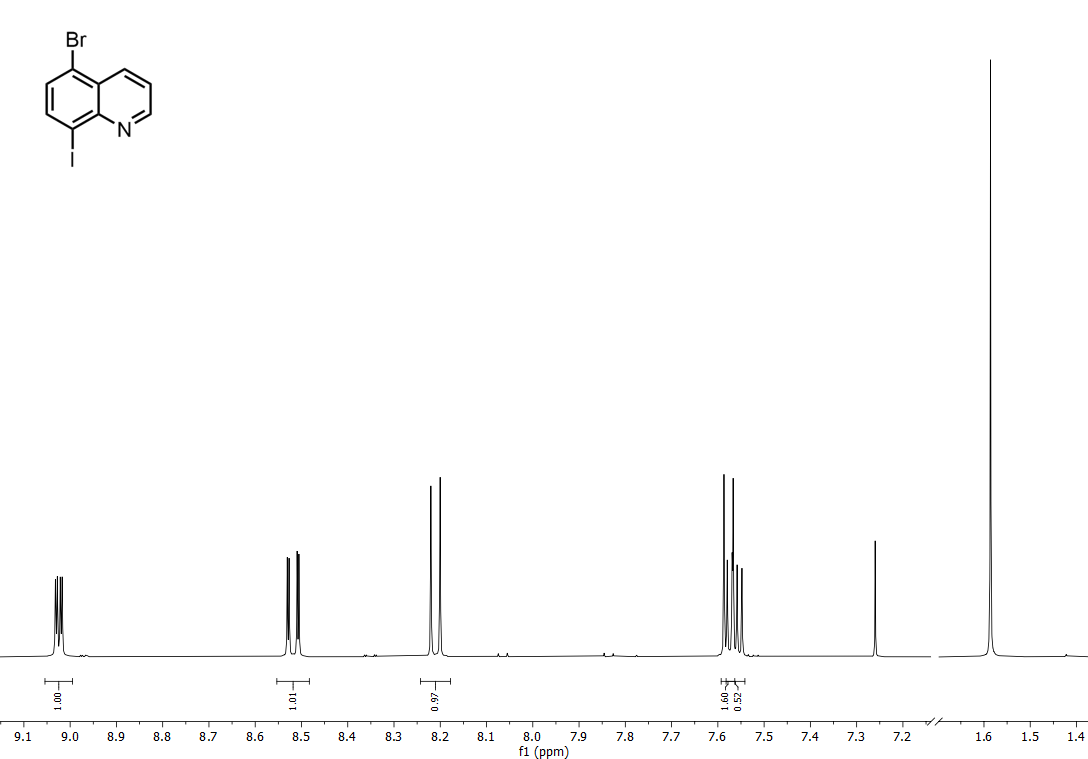
**

**
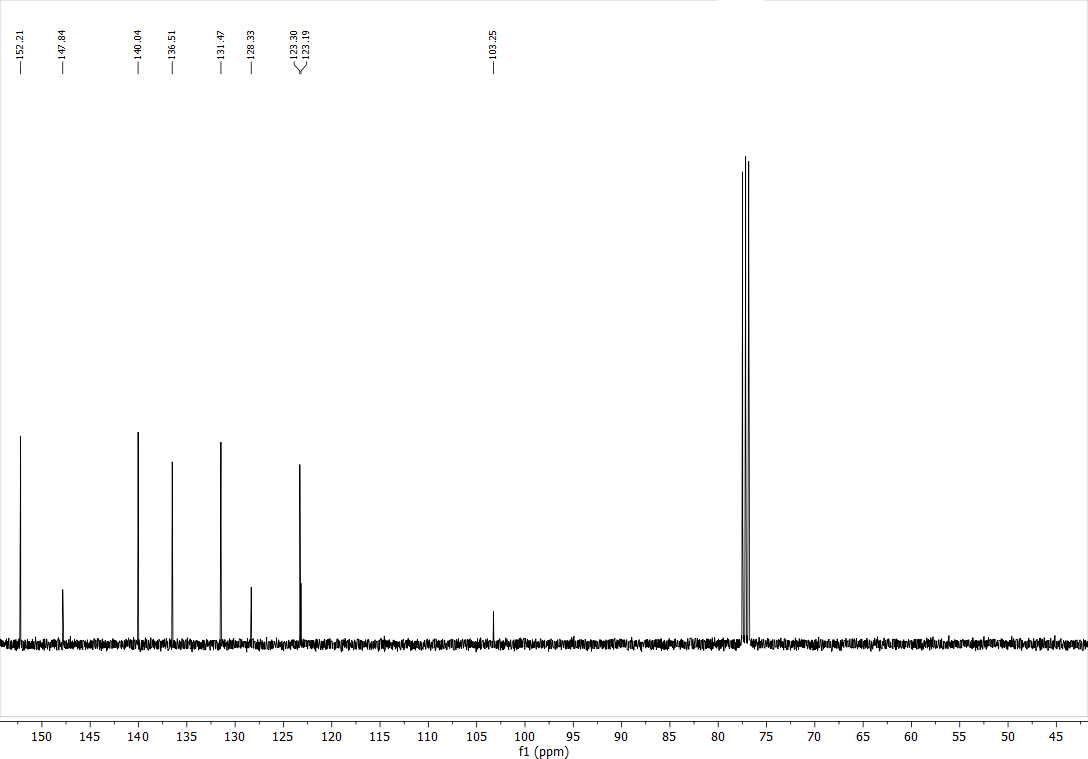
**

^1^H- (top) and ^13^C-NMR (bottom) spectra of 5-Bromo-8-iodoquinoline (**3g**) in CDCl_3_.

**
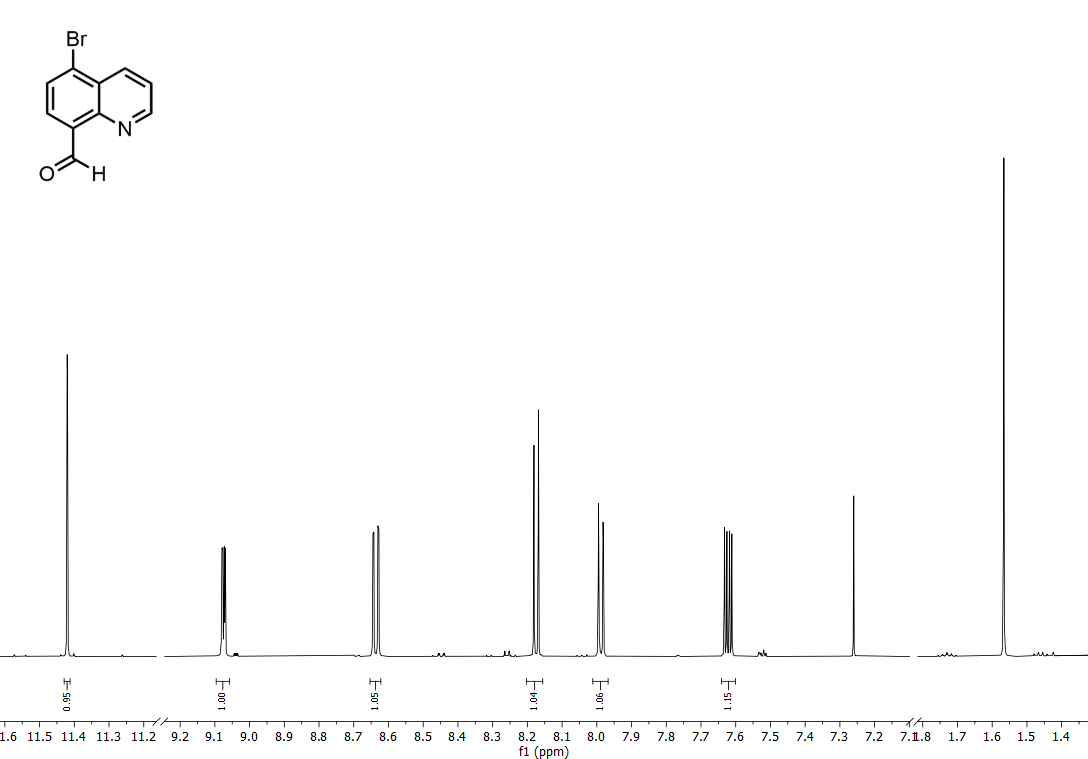
**

**
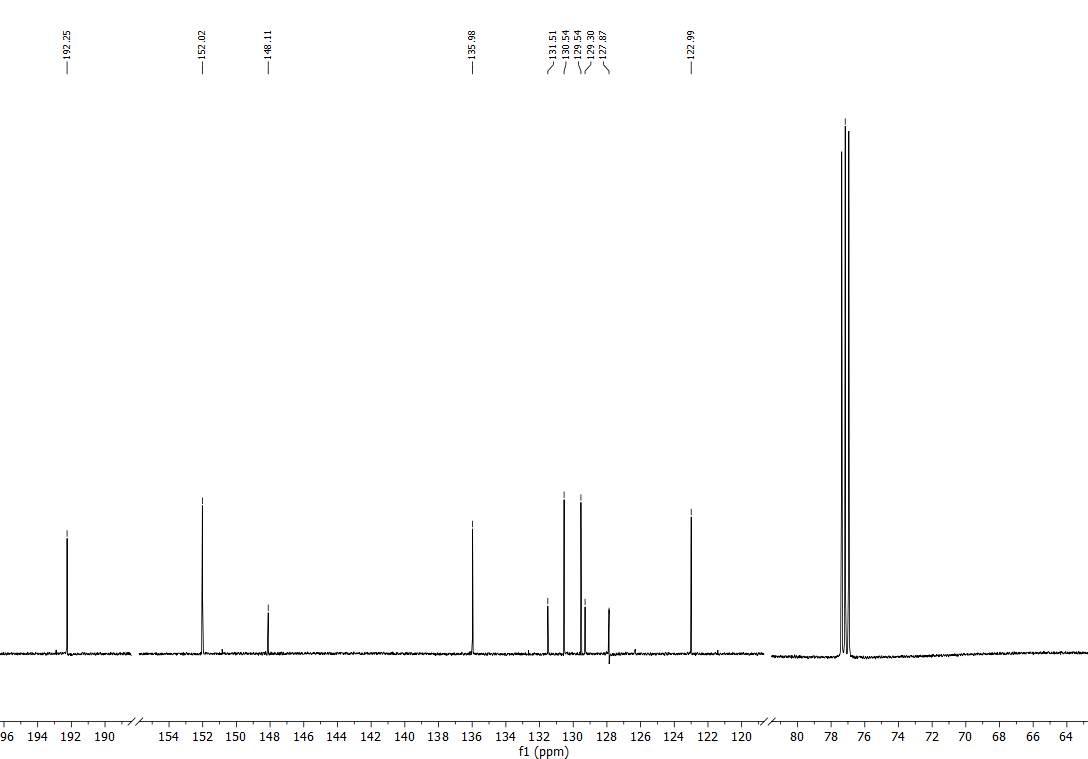
**

^1^H- (top) and ^13^C-NMR (bottom) spectra of 5-Bromoquinoline-8-carbaldehyde (**4a**) in CDCl_3_.

**
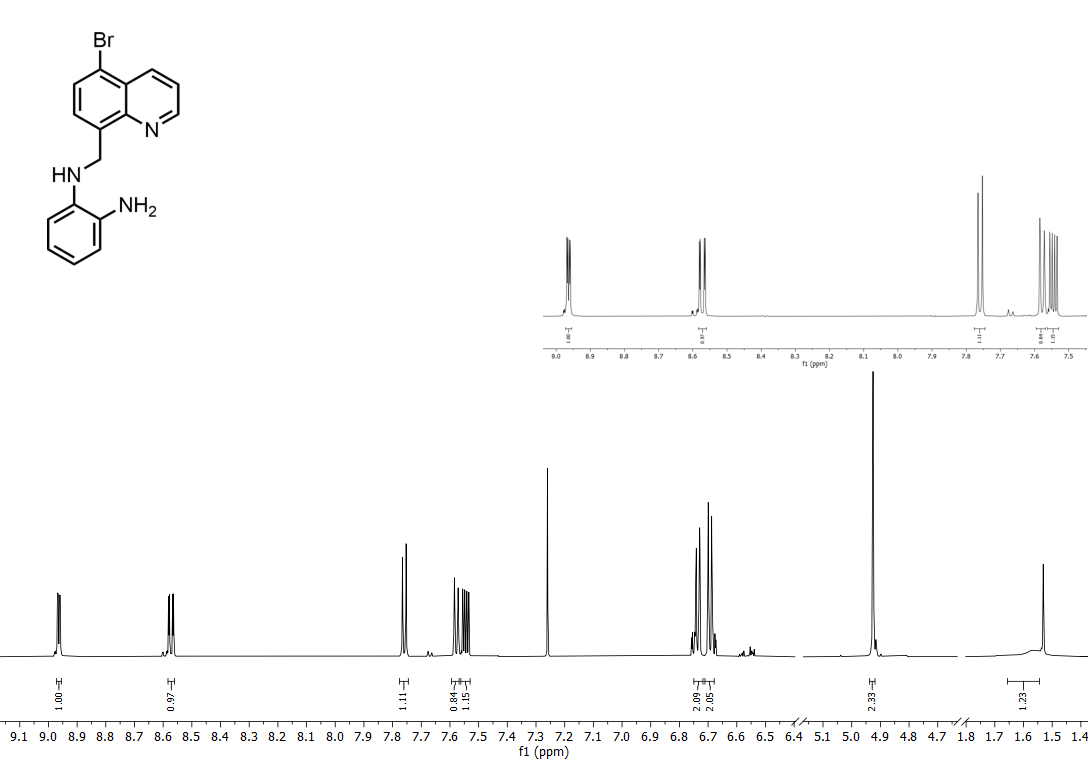
**

**
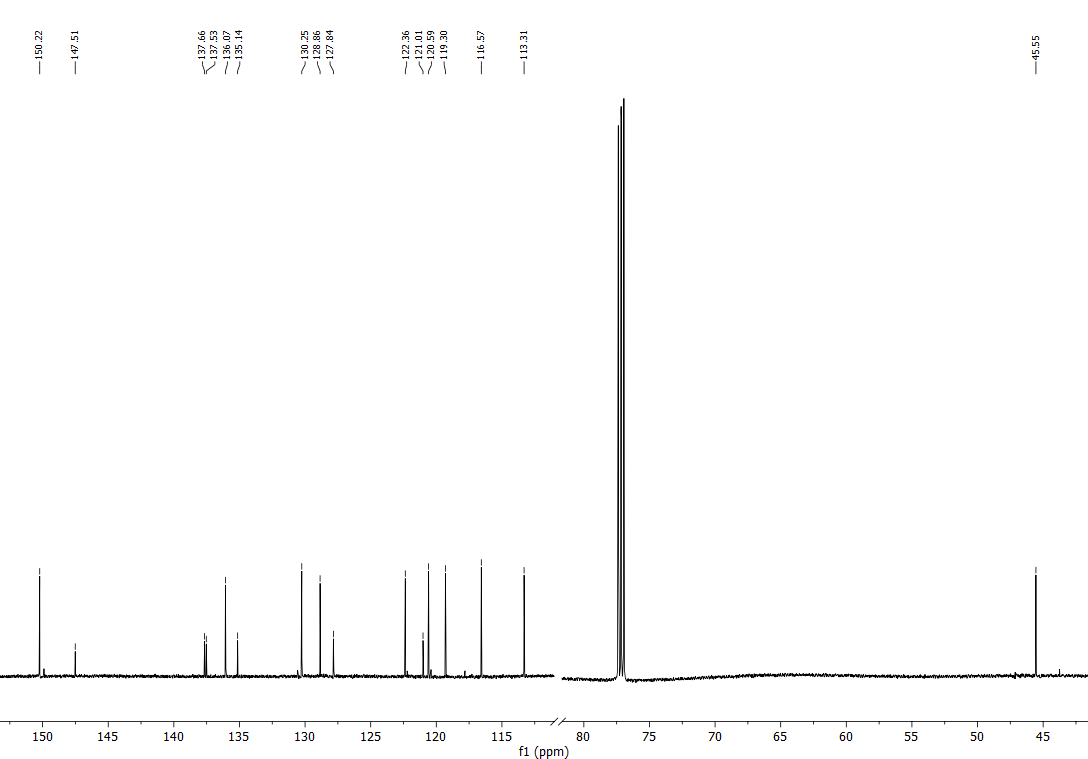
**

^1^H- (top) and ^13^C-NMR (bottom) spectra of *N*-[(5-Bromoquinolin-8-yl)methyl]benzene-1,2-diamine (**4b**) in CDCl_3_.

**
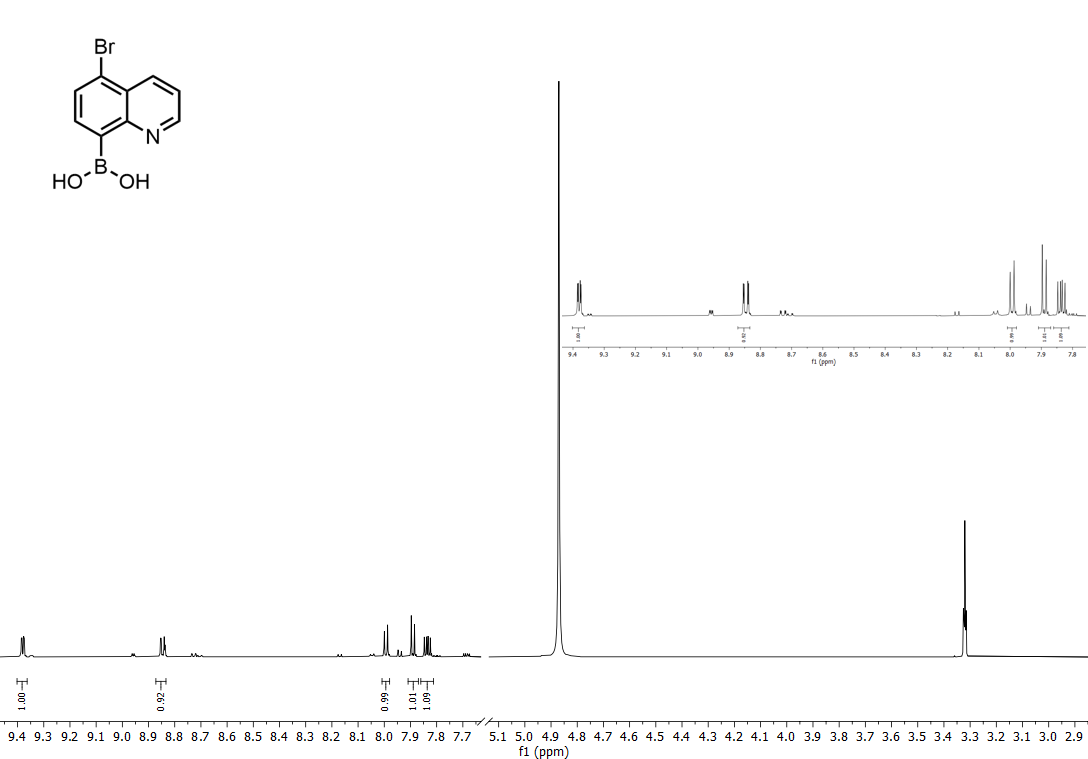
**

**
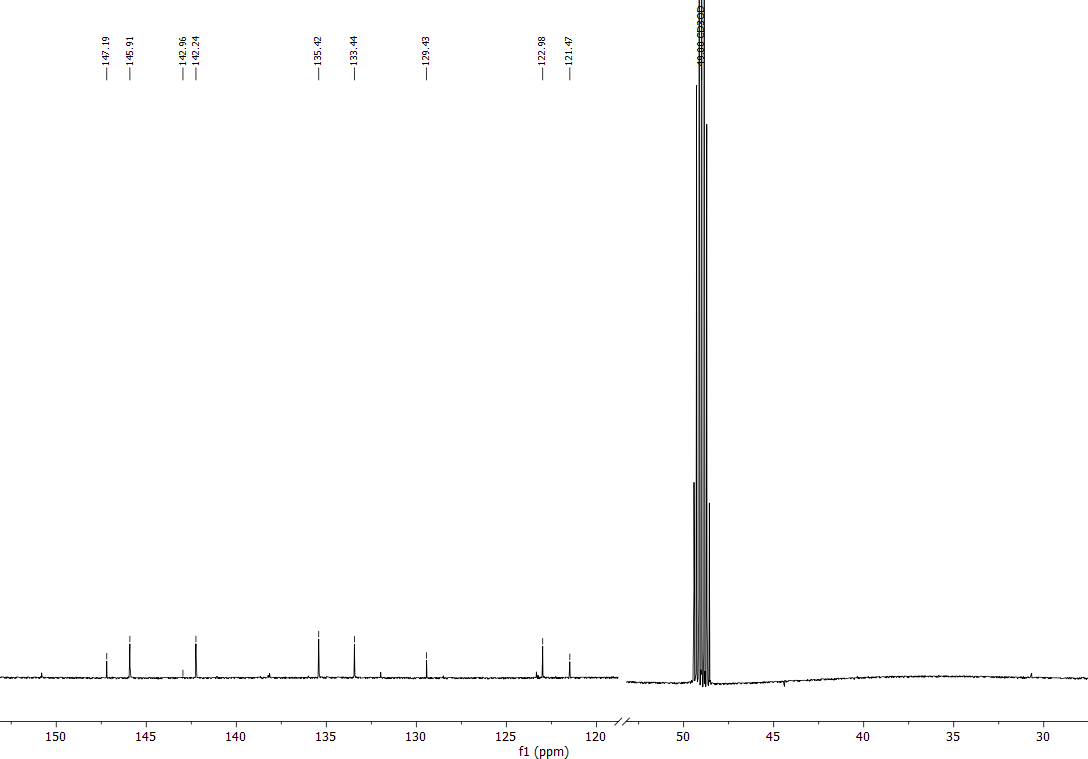
**

^1^H- (top) and ^13^C-NMR (bottom) spectra of 5-Bromoquinoline-8-boronic acid (**4c**) in CD_3_OD.

*
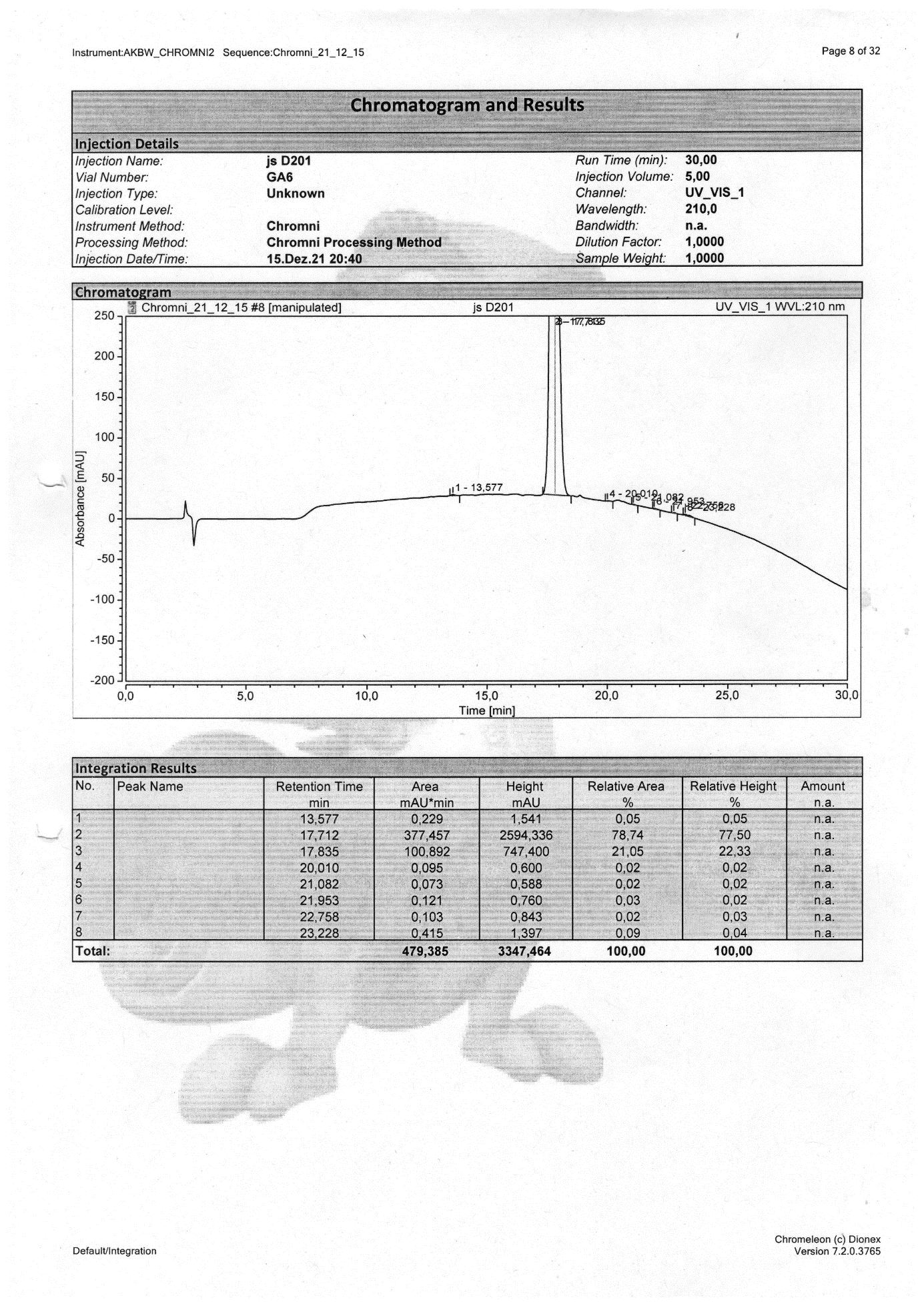
*

Purity (HPLC) of 5-Chloro-8-nitroquinoline (**2a**).

*
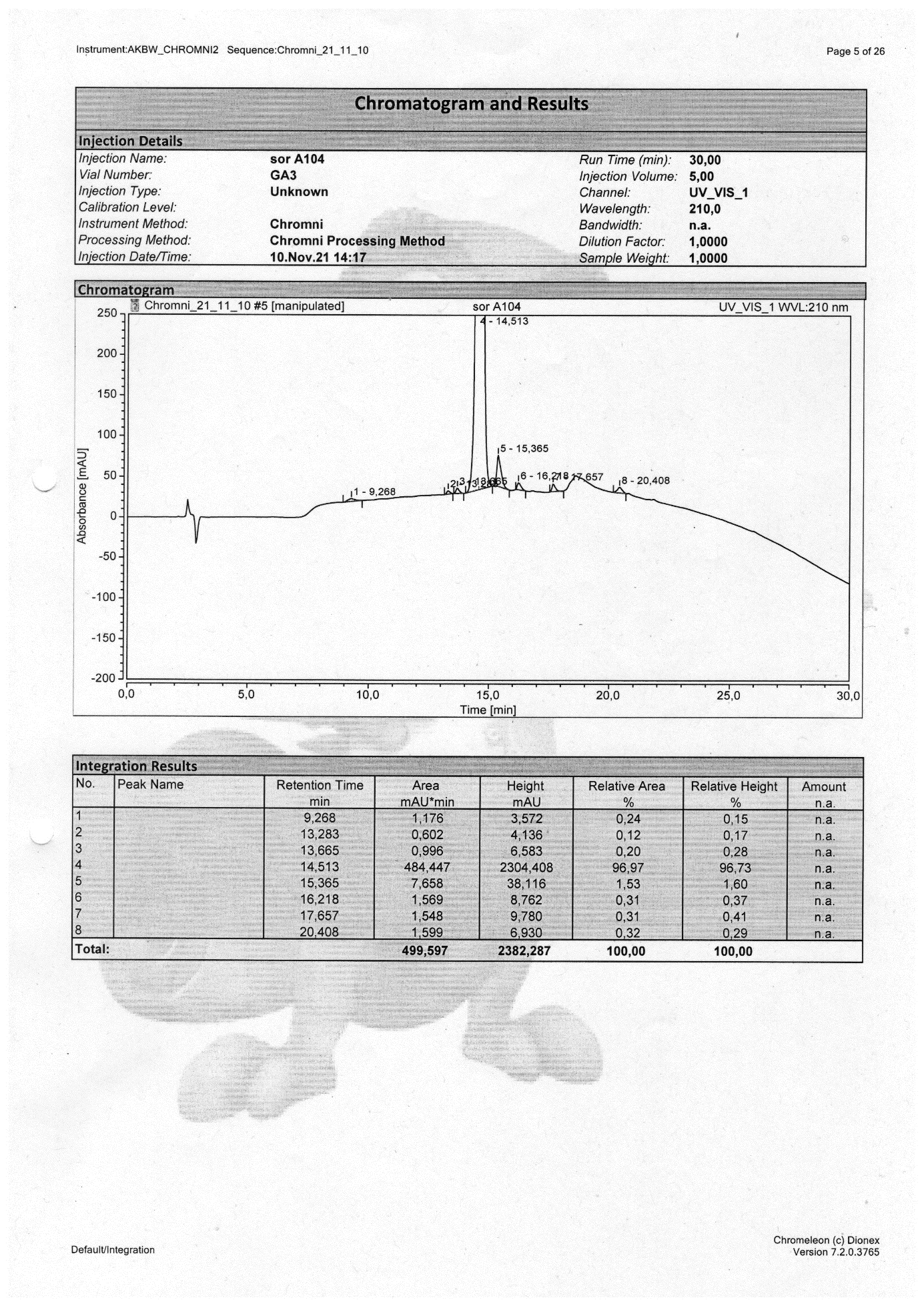
*

Purity (HPLC) of 5-Chloroquinoline-8-carboxylic acid (**2b**).

*
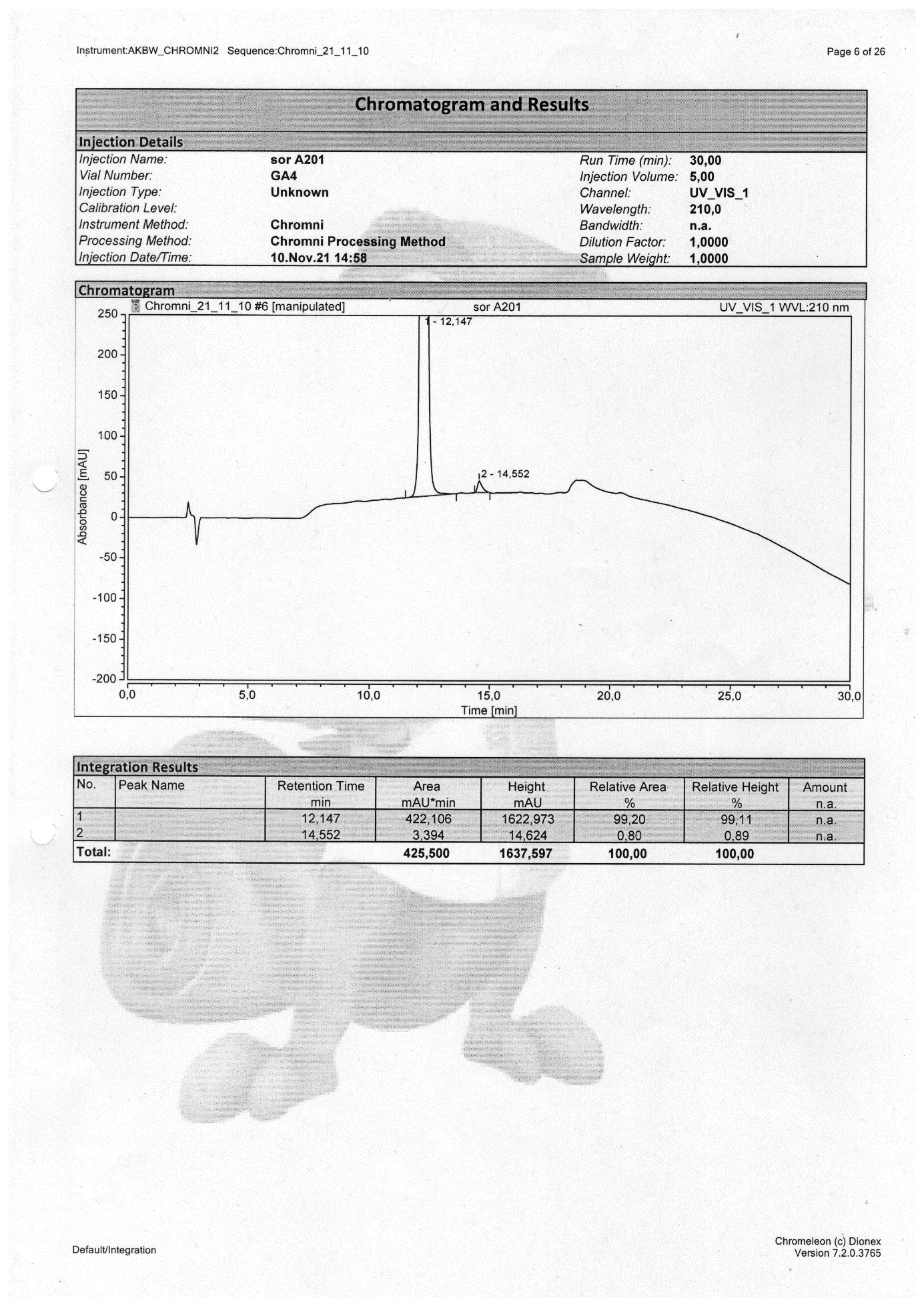
*

Purity (HPLC) of Methyl 5-chloroquinoline-8-carboxylate (**2c**).

*
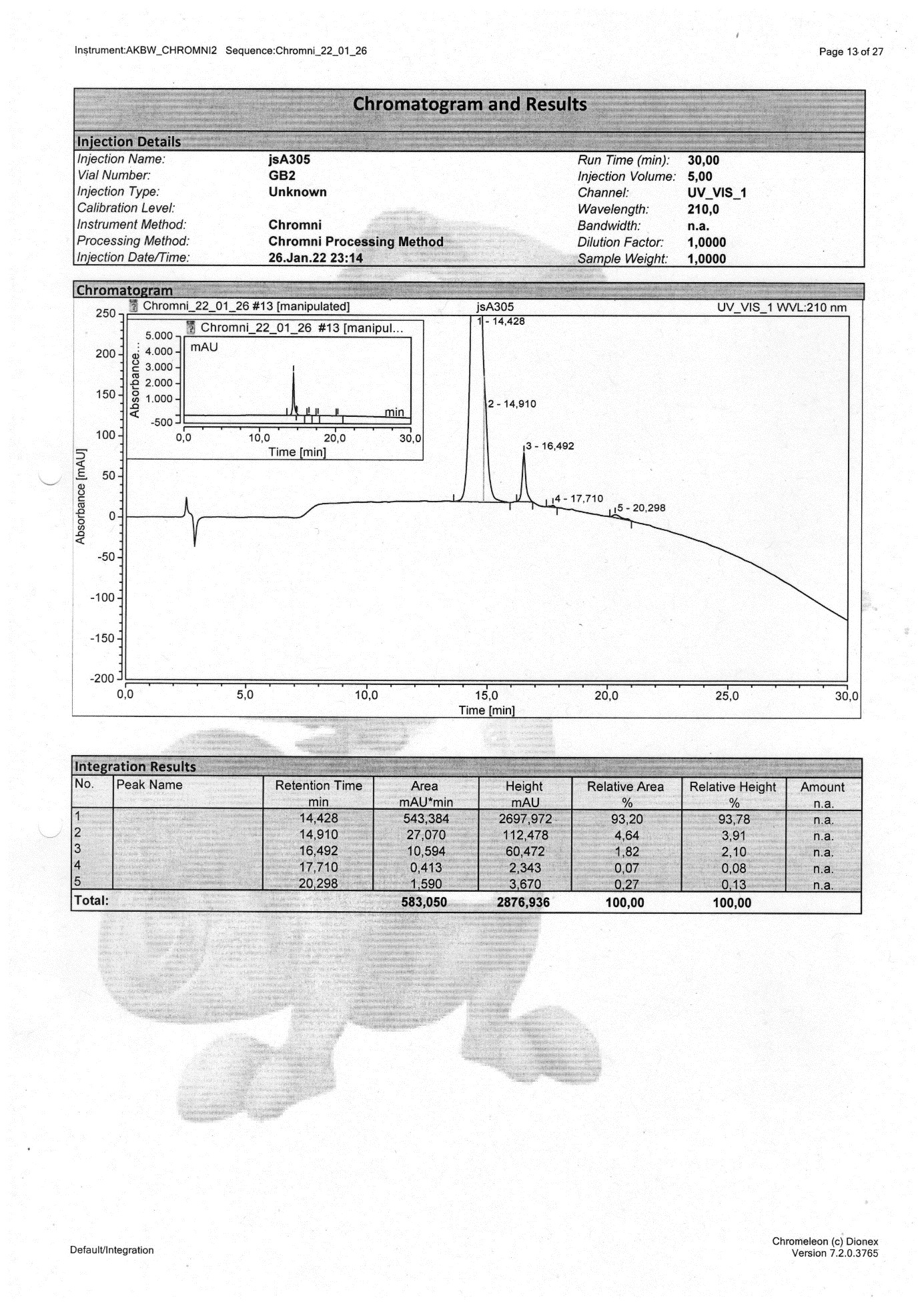
*

Purity (HPLC) of 5-Chloro-*N*-methylquinoline-8-carboxamide (**2d**).

*
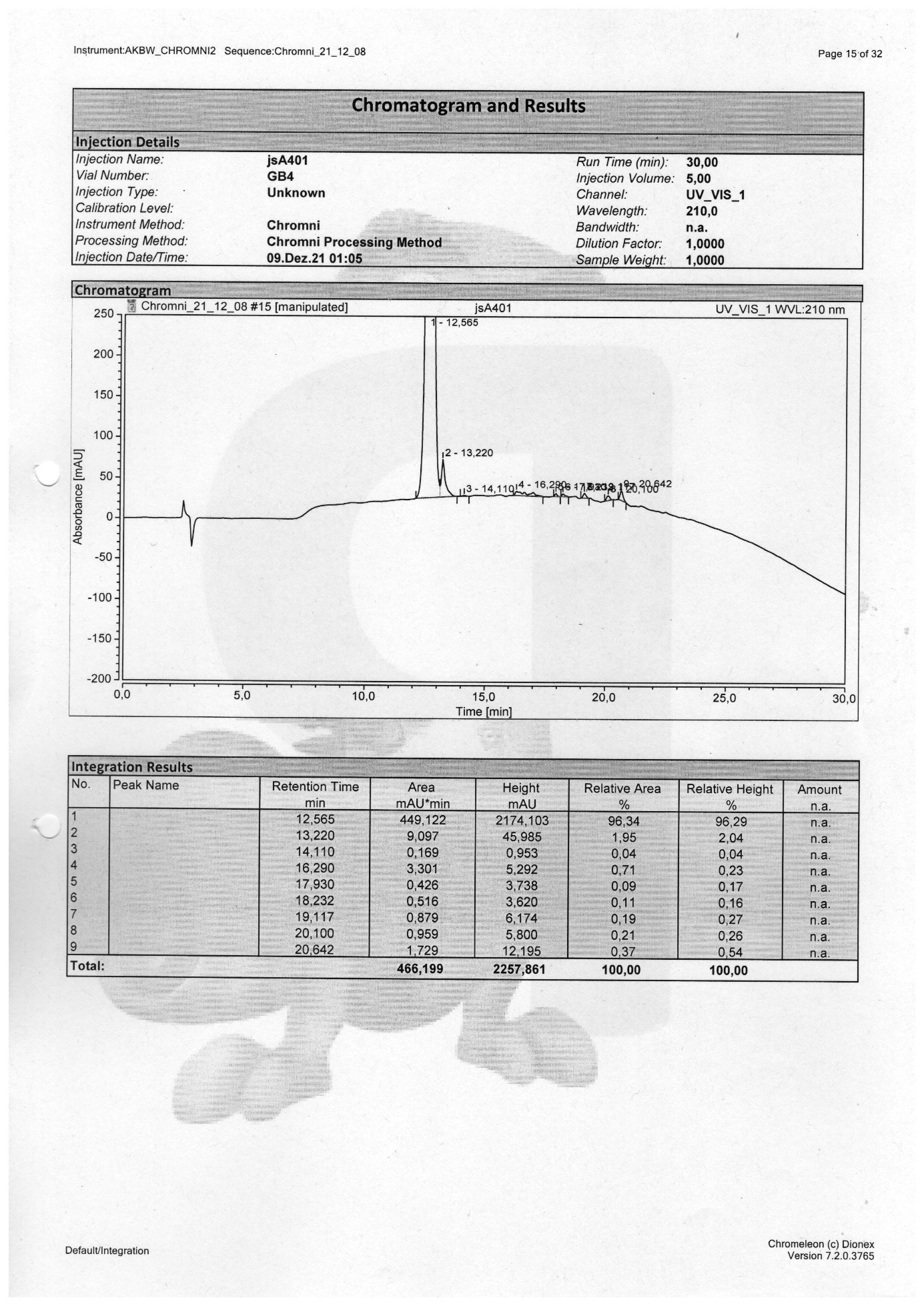
*

Purity (HPLC) of (5-Chloroquinolin-8-yl)methanol (**2e**).

*
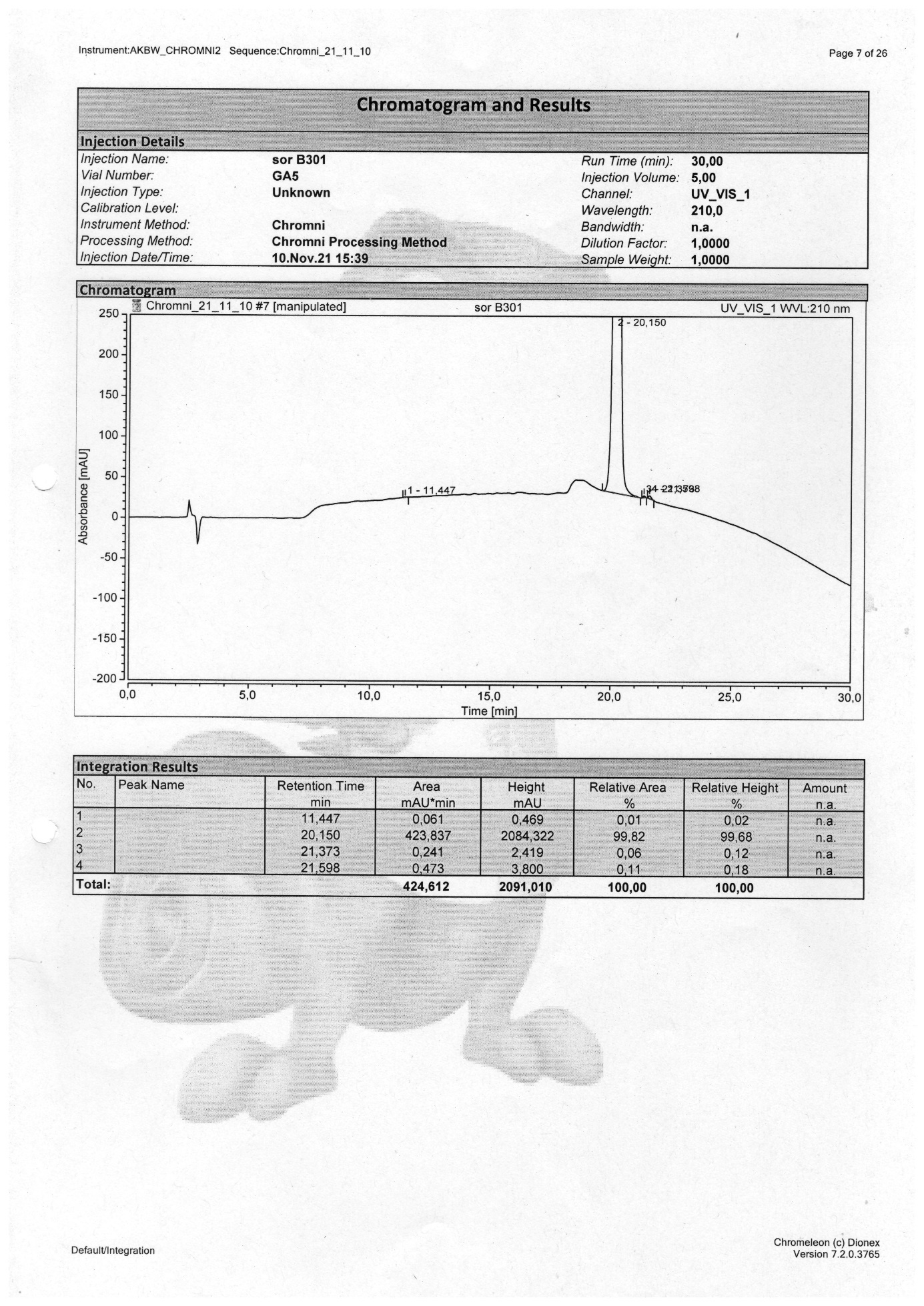
*

Purity (HPLC) of 5-Bromo-*N*-methylquinoline-8-amine (**3b**).

*
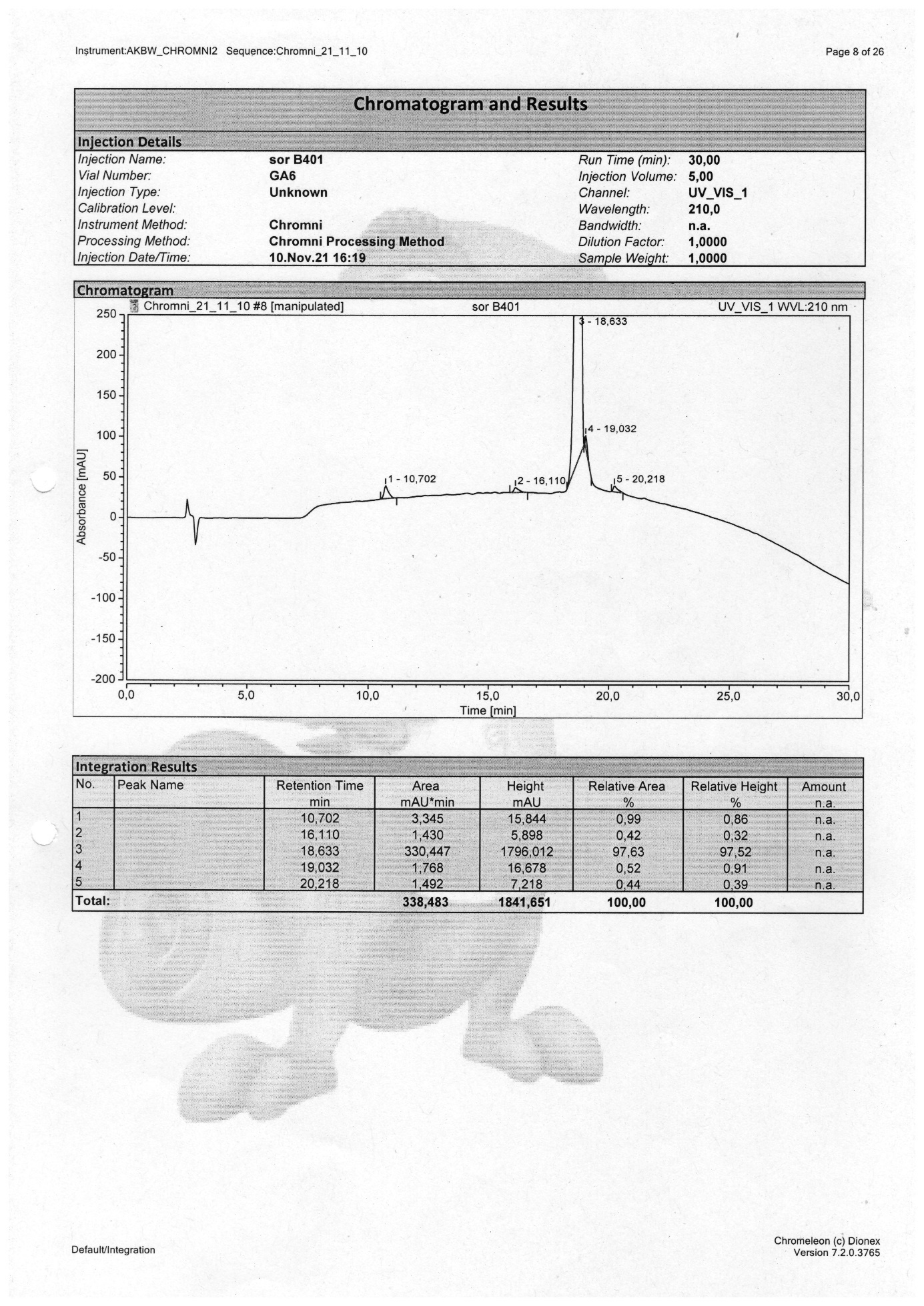
*

Purity (HPLC) of *N*-(5-Bromoquinolin-8-yl)formamid (**3c**).

*
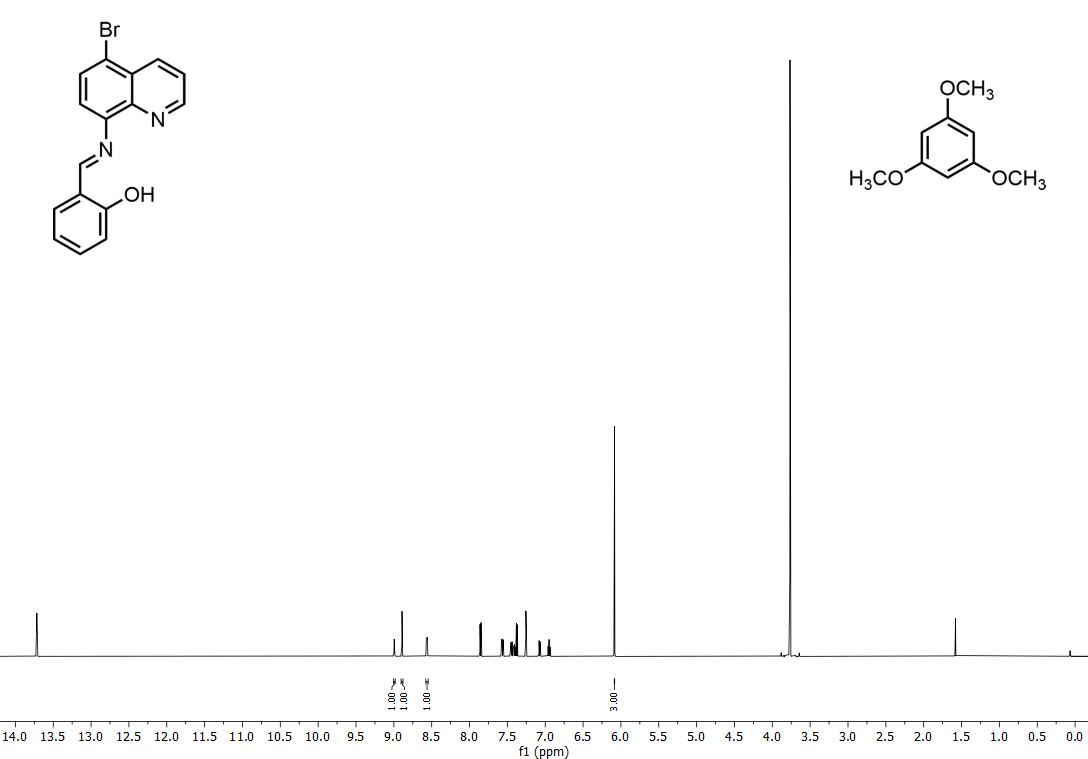
*

Quantitative ^1^H-NMR spectrum of *N*-(5-Bromoquinolin-8-yl)2-hydroxybenzaldimine (**3d)** in CDCl_3_ with 1,3,5-trimethoxybenzene as internal calibrant.

*
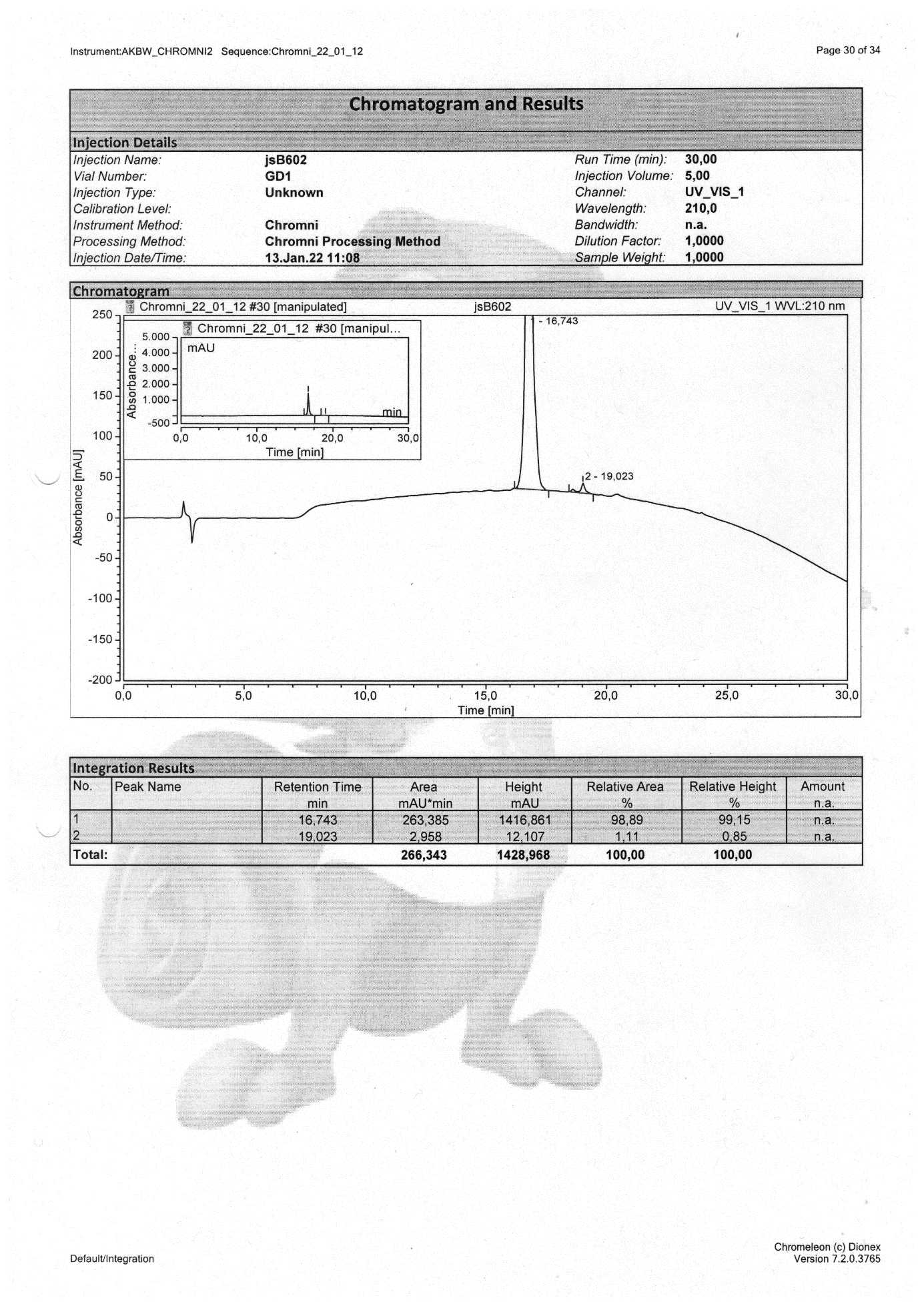
*

Purity (HPLC) of 1-(5-Bromoquinolin-8-yl)urea (**3e**).

*
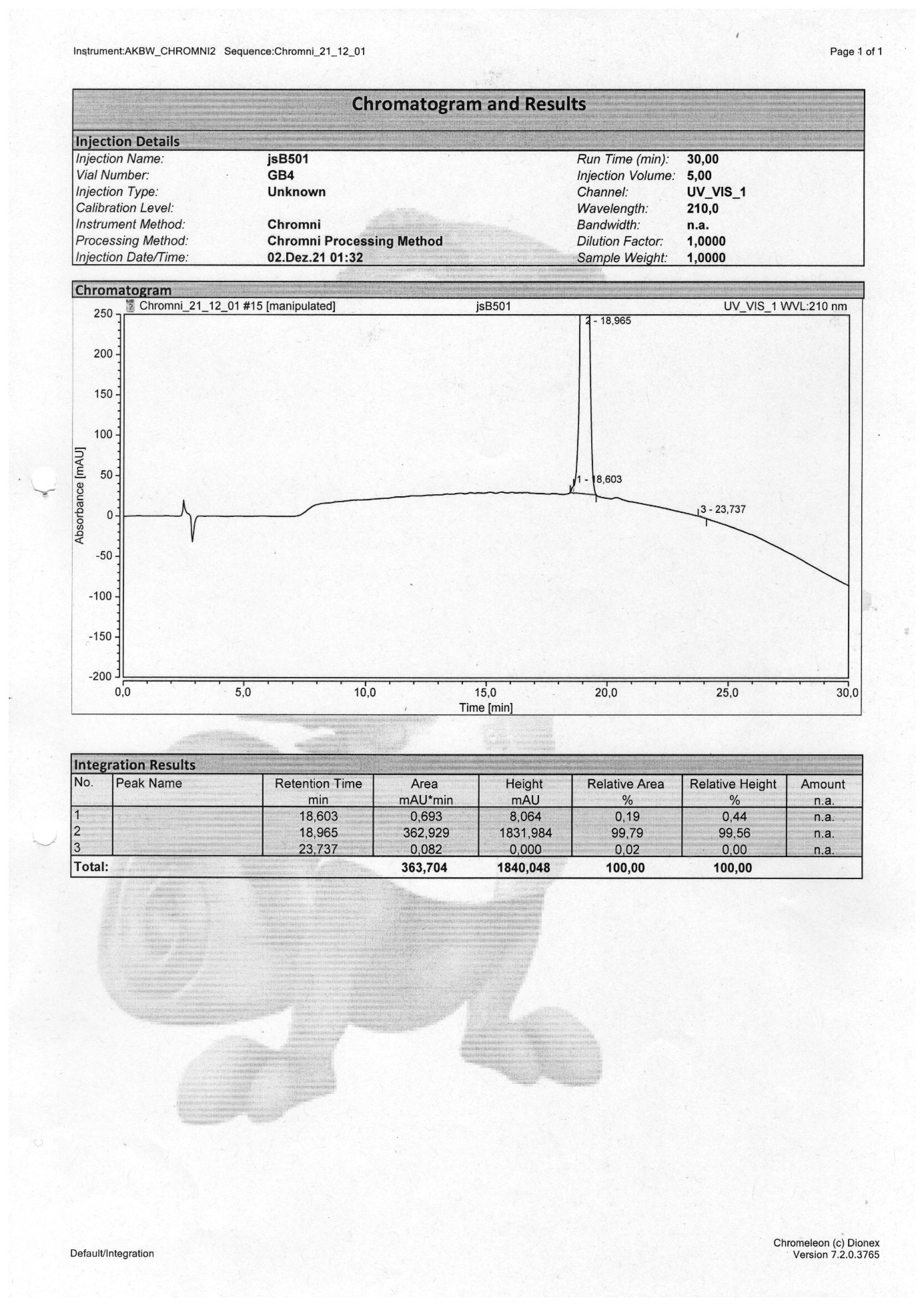
*Purity (HPLC) of *N*-(5-Bromoquinolin-8-yl)acetamid (**3f**).

*
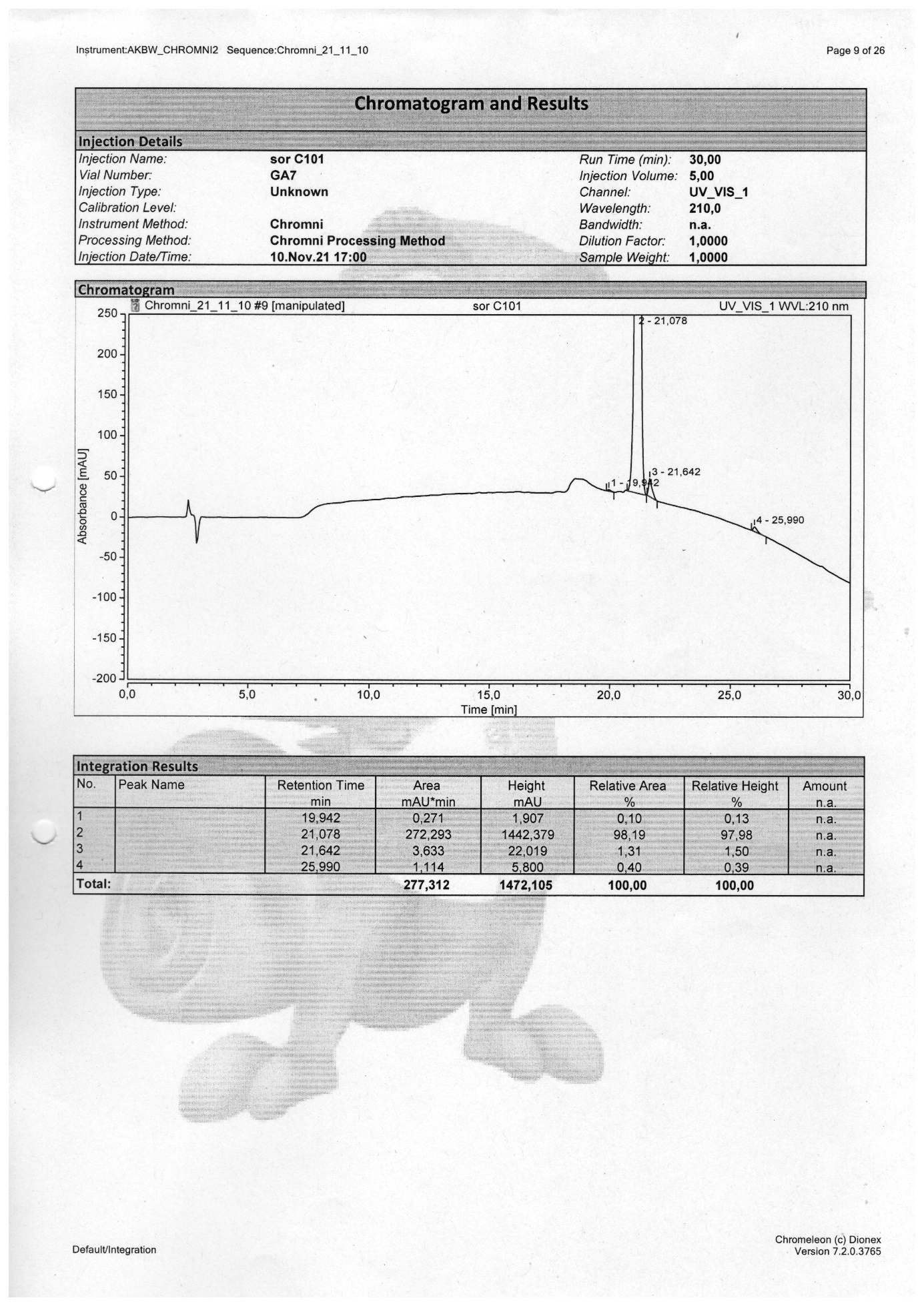
*Purity (HPLC) of 5-Bromo-8-iodoquinoline (**3g**).

*
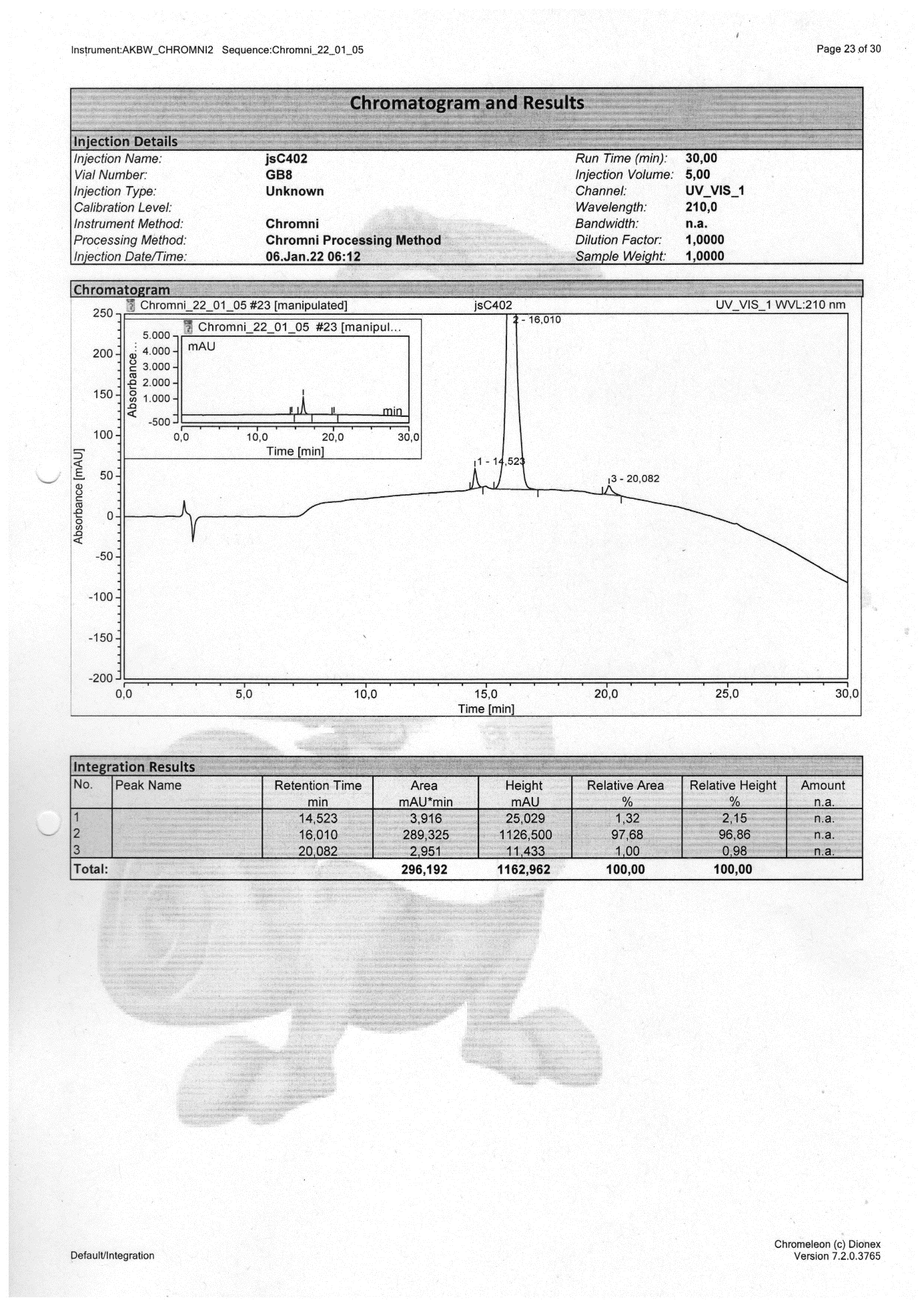
*

Purity (HPLC) of 5-Bromoquinoline-8-carbaldehyde (**4a**).

*
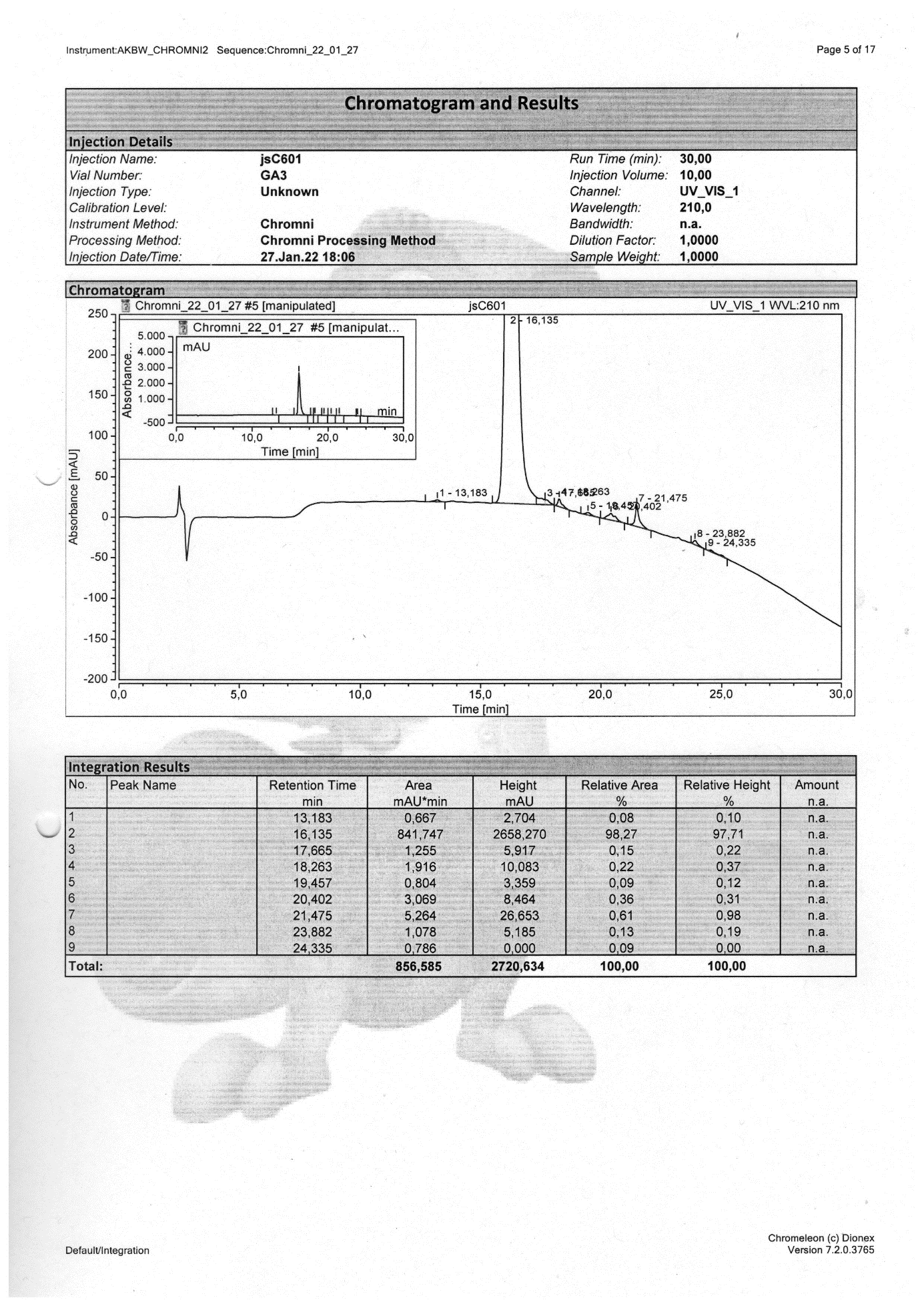
*

Purity (HPLC) of *N*-[(5-Bromoquinolin-8-yl)methyl]benzene-1,2-diamine (**4b**).

*
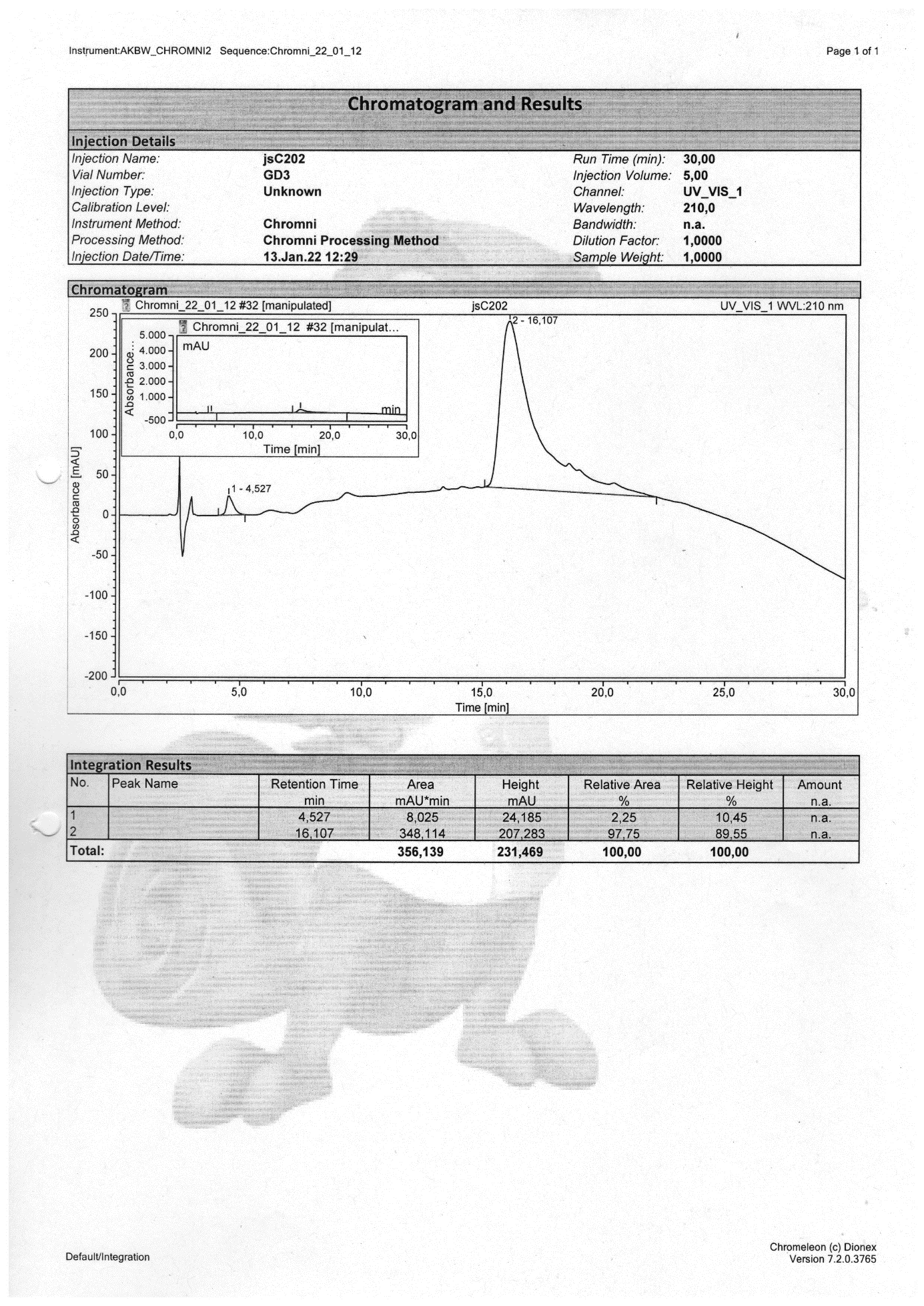
*

Purity (HPLC) of 5-Bromoquinoline-8-boronic acid (**4c**).

| SI Table 1: Fitting parameters for linear regression ln (c) = ln (c_0_) * -k*t ± SE. | | |
| --- | --- | --- |
|  | UV Vis data | NMR data |
| ln (c_0_) | 0.02008 ± 0.00118 | -1.50429 ± 0.03877 |
| k | 0.01756 ± 6.66571E-5 min^-1^ | 0.01792 ± 8.33432E-4 min^-1^ |
|  |  |  |
| coefficient of determination (R^2^) | 0.9996 | 0.9893 |
| Half-life t_1/2_ = ln (2) / k | ~ 39.47 min | ~38.68 min |


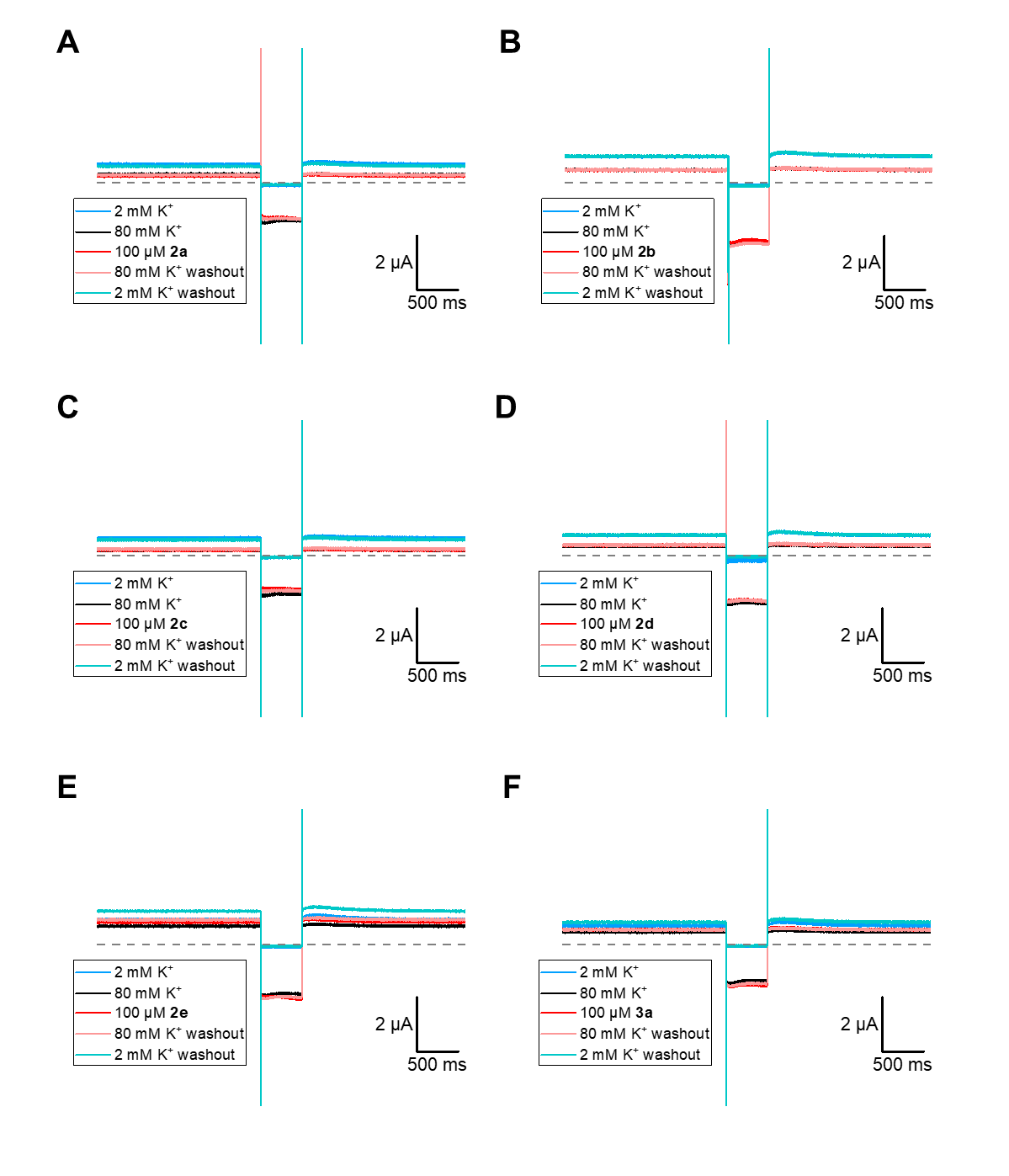


TEVC sample traces of compounds **2a**-**3a** resulting from repetitive pulse application from 0 mV to -100 mV (see materials and methods) at hK_2P_18.1 expressing *Xenopus* *laevis* oocytes. For each compound an overlay of the last sweep in presence of 2 mM K^+^ buffer (sweep 15, blue), 80 mM K^+^ buffer (sweep 35, black), 100 µM compound (sweep 65, red), 80 mM K^+^ washout (sweep 85, light red) and 2 mM K^+^ washout (sweep 100, cyan) is displayed. Data for ion channel modulation by the compound (I_c_) and compound washout (I_w_) were determined by currents recorded at -100 mV.


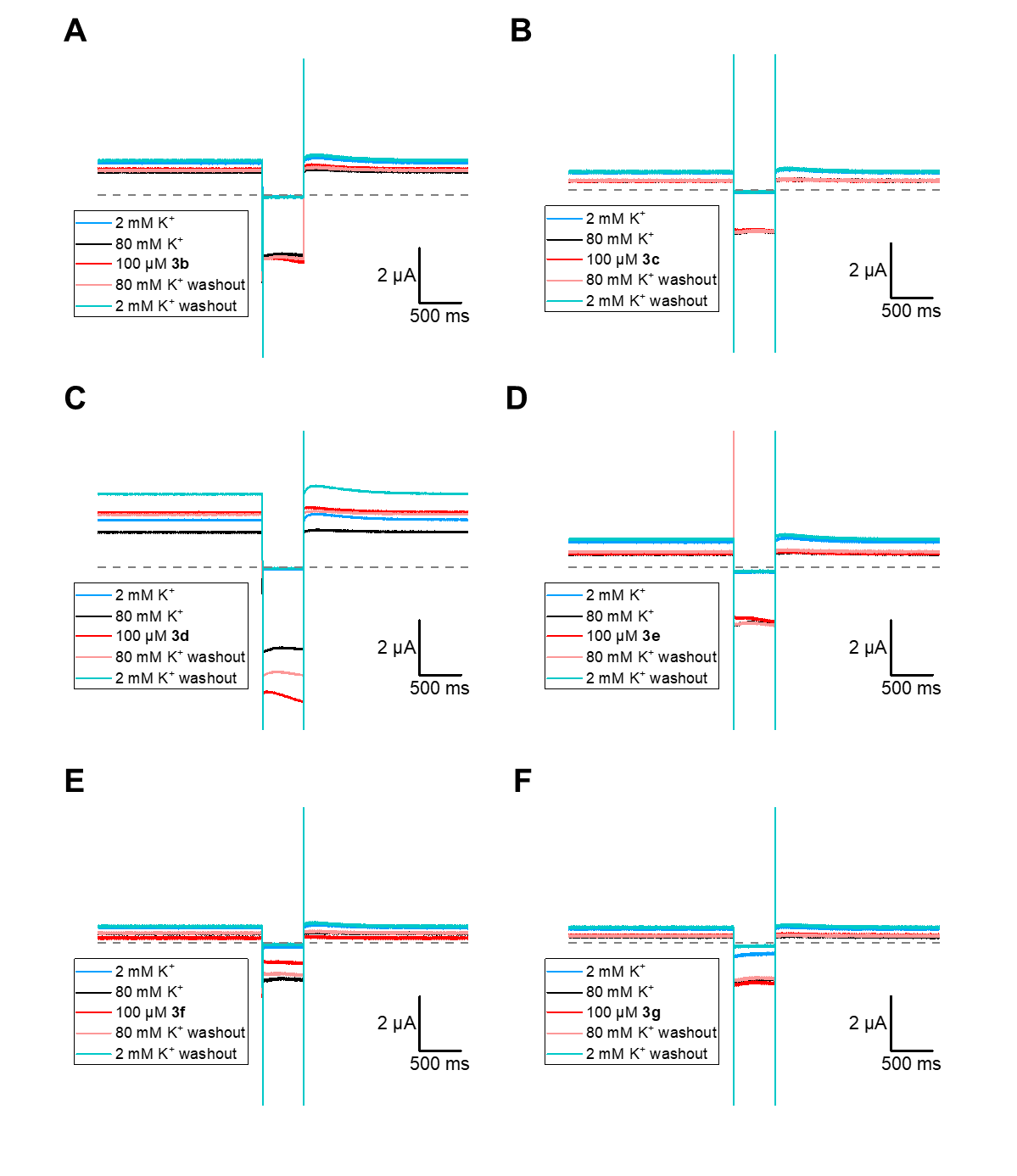


TEVC sample traces of compounds **3b**-**3g** resulting from repetitive pulse application from 0 mV to -100 mV (see materials and methods) at hK_2P_18.1 expressing *Xenopus* *laevis* oocytes. For each compound an overlay of the last sweep in presence of 2 mM K^+^ buffer (sweep 15, blue), 80 mM K^+^ buffer (sweep 35, black), 100 µM compound (sweep 65, red), 80 mM K^+^ washout (sweep 85, light red) and 2 mM K^+^ washout (sweep 100, cyan) is displayed. Data for ion channel modulation by the compound (I_c_) and compound washout (I_w_) were determined by currents recorded at -100 mV.


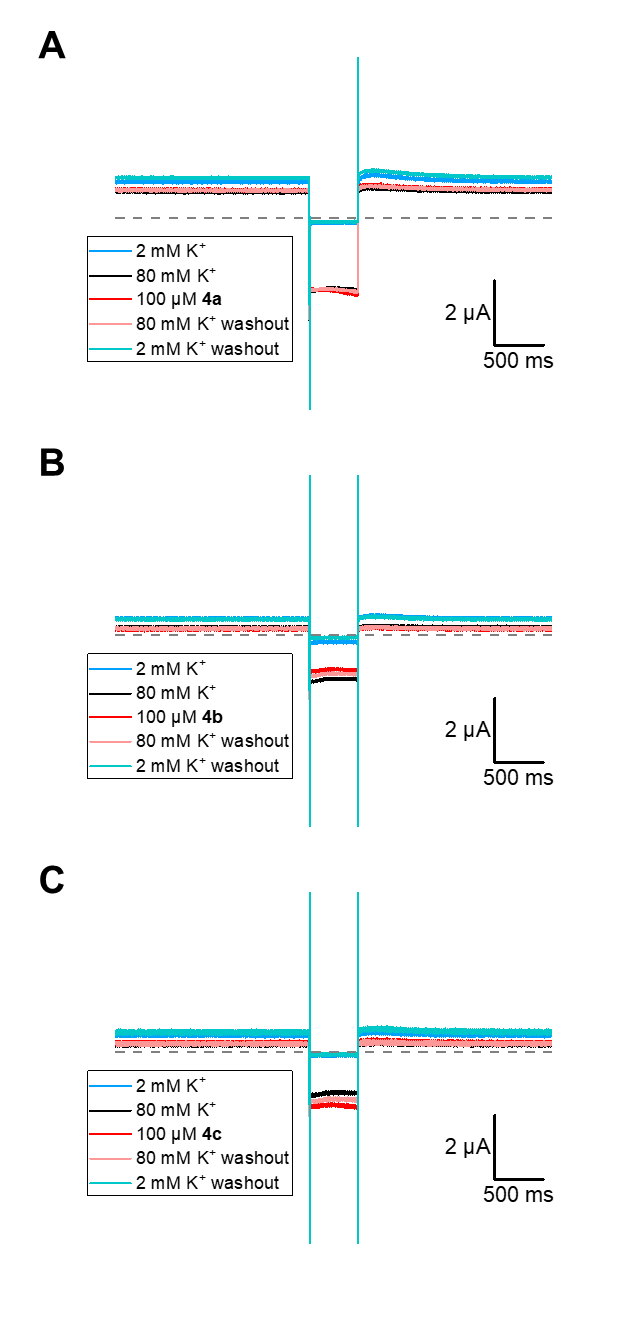


TEVC sample traces of compounds **4a**-**4c** resulting from repetitive pulse application from 0 mV to -100 mV (see materials and methods) at hK_2P_18.1 expressing *Xenopus* *laevis* oocytes. For each compound an overlay of the last sweep in presence of 2 mM K^+^ buffer (sweep 15, blue), 80 mM K^+^ buffer (sweep 35, black), 100 µM compound (sweep 65, red), 80 mM K^+^ washout (sweep 85, light red) and 2 mM K^+^ washout (sweep 100, cyan) is displayed. Data for ion channel modulation by the compound (I_c_) and compound washout (I_w_) were determined by currents recorded at -100 mV.
