# Supplementary material for: Evaluation of Novel Cloxyquin Analogs for K2P18.1 Channel Modulation
Source: Arch Pharm (Weinheim). 2026 Jul 15;359(7):e70310. doi: 10.1002/ardp.70310 (PMC13373494; doi:10.1002/ardp.70310)
Supplement: Supplementary file 2 — Supporting File 2 [file ARDP-359-e70310-s001.doc]

**Supplemental Material: Novel Compounds and Biological Screening Results**

**Evaluation of novel cloxyquin analogs for K_2P_18.1 channel modulation**

Jasmin Sörgel^1)^, Marcel Kloth^2)^, Henning Klaasen^2)^, Sven G. Meuth^3)^, Thomas Budde^4)^, Bart Jan Ravoo^2),5)^, Julian A. Schreiber^1)*^

1. Institute of Pharmaceutical and Medicinal Chemistry, University of Münster, Corrensstraße 48, 48149 Münster, Germany.
2. Organisch-Chemisches Institut, University of Münster, Corrensstraße 36, 48149 Münster, Germany.
3. Department of Neurology, University Hospital Münster, 48149 Münster, Germany.
4. Institute of Physiology I, University of Münster, 48149, Münster, Germany.
5. Center for Soft Nanoscience, University of Münster, Busso-Peus-Straße 10, 48149 Münster, Germany.

Corresponding author – full address: Dr. Julian Alexander Schreiber, Corrensstraße 48, D-48149 Münster, Tel.: +49-251-8333372, j.schreiber@uni-muenster.de.

| **Compound No.** | **InChI** | **Biological Activity (% activity at 100 µM)^a^** |
| --- | --- | --- |
| **2a** | InChI=1S/C9H5ClN2O2/c10-7-3-4-8(12(13)14)9-6(7)2-1-5-11-9/h1-5H | 103.2 ± 7.1 |
| **2b** | InChI=1S/C10H6ClNO2/c11-8-4-3-7(10(13)14)9-6(8)2-1-5-12-9/h1-5H,(H,13,14) | 98.3 ± 1.6 |
| **2c** | InChI=1S/C11H8ClNO2/c1-15-11(14)8-4-5-9(12)7-3-2-6-13-10(7)8/h2-6H,1H3 | 93.0 ± 4.2 |
| **2d** | InChI=1S/C11H9ClN2O/c1-13-11(15)8-4-5-9(12)7-3-2-6-14-10(7)8/h2-6H,1H3,(H,13,15) | 96.0 ± 0.6 |
| **2e** | InChI=1S/C10H8ClNO/c11-9-4-3-7(6-13)10-8(9)2-1-5-12-10/h1-5,13H,6H2 | 109.2 ± 4.1 |
| **3a** | InChI=1S/C9H7BrN2/c10-7-3-4-8(11)9-6(7)2-1-5-12-9/h1-5H,11H2 | 113.2 ± 1.4 |
| **3b** | InChI=1S/C10H9BrN2/c1-12-9-5-4-8(11)7-3-2-6-13-10(7)9/h2-6,12H,1H3 | 109.5 ± 0.7 |
| **3c** | InChI=1S/C10H7BrN2O/c11-8-3-4-9(13-6-14)10-7(8)2-1-5-12-10/h1-6H,(H,13,14) | 96.6 ± 0.8 |
| **3d** | InChI=1S/C16H11BrN2O/c17-13-7-8-14(16-12(13)5-3-9-18-16)19-10-11-4-1-2-6-15(11)20/h1-10,20H/b19-10+ | 159.8 ± 9.8 |
| **3e** | InChI=1S/C10H8BrN3O/c11-7-3-4-8(14-10(12)15)9-6(7)2-1-5-13-9/h1-5H,(H3,12,14,15) | 93.3 ± 1.6 |
| **3f** | InChI=1S/C11H9BrN2O/c1-7(15)14-10-5-4-9(12)8-3-2-6-13-11(8)10/h2-6H,1H3,(H,14,15) | 64.6 ± 7.0 |
| **3g** | InChI=1S/C9H5BrIN/c10-7-3-4-8(11)9-6(7)2-1-5-12-9/h1-5H | 107.0 ± 5.3 |
| **4a** | InChI=1S/C10H6BrNO/c11-9-4-3-7(6-13)10-8(9)2-1-5-12-10/h1-6H | 107.0 ± 1.0 |
| **4b** | InChI=1S/C16H14BrN3/c17-13-8-7-11(16-12(13)4-3-9-19-16)10-20-15-6-2-1-5-14(15)18/h1-9,20H,10,18H2 | 80.7 ± 3.1 |
| **4c** | InChI=1S/C9H7BBrNO2/c11-8-4-3-7(10(13)14)9-6(8)2-1-5-12-9/h1-5,13-14H | 127.9 ± 6.3 |

^a^ Two-electrode voltage clamp (TEVC) recordings as previously described in Schreiber JA, Derksen A, Goerges G, Schütte S, Sörgel J, Kiper AK, Strutz-Seebohm N, Ruck T, Meuth SG, Decher N, Seebohm G. Cloxyquin activates hTRESK by allosteric modulation of the selectivity filter. Commun Biol. 2023 Jul 18;6(1):745. doi: 10.1038/s42003-023-05114-4. PMID: 37464013; PMCID: PMC10354012. .
